# Defining the ‘HoneySweet’ insertion event utilizing NextGen sequencing and a de novo genome assembly of plum (*Prunus domestica*)

**DOI:** 10.1038/s41438-020-00438-2

**Published:** 2021-01-01

**Authors:** Ann M. Callahan, Tetyana N. Zhebentyayeva, Jodi L. Humann, Christopher A. Saski, Kelsey D. Galimba, Laura L. Georgi, Ralph Scorza, Dorrie Main, Christopher D. Dardick

**Affiliations:** 1 USDA-ARS, Appalachian Fruit Research Station, Kearneysville, WV 25430 USA; 2 The Schatz Center for Tree Molecular Genetics, Department of Ecosystem Science and Management, The Pennsylvania State University, University Park, PA 16802 USA; 3 Department of Horticulture, Washington State University, Pullman, WA 99164 USA; 4 Plant and Environmental Sciences Department, Clemson University, Clemson, SC 29634 USA; 5 Department of Genetics and Biochemistry, Clemson University, Clemson, SC 29634 USA

**Keywords:** Molecular engineering in plants, Plant breeding

## Abstract

‘HoneySweet’ plum (*Prunus domestica*) is resistant to *Plum pox potyvirus*, through an RNAi-triggered mechanism. Determining the precise nature of the transgene insertion event has been complicated due to the hexaploid genome of plum. DNA blots previously indicated an unintended hairpin arrangement of the *Plum pox potyvirus* coat protein gene as well as a multicopy insertion event. To confirm the transgene arrangement of the insertion event, ‘HoneySweet’ DNA was subjected to whole genome sequencing using Illumina short-read technology. Results indicated two different insertion events, one containing seven partial copies flanked by putative plum DNA sequence and a second with the predicted inverted repeat of the coat protein gene driven by a double 35S promoter on each side, flanked by plum DNA. To determine the locations of the two transgene insertions, a phased plum genome assembly was developed from the commercial plum ‘Improved French’. A subset of the scaffolds (2447) that were >10 kb in length and representing, >95% of the genome were annotated and used for alignment against the ‘HoneySweet’ transgene reads. Four of eight matching scaffolds spanned both insertion sites ranging from 157,704 to 654,883 bp apart, however we were unable to identify which scaffold(s) represented the actual location of the insertion sites due to potential sequence differences between the two plum cultivars. Regardless, there was no evidence of any gene(s) being interrupted as a result of the insertions. Furthermore, RNA-seq data verified that the insertions created no new transcriptional units and no dramatic expression changes of neighboring genes.

## Introduction

‘HoneySweet’ plum (*Prunus domestica*) is highly resistant to the devastating disease, Sharka, for which the causal agent is *Plum pox potyvirus* (PPV)^1,2^. ‘HoneySweet’ is derived from a ‘Bluebyrd’ x unknown pollen parent seed that had been transformed with a PPV coat protein gene (CP), driven by the 35S promoter^3^. Standard molecular analyses including DNA blots, RNA blots, and protein blots, suggested that there were 3–4 copies of the CP gene inserted, yet very low levels of CP RNA and no detectable CP^3^. Further analyses deduced that ‘HoneySweet’ was resistant to PPV through an RNAi mediated response^4^. The introduced CP was methylated, transcription rates were high, but RNA was undetectable outside of the nucleus and PPV RNA was not detectable after challenge inoculations.

Further DNA blotting experiments suggested that there was a rearrangement of the inserted DNA resulting in a predicted hairpin of two CP genes as well as a separate multicopy arrangement of the transgenes^4^. To verify this, a BAC library was constructed from ‘HoneySweet’ DNA. Two BAC clones with NPT sequence but not containing the CP were isolated along with a single clone that contained the CP gene. Upon sequencing the CP clone and confirmation by PCR, it was found that this fragment contained an inverted duplication resulting in a tail-to-tail arrangement of the CP gene as well as a double 35S promoter sequence driving each of the copies. An incomplete 3′UTR was situated between the two copies of the CP gene resulting in a short intervening region that was not duplicated. This CP hairpin fragment was transformed back into plum and plants were tested for resistance. Four independent lines (out of 8) showed high levels of resistance through three cycles of dormancy. These results confirmed that this unintended hairpin could be responsible for the resistance to PPV of ‘HoneySweet’^5^.

To more precisely define the insertion event(s) within ‘Honeysweet’ and their potential impacts, NextGen sequencing was used to sequence ‘HoneySweet’^6^. In addition, the hexaploid *Prunus domestica* ‘Improved French’ was sequenced and phased. The phased genome was then used to identify the insertion sites within the ‘HoneySweet’ genome. Comprehensive RNA-seq analyses were performed to evaluate if the insertion event(s) resulted in the production of new transcripts or altered the expression of neighboring genes

## Results

### Plum genome assembly and annotation

‘Improved French’, from which a great majority of the commercial production of dried plums (prunes) is derived, was chosen to provide a phased genome sequence, such that all six copies of each chromosome would be represented. This was performed by NRGene using second-generation sequencing resulting in 210× coverage and third-generation sequencing resulting in 55× coverage. The data were assembled into 27,870 scaffolds representing a genome of 1,399,321,220 bases (Table 1). Using the number of conserved genes Benchmarking Universal Single-Copy Orthologs (BUSCO)^7^ to evaluate the completeness and accuracy of the genome assembly, 1385 or 96.2% of all the genes were found to be complete, and of which 1318 were duplicated. In addition, there were 9 fragmented genes (0.6%) and 46 missing genes (3.8%). Table 1 presents summaries of various aspects of the genome. The assembled genome was then annotated resulting in 130,866 gene models and is available on Genome Database for Rosaceae (GDR), https://www.rosaceae.org/^8^.

**Table 1:** Phased plum genome description

	Phased
Total scaffolds^a^	27,870
Assembly size	1,399,321,220
Gaps size	27,033,790
Gaps, %	1.93
N50	1,627,960
N50 #sequences	238
N90	128,064
N90 #sequences	1152
MAX	8,330,377
Complete BUSCOs	1385 (96.2%)
Complete BUSCOs single copy	67 (4.7%)
Complete BUSCOS—duplicated	1318 (91.5%)
Fragmented BUSCOs	9 (0.6%)
Missing BUSCOs	46 (3.2%)
Total BUSCO groups searched	1440

*BUSCO* Benchmarking Universal Single-Copy Orthologs

^a^The contigs composing the scaffolds are gapless

### NextGen sequence of ‘HoneySweet’

**Fig. 1:**
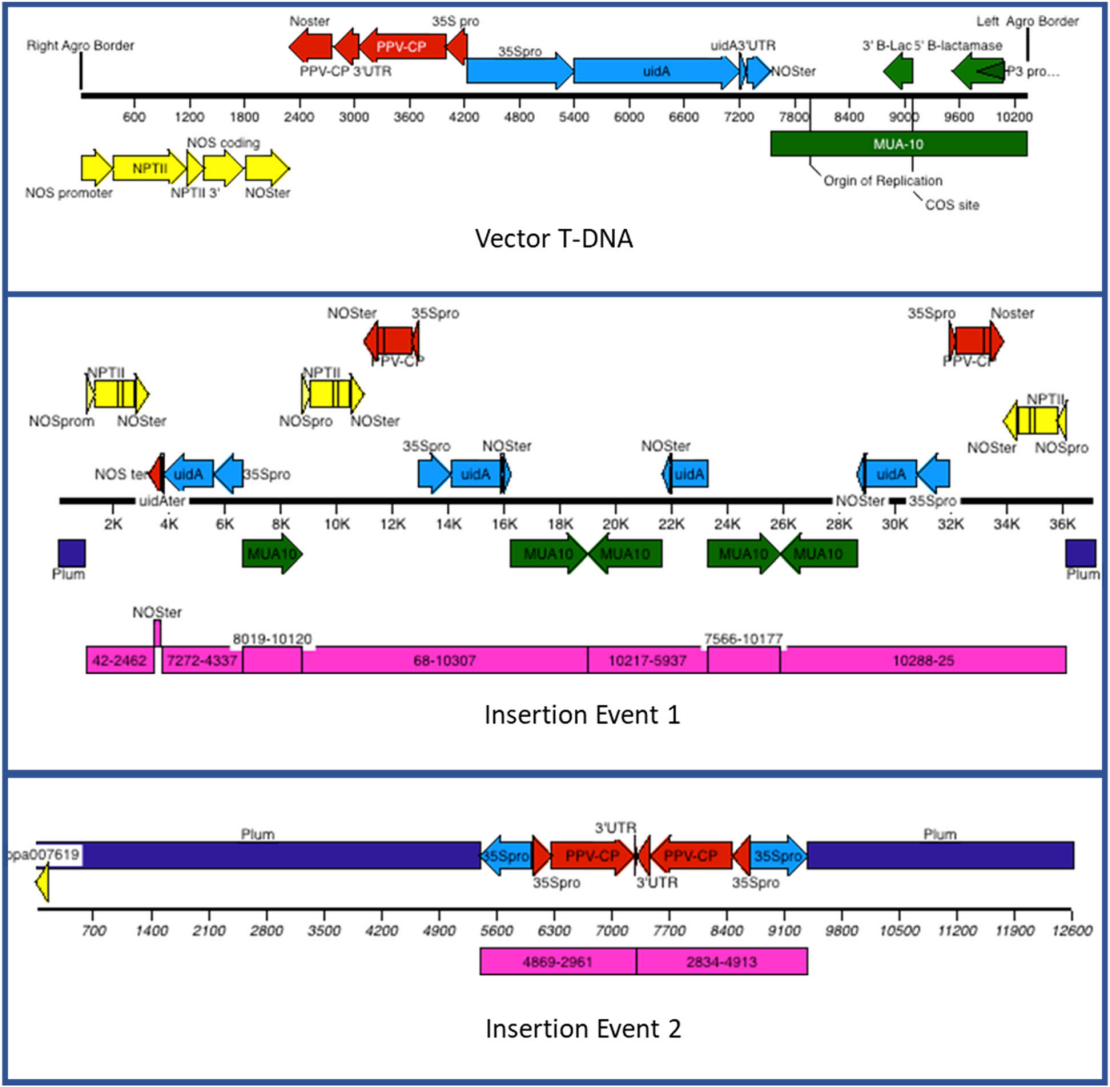
Diagram of the initial transformation T-DNA and the rearrangements resulting at the insertion event 1 and insertion event 2. Each of the transgene regions is color coded with the NPTII gene, promoter, and terminator in yellow, the PPV-CP in red, the UIDA in blue, the MUA-10 in green, and plum in dark blue. The regions in pink represent the fragments of the initial T-DNA specified by the base numbers

Whole genome sequencing was undertaken, not only to confirm the location and arrangement of the transgenes but also to verify the lack of other insertion events undetected by previous DNA blot analyses and PCR detections. Using the Illumina GAII sequencer, 75 and 100 base paired-end sequences, ~607,000 reads were generated after trimming, representing ~52 billion bases of sequence. The sequences were aligned to the transgene T-DNA insert sequence used to transform ‘HoneySweet’ (Supplementary Data Set 1). Junction sequences were detected where the sequence was aligned on only one side of the junction and was not homologous on the other (Fig. S1). In total 11 junction sites were detected (Table S1). Of these, four junctions (junctions #1, 8, 9, and 11) represented inserts flanked by plum DNA. The remaining represented inserts with transgene DNA on one end and non-contiguous transgene DNA on other side. These sites marked duplications, deletions, or other rearrangements during the integration of the T-DNA, a phenomenon known to occur with *Agrobacterium* transformation. A map was constructed based on these junction sites resulting in two separate insertion events, each flanked by plum DNA (Fig. 1).

The first event (insertion 1) consisted of seven copies of the insert with no intervening plum DNA. Two of those copies contained nearly the whole insert and the others contained only partial copies. No T-DNA border sequences were detected in these seven copies. The flanking plum DNA on one side matched that found previously in the BAC sequencing^5^ (see Table S5). On the other side, flanking plum DNA was unique.

The second insertion event (insertion 2) consisted of two copies that each contained the 35S promoter from UIDA and the nearly complete CP gene (Fig. 1) with unequal amounts of the 3′ noncoding region of the CP gene. The copies were arranged toe-to-toe forming a hairpin arrangement with the unequal amounts of 3′ sequence forming an unmatched intervening sequence loop. The flanking plum sequences on either end matched the previously sequenced BAC containing the hairpin arrangement of the CP gene^5^

To confirm the arrangements of the insertion events, primers were designed to the junction sequences and used to verify by PCR that those junction sequences existed in ‘HoneySweet’ (Table S2). Table S1 lists the junction sequences and the PCR reactions that were verified in this manner.

The predicted arrangement of the multiple copies was also confirmed by mapping the ‘HoneySweet’ DNA sequence reads to the predicted insert 1 and 2 sequence. Only sequences that were mapping uniquely were kept, which should represent the junction sequences because of the repetitive nature of the insertion events. All the unique junctions were confirmed (Figs. S2 and S3).

### No additional insertion of foreign DNA

Whole genome sequences were then aligned to the sequence of *Agrobacterium* Ti plasmid DNA as well as *Agrobacterium* genomic DNA and E. coli sequences to determine if there were any unintentional insertions of bacterial vector DNAs. No matches were found indicating that there were no unintentional insertions of bacterial DNA in other parts of the genome.

### Identification of plum scaffolds that have homology to the ‘HoneySweet’ insertion sites

The ~1000 bases of the predicted plum DNA sequence closest to insertion 1 and insertion 2 that were derived from the BAC sequencing and the whole genome sequencing of ‘HoneySweet’ were used to identify scaffolds from the phased plum genome that contained matching sequence. For the multicopy insertion event 1, five scaffolds had significant sequence homology with the ‘HoneySweet’ flanking DNAs (Supplementary Data Set 2). Plum DNA 5’ (991 bases) matched that of Scaffold 2675 with only one base missing, Scaffold 1234 had only an eight base indel, and Scaffolds 1332, 1429, and 1650 also had that eight base indel as well as a few SNPs and additional indels.

The 3′ flanking plum DNA (999 bases) had more divergence. Unlike the 5′ end, this sequence was not derived from BAC sequence but based only on a consensus sequence derived from ‘HoneySweet’ short sequencing reads mapping to the related region in the peach genome. The first 419 bases matched well before diverging amongst the five contigs. In this region there are a handful of SNPs and indels with Scaffolds 2675, 1234, and 1332 sharing most of the SNPs and the ‘HoneySweet’ sequence having some unique SNPs. The sequence following the first 419 bases of ‘HoneySweet’ separated the scaffolds into two groups as the sequence diverged (Supplementary Data Set 2). The first group contained Scaffolds 2675, 1234, and 1332 while the second group consisted of Scaffolds 1429 and 1650. It is hard to place ‘HoneySweet’ reads into either group as reads mapped to either arrangement. Because of the distance from the insertion event, the short reads could not be aligned with the new insertion DNA. The scaffold alignment with the ‘HoneySweet’ short reads predicted that there was a 33-base deletion (or 34 in the case of Scaffold 1429) that was at the site of insertion 1 in ‘HoneySweet’. A sixth scaffold that matched the flanking DNA even with relaxed criterium could not be found for insertion 1.

For the second insertion event, the hairpin, 1000 bases of plum flanking sequence was used for both the 5′ and 3′ that matched the BAC sequence and was confirmed by the whole genome sequence of ‘HoneySweet’. Seven scaffolds were found with significant homologies of which two had two different sites of homology. Of the seven scaffolds, there are four that also contain the flanking sequences for insertion 1, Scaffolds 2675, 1234,1429, and 1650.

A comparison of the sequences with ‘HoneySweet’ for insertion 2 is presented in Supplementary Data Set 3. The site of insertion 2 appears to be in a highly repetitive region with slight variations in sequence. The alignments at the 5′ end were very difficult because of the highly divergent sequences. The homologies improved in the 600 bases closest to insertion site. The alignments suggest grouping of the scaffolds with Scaffold 4101 closest to 1650, and 2675 along with 1234_1 and 1234_2 also grouping with them. Scaffolds 4359 and 1429-1 appear to group and 1429-2 and 6796 appear to be the closest to the ‘HoneySweet’ sequence. But there are variations within the groupings, the most major of which are non-contiguous sequences for Scaffold 4101, 1650 and 1429_2, in which a large insertion of bases appears to be present. For Scaffolds 4101 and 1650 that insertion event is at the same site and identical sequence. The predicted sequence for ‘HoneySweet’ diverges from the other sequences. This could represent a novel arrangement in ‘HoneySweet’ or a miss-assembly of the BAC sequence. There are many matches (~300) of the diverging sequence in the plum genome including 3 in Scaffold 6796 which are located quite close to the predicted insertion site.

### Arrangement of flanking genes

**Fig. 2:**
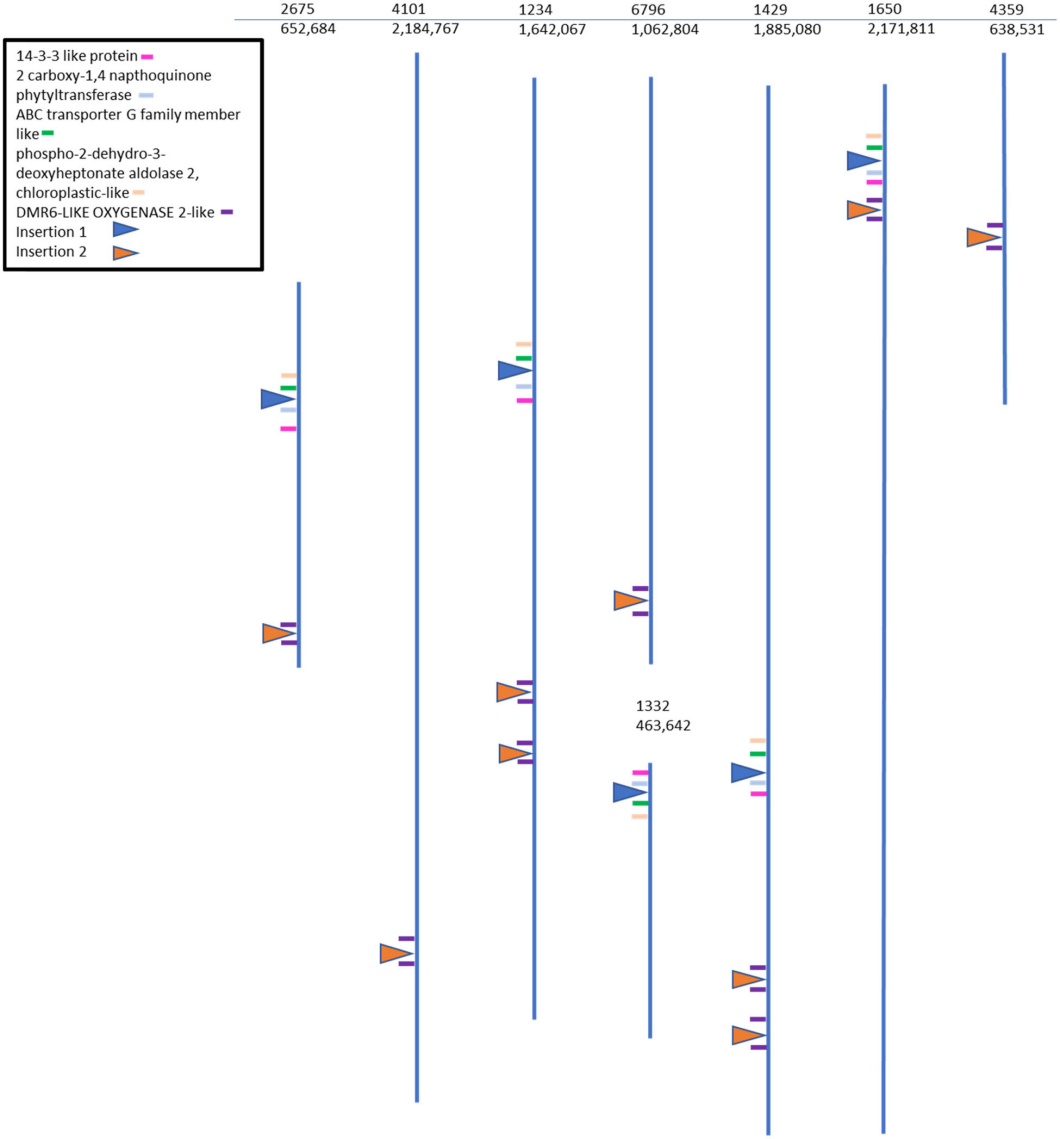
Diagram of the plum scaffolds that contain sites that match the insertion events. Under each scaffold number is the size of the scaffold in bases. The insertion sites are marked with a triangle and the flanking genes with a bar. The key identifies each by color

To further clarify the arrangements as well as possible infringement on gene expression of flanking genes, the closest annotated predicted genes were mapped. Figure 2 presents a schematic and Table S3 presents the actual locations on each scaffold. Insertion 1 is flanked upstream by a 2 carboxy-1,4 naphthoquinone phytyltransferase gene of ~5000 bases. The insertion is located from 1095 to 1976 bases 5′ of the gene in each scaffold. On the downstream side insertion 1 is flanked in three of the scaffolds by a predicted ncRNA of unknown function. In the case of Scaffolds 2675 and 1234 the predicted ncRNA gene is 565 bases away and in Scaffold 1429, it is over 35,000 bases away. All five Scaffolds are then flanked by an ABC transporter G family member like-gene ranging from 15,000 to 38,000 bases away. In none of these five potential sites would the insertion event interrupt a gene.

Insertion 2 was flanked in all cases by *DMR6-LIKE OXYGENASE 2*-like genes, ranging from 6103 to 42,874 away from predicted insertion site on one side and 2337 to 13,351 away on the other side. Again, in none of the scaffolds was the predicted insertion site interrupting a gene.

### Synteny of region with peach genome

**Fig. 3:**
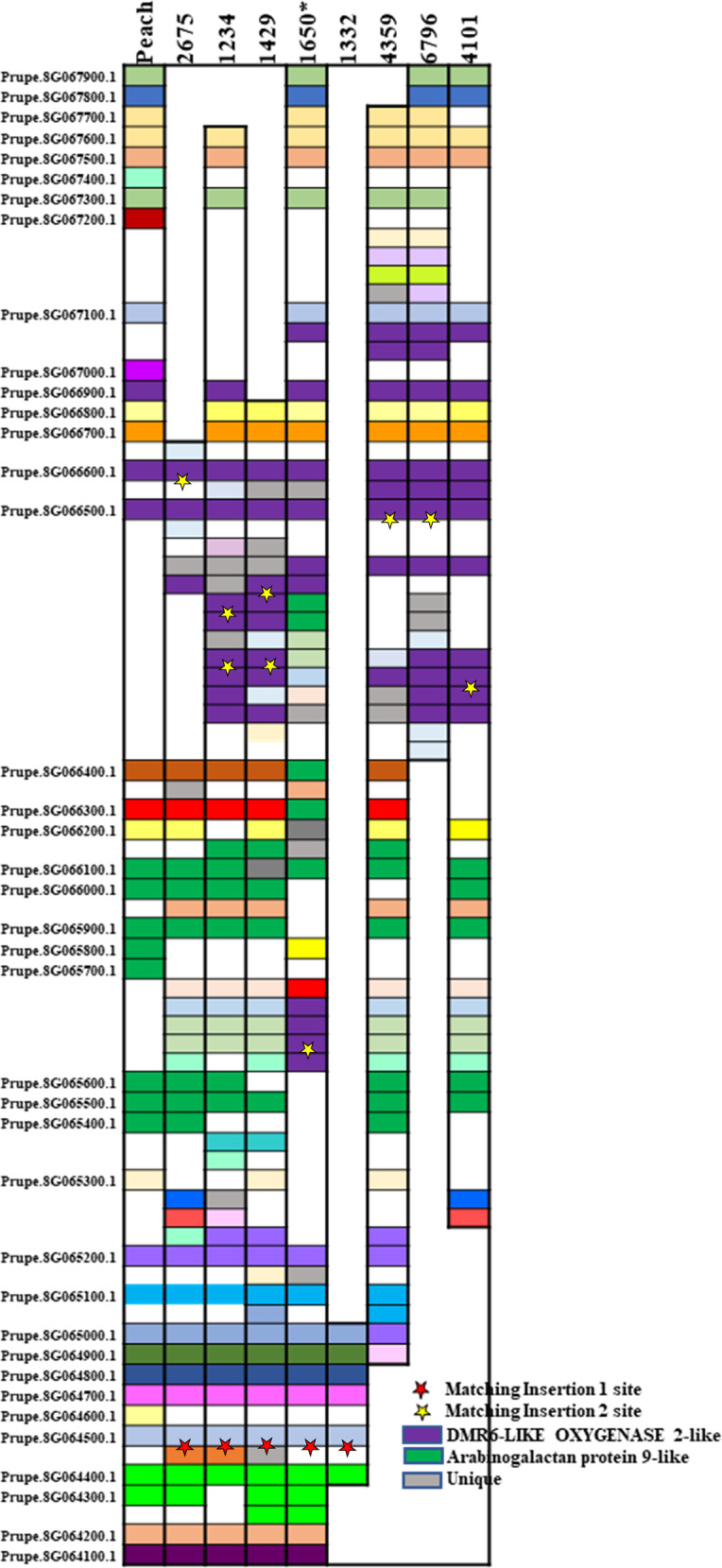
Synteny between peach and the eight scaffolds that have matching sequence to the flanking sequences for insertion 1 and 2. Like color blocks are orthologous genes. The names of all the genes for peach are listed on the side. Light gray blocks for the plum represent unique genes, deep purple blocks represent the repetitive DMR6-like genes and the forest green represent arabinogalactan-9 family genes. Blank space are gene gaps in the sequences. The asterisk for Scaffold 1650 is to indicate that there is an inversion in the middle of the synteny chart. The red stars indicate the location of insertion 1. The yellow stars are indications of where insertion 2 is

The plum scaffolds were compared to the Peach V2.0 genome assembly^9^ to look for regions of synteny, again as further evidence for the relevant plum scaffolds. Previous results suggested a location for related sequences on chromosome 8 of peach, and as expected the synteny was with Pp08 from 9,473,585 to 10,635,400 bases. (Table S4). The eight scaffolds were compared with the region of peach that would cover both insertion sites (9,474,968–9,968,036 bases) with each predicted transcript annotated (Fig. 3). Matching genes have similar colors and non-matching are a similar gray. There are striking homologies at either end of this segment with peach and between all the scaffold sequences that cover these regions. Between these ends, there is considerable variation within the plum scaffolds as well as peach. The first variable is the size of this region. For peach, Scaffolds 2675, 1234, 1429, 1650, 4359, and 4101 the sizes are 493,068, 578,239, 1,218,611, 696,146, 855,117, 618,536, and 784,033, respectively. Scaffold 1332 and 6796 are much shorter at 67,859 and 388,486, respectively. This represents an expansion in the plum genome relative to peach (or a reduction in peach) at this site of up to threefold as well as considerable variation between the plum scaffolds.

There are two sets of repetitive gene sequences in this region, the first is arabinogalactan protein 9-like (*AGP9*) and the second *is DMR6*-Like oxygenase 2-like sequences. There are similar numbers of annotated *AGP9*s in peach (10) and the plum scaffolds (5–8). But for the *DMR6* genes, there are many more in plum at this region (8–10) than in peach (3). One plum scaffold, 2675 also has only 3.

There are small regions where there is significant synteny between the plum scaffolds that are not found in peach, such as the region between Prupe.8G065600.1 and Prupe.8G066000.1, but there is also variation amongst the plum scaffolds, such as between Prupe.8G065200.1 and Prupe.8G065400.1. The last major difference is the inversion found in Scaffold 1650 between Prupe.8G065200.1 and Prupe.8G066500.1.

### Expression of genes flanking matching insertion sites

To verify the lack of influence of the insertion events on expression of flanking genes as well as the intervening space, RNAs were mapped to predicted gene sequences of the newly assembled plum (Figs. S4 and S5). A collection of ‘HoneySweet’ trees from three locations and ‘Stanley’ trees from four locations was used as they represented a variety of environments and management schemes that might affect expression. Quantitatively, there were considerable differences between expression in fruit and leaf tissues, but qualitatively no new sites of transcription were seen between the 16 ‘HoneySweet’ library RNAs and the 20 ‘Stanley’ libraries. To quantify any differences, TPMs (transcripts per million) were calculated and analyzed statistically amongst the different libraries (Table 2). Based initially on the total reads which including those mapping uniquely to each predicted transcript as well as a proportion of those that mapped to multiple predicted transcripts, 10 genes had significant differences in expression in leaf as judged by the *P* value of 0.05 (Table 2). Of these two were significant using the Bonferroni Correction value of 0.05. There were 23 predicted genes that had significant differences of expression in fruit as judged by the *p* value of 0.05 of which only two were significant using the Bonferroni Correction value of 0.05.

**Table 2:** DEGs of flanking genes to predicted insertion sites in all scaffolds

Flanking Gene ID^a^	Insert^b^	Scaffold^c^	HS mean (TPM)^d^	Log^2^ fold change (HS/ST)^e^	Fold change^f^	P-value^g^	FDR p-value^h^	Bonferroni^i^	HS Unique TPMavg (±StDev)^j^	ST Unique TPMavg (±StDev)^k^
Significant DEGs Leaf										
Pd.00g783590	1	1332	0.77	(8.02)	(259.02)	1.16E-07	2.23E-06	0.02	0.01 (0.02)	0.46 (0.32)
Pd.00g783600	1	1332	0.44	(6.06)	(66.68)	5.03E-04	4.26E-03	1	1.07 (3.02)	0.14 (0.23)
Pd.00g1042770	1	1429	3.21	(8.72)	(421.25)	2.44E-08	5.28E-07	0.003	–	0.16 (0.20)
Pd.00g1042800	1	1429	1.57	(5.99)	(63.50)	2.09E-06	3.13E-05	0.29	0.01 (0.04)^l^	1.25 (1.07)
Pd.00g1042440	2	1429	4.58	(2.30)	(4.92)	1.97E-04	1.88E-03	1	0.57 (0.47)	1.28 (0.57)
Pd.00g1042470	2	1429	0.15	(4.13)	(17.55)	1.00E-02	0.07	1	–	–
Pd.00g1042480	2	1429	0.40	5.08	33.91	1.41E-03	1.00E-02	1	–	–
Pd.00g1204440	2	1650	22.09	1.89	3.70	9.15E-05	9.52E-04	1	–	–
Pd.00g357600	2	4101	21.33	1.77	3.42	1.89E-04	1.81E-03	1	–	–
Pd.00g357630	2	4101	0.87	(3.28)	(9.73)	6.86E-05	7.38E-04	1	0.02 (0.03)	0.40 (0.27)
Significant DEGs Fruit										
Pd.00g783610	1	1332	0.27	(2.65)	(6.27)	3.00E-02	0.12	1	0.01 (0.04)	0.23 (0.18)
Pd.00g783620	1	1332	1.17	(2.73)	(6.62)	5.20E-05	5.36E-04	1	0.12 (0.15)	0.85 (0.51)
Pd.00g1042790	1	1429	0.04	3.17	9.03	1.00E-02	5.00E-02	1	0.01 (0.01)	–
Pd.00g1042800	1	1429	0.63	(5.58)	(47.83)	4.18E-04	3.35E-03	1	–	0.50 (0.20)
Pd.00g1042810	1	1429	7.53	1.52	2.86	4.30E-03	2.00E-02	1	1.63 (2.30)	0.64 (0.24)
Pd.00g1042820	1	1429	5.34	(0.97)	(1.96)	4.00E-02	0.15	1	–	–
Pd.00g1204570	1	1650	2.80	1.74	3.35	1.75E-03	1.00E-02	1	1.97 (1.98)	0.68 (0.32)
Pd.00g1204580	1	1650	7.55	3.65	12.54	5.29E-09	1.23E-07	0.001	0.71 (0.50)	0.09 (0.12)
Pd.00g1042430	2	1429	0.82	(6.75)	(107.33)	4.54E-05	4.74E-04	1	–	0.55 (0.62)
Pd.00g1042440	2	1429	5.59	(2.21)	(4.64)	1.39E-03	9.41E-03	1	0.45 (0.56)	1.24 (2.10)
Pd.00g1042470	2	1429	0.09	(3.64)	(12.47)	4.00E-02	0.14	1	–	–
Pd.00g1042480	2	1429	0.33	3.45	10.92	1.75E-03	1.00E-02	1	–	–
Pd.00g1204450	2	1650	1.48	1.90	3.73	1.33E-03	9.11E-03	1	–	–
Pd.00g1204460	2	1650	5.55	2.34	5.07	1.92E-05	2.19E-04	1	–	–
Pd.00g722430	2	2675	0.65	4.85	28.88	1.52E-05	1.78E-04	1	–	–
Pd.00g722750	2	2675	14.65	1.12	2.17	1.00E-02	0.06	1	–	–
Pd.00g357610	2	4101	1.56	2.43	5.39	1.62E-04	1.46E-03	1	-	-
Pd.00g357620	2	4101	5.52	2.50	5.66	7.40E-06	9.32E-05	1	-	0.07 (0.22)
Pd.00g357630	2	4101	0.83	(2.08)	(4.23)	1.36E-03	9.26E-03	1	0.08 (0.06)	0.38 (0.26)
Pd.00g509780	2	4359	0.94	3.53	11.55	2.03E-05	2.31E-04	1	0.14 (0.09)	0.03 (0.04)
Pd.00g509790	2	4359	0.17	2.29	4.89	4.00E-02	0.15	1	0.07 (0.13)	–
Pd.00g864730	2	6796	17.73	(4.80)	(27.94)	1.26E-11	4.37E-10	0.0000	0.22 (0.19)	6.18 (7.77)
Pd.00g864740	2	6796	0.27	3.98	15.83	1.91E-03	1.00E-02	1	–	–

^a^Flanking genes that is significantly different in expression value between ‘HoneySweet’ and ‘Stanley’ based on the average of the individual tree libraries

^b^Insertion event that gene flanks

^c^Scaffold that the differentially expressed gene is located in

^d^The mean is for ‘HoneySweet’ (HS) either 8 libraries for leaf or 8 libraries for fruit tissues. This mean consists of all reads that map whether unique or not

^e^This is the log2 of the ratio between ‘HoneySweet’ (HS) expression and ‘Stanley’ (ST). Negative numbers are in parentheses

^f^the calculated fold change with negative numbers in parentheses

^g^the calculated p value from the variation between the different libraries (8 HS and 10 ST for leaf and for fruit). Only those with a 0.05 or less are presented in this table

^h^False discovery rate calculated

^i^Bonferoni rate

^j^Expression level in ‘HoneySweet’ if only sequenced reads that map to one location are used. This is the mean of 8 individual libraries in Transcripts Per Million with standard error calculated

^k^Expression level in ‘Stanley’ if only sequenced reads that map to one location are used. This is the mean of 10 individual libraries in Transcripts Per Million with standard error calculated

^l^Non-overlapping values

The TPMs were recalculated for these potentially significant genes using only the unique reads, as these were the only transcripts that could definitively be mapped to each allele. On doing this, only three genes had an average TPM with a standard deviation that did not overlap with ‘Stanley’. All three had very low expression from 0 to 1 TPM per library which for most of the libraries meant less than five reads (Table 2). Heat maps were constructed from the TPM values for the unique reads (Tables S5 and S6). These show the variation between each tree sampled is for the most part, greater than the variation by cultivar similar to that seen when sampling fruit composition from these trees^10^.

To confirm that no new genes were formed by the insertion events, all the RNA-seq reads from the 16 ‘HoneySweet’ libraries were mapped to the two predicted insertion events which included ~1000 bases of flanking plum sequence (Fig. S6). Only the expected reads for the coding sequences of *NPTII*, *CP*, *UIDA*, and partial transcripts from the interrupted *BLA* gene in MUA-10 sequence were detected. A few random transcripts were detected but were less than one read per library and hence not significant.

## Discussion

‘HoneySweet’ was chosen as the PPV resistant line to carry through to commercialization because it was the only line out of over 100 independent transformants, with stable resistance to PPV infections in a containment greenhouse. Unfortunately, or fortunately, it had a complex arrangement of transgenes as initially determined by DNA blotting^1,3,4^. Unfortunate, since understanding the complex arrangement would be difficult but fortunate, since the original transgene construct was intended to be an overexpression of the PPV-CP gene, but the complex arrangement resulted in an RNAi mode of resistance before we understood RNAi. The occurrence of rearrangements of insertions is not unusual using *Agrobacterium* transformation: in addition, other insertional effects happen, including but not limited to rearrangements, translocations, deletions, and incorporation of additional DNA^11^. Normally a plant line with a complex arrangement of the introduced transgenes would be discarded at an early stage because of the difficulty predicting the effects on flanking genes as well as potential segregation issues when used in a breeding program. But because of the value of a PPV resistant line of plum, ‘HoneySweet’ was kept and subjected to more extensive analyses of the insertion event. The CABI Invasive Species Compendium states that “Plum pox virus disease (Sharka) is one of the most destructive diseases of stone fruits.” (https://www.cabi.org/isc/datasheet/42203).

The purpose of understanding transgene insertion sites is to verify that no genes have been interrupted or influenced, potentially causing variations in expression that could lead to undesirable phenotypic changes in the transgenic plant. Secondly, that knowledge is used to verify that no new RNAs and potential proteins are generated. And lastly, to verify that no unexpected DNA insertions have happened from carrier DNA such as *Agrobacterium* or *even E. coli* used in the propagation of the vectors. Previous work had suggested that there were two insertions in ‘HoneySweet’ plum, one containing a multicopy, rearranged version of the introduced transgenes (insertion 1) and the second containing an inverted repeat of the CP gene from PPV^4,5^ (insertion 2). The three flanking plum DNA borders from BAC sequence were compared to the peach genome assembly, and indicated that the insertions were in the same region of Pp08 and did not interrupt any genes. To verify this assumption, specific plum genome information was needed. We utilized newer technologies^12–15^, including next generation sequencing of ‘HoneySweet’, and the assembly of a phased genome for the major plum cultivar, ‘Improved French’. ‘Improved French’ was chosen for the genome assembly because it is the major cultivar for the dried plum Industry representing over 65% of the world crop for dried plums. ‘Bluebyrd’, the maternal parent of ‘HoneySweet’, would have provided half of the genome but is only a minor cultivar. The sequence of ‘Improved French’ will have a greater impact on future breeding efforts.

Whole genome sequencing of ‘HoneySweet’ yielded a complete picture of the rearranged insertion event 1 (Fig. 1) that contained seven copies of the transgene insert with various deletions and inversions. These were determined using junction sequences that contained part of the transgene insert joining either plum DNA or an unexpected part of the transgene insert. This also yielded two border sequences, though because of the short reads, the new border sequence could only be extended the length of the short read. Those reads that overlapped the border junction were partially extended with overlapping reads only if there were unique SNPs to verify that the overlapping read was unique from all others, indicating it was part of the same chromosome. Because few of the overlapping reads had unique SNPS, a consensus sequence was used beyond that, which represented the sequence from a majority of the six different chromosomes. The other border sequence was compared to the BAC sequence, which represented only the chromosome that contained the insertion event.

The second insertion event, the hairpin, had been completely covered by a BAC sequence, but was confirmed in the whole genome sequencing by three unique junction sequences as predicted. Reads were then matched to the BAC sequence to verify the plum flanking sequence. When it had been previously aligned in the peach genome, the flanking sequences were found in multiple places and the placement was based on the relative distance to a coding region of one gene found in the BAC flanking sequence, ppa007619 which is equivalent to Prupe.8G066600 in the peach genome assembly 2.0 (ref. ^9^).

To specifically locate the insertion events in the plum genome, a phased plum genome was developed from ‘Improved French’ DNA that would ideally separate out the sequences for the six copies of each chromosome. To gauge the assembly several factors were measured. The first was size (Table 1). The assembly size was 1,399,321,220 bases and because plum is hexaploid, it suggests it should be ~6× the peach genome size (2.274 × 10^8^ haploid_)_ or ~1.3644 × 10^9^. The second was the representation of genes expected to be present as single-copy genes or BUSCO evaluation (Tables 1 and S7). The vast majority of the genes were found in the plum assembly (96%) and interestingly, only 30 of the 1385 were found with six alleles, the majority were found with five alleles (636), with decreasing amounts in 4, 3, and 2 phases and only 76 with a single phase. This reflects not only the hexaploid nature but the high degree of heterozygosity in plum. The last measure of assembly was the number of genes annotated. Again, the expectation would be that it could be up to six times that of the haploid peach (26,873). The plum assembly yielded 130,866 gene models or 4.8× that of the peach. This is consistent with the BUSCO evaluation where only ~2% had six alleles.

With a newly assembled phased plum genome the sequences flanking the insertions of transgenes were used to determine which plum scaffolds could contain the insertions. Scaffolds 1234, 1429, 2675, and 1650 were identified that had sequence homology to the flanking regions of insertion 1 and 2. A fifth scaffold, Scaffold 1332 had homology to only insertion 1 flanking sites, and three additional scaffolds, 4101, 4359, and 6796, had homology to insertion 2 flanking sites. These seven scaffolds (not Scaffold 1332) had significant synteny to a region of the peach Pp08. Looking at the synteny and looking at the mapping, we hypothesize that the nine scaffolds represent six syntenic regions of plum. Scaffolds 1234, 1429, and 1650 each represent one unique phase (one copy of the chromosome). Scaffolds 1332 and 4101 might represent the same phase as one begins where the other ends. Scaffold 2675 and Scaffold 6796 may represent the fifth copy of the chromosome. That hypothesis is based on the decreased number of *DMR6* sites in Scaffold 2675 which ends prior to the region of synteny from Prupe.8G066700.1 which is where Scaffold 6796 begins with multiple *DMR6* sites. This leaves Scaffold 4359 which has only sequences related to insert 2. This scaffold may represent a sixth phase that is homologous at insertion 1 flanking sites with one of the other phases but diverges afterwards. In the assembly of phased sequencing reads, regions of enough homology will not separate into different phases leading to Scaffolds that do not cover the homologous regions.

The fact that the two insertions are in a region of the chromosome that contains two repetitive genes, makes it very difficult to identify the specific scaffold that represents the insertion events. The first insert is near a series of arabinogalactan-9 family genes, but because of the uniqueness of the genes flanking insertion 1, the scaffolds representing that region are quite clear. The second insertion is between repetitive motifs which turn out to be genes from the super family 2-oxoglutarate-dependent dioxygenase or DMR6 like. This has been found to be represented conservatively by more than 100 genes in *Arabidopsis*^16^, most of which map in clusters. In these plum scaffolds the genes are present multiple times with small variations in sequences. The variation in the scaffolds with homology to insertion 2 plum borders may be due to the variability in the number of members of this DMR6-like super family.

The identification with certainty, of the specific scaffold that represents where the insertions are, was not possible. The closest homology with ‘HoneySweet’ is Scaffold 2675, which was very homologous near insertion 1, and Scaffold 4359, which had the most homology at insertion 2. The uncertainty may be related to intra-specific diversity between the two plum cultivars (‘Improved French’ vs. ‘HoneySweet’) and the nucleotidic/structural differences between homologous chromosomes. Without longer sequencing reads for ‘HoneySweet’, this uncertainty remains. Regardless of which scaffold represents the insertion events, none of them interrupt predicted genes. The insertions are in intergenic spaces.

Even though the insertions are not in genes, they might have had an influence of gene expression of flanking genes. When the RNA expression from leaves and fruit from eight ‘HoneySweet’ trees and ten ‘Stanley’ trees was compared, a number of flanking genes from the eight scaffolds had statistically significant differences. But, when looked at using reads that were unique to the flanking genes versus the other related or family members, only four genes showed real differences and for these the expression of three of them did not yield enough reads (>5) to be real and the fourth was also ~1 TPM or again quite low. So our conclusion is that very little change in expression could be attributed more to the presence of the insertions than to differences in environment.

Another aspect of understanding the insertion events of transgenic plants is to understand the breeding potential. One of the problems for plum genetics has been the polyploid aspect where some traits appeared to segregate in a diploid manner, yet the genome is hexaploid. In the case of ‘HoneySweet’ transgenes, *UIDA* and the PPV-*CP*, both appear to segregate close to a 1:1 ratio^17,18^. The UIDA and PPV-CP also co-segregate suggesting that the two insertions are on the same linkage group and close. Most single-copy genes looked at in the BUSCO analysis of the plum genome were found in five different scaffolds implying that there were five variations of those regions. The sixth should/could be a near duplicate of one of those five. In addition, the conclusions of studies looking at the origin of the hexaploid *Prunus domestica* was that it consisted of at least two different *Prunus* genomes^19–21^, an interspecific hybrid of a diploid *P. cerasifera* and a tetraploid *P. spinosa* that itself may have been an interspecific hybrid of *P. cerasifera* and an unknown Eurasian plum species^21–23^. It could be then that two copies come from one species and the other four from a second species. When they assort in meiosis, the four randomly assort and the two segregate so any allele on one of those two segregates as a diploid while the others segregate randomly as a tetraploid. Since it appears that the ‘HoneySweet’ transgenes segregate as a diploid, they should be located on one of the diploid chromosomes. Looking at the diversity at the region of the plum genome where the insertion events are, it could be easily understood that the chromosomes might not all randomly assort because the species divergence did not allow them to pair.

In conclusion, there were two insertion events of the introduced transgenes in ‘HoneySweet’, both resulting in rearrangements and deletions, such that one insertion contained seven modified copies of the transgenes but with at least two complete copies of each gene and the second insertion event resulted in a hairpin arrangement of the PPV-CP transgene driven by a double 35S promoter on each end. These insertion events were each associated with a small deletion of plum DNA, 33 or 39 bases, and were not in any predicted gene. Neither of these insertion events had a dramatic effect on flanking gene expression. Lastly, the insertion events are in a region of the plum genome that has a high level of diversity amongst the different ‘chromosomes’ but without further long-read sequencing of ‘HoneySweet’ in this region, the specific ‘chromosome’ could not be determined.

## Materials and methods

### ‘Improved French’ DNA extraction, sequencing, and assembly

DNA from young (in the end of bud burst) leaves of the ‘Improved French’ was extracted using modified protocol by Kubisiak et al.^24^. Briefly, nuclei were isolated using extraction buffer (0.35 M sorbitol, 10% polyethylene glycol 8000, 0.2% bovine serum albumin, 10 mM Tris-HCl (pH 8.0), 10 mM EDTA, 1 mM spermidine, 1 mM spermine, and 1% β-mercaptoethanol). Pelleted nuclei were washed with organelle wash buffer containing 0.35 M sorbitol, 10 mM Tris-HCl (pH 8.0), 10 mM EDTA, 1 mM spermidine, 1 mM spermine, and 1% β-mercaptoethanol. The DNA from nuclei was extracted by treatment with proteinase K (5 µl per 400 ul) in lysis buffer containing 0.5% N-lauryl sarcosine, 1% CTAB, 0.7 M NaCl at 65 °C for 12 min, phase-separated with equal volume of chloroform: isoamyl alcohol (24:1 vol/vol) extraction and precipitated with 2-isopropanol (1:1 vol/vol). The DNA was washed twice in 70% ethanol, dried at room temperature and resuspended in 0.01 M tris HCl, pH 8.0. Then, the DNA was treated with 3 µl of the Ambion^®^ RNase Cocktail™ (Thermo Fisher Scientific Inc., USA), for 30 min at 37 °C, followed by chloroform: isoamyl alcohol (24:1 vol/vol) extraction and precipitation with two volumes of ethanol. Finally, the DNA was resuspended in 100 µl of 0.01 M Tris-HCl, pH 8.0. The quality and integrity of the DNA were evaluated using Qubit 2 Fluorometer (Thermo Fisher Scientific Inc., USA), a NanoDrop ND-8000 (Thermo Fisher Scientific Inc., USA) followed by electrophoresis on 1% agarose gels.

Sequencing and assembly were performed by NRGene (San Diego, CA) using a combination of Illumina™ technologies including paired-end reads, mate-pair reads of differing sizes, and sequencing and assembly of Chromium 10x libraries (Table S8). The sequencing data were processed and assembled using DeNovoMAGIC™ assembler application version 3.0. The integrity of the assembly was verified using several quality-assurance procedures including the independent BUSCO benchmark (http://busco.ezlab.org/)^7^ which is used to specifically indicate the genic region integrity, ploidy, and zygosity characteristics of the assembled genome. The assembled scaffolds are available at GDR (www.rosaceae.org).

### Genome annotation

A total of 2747 scaffolds (>10 Kb in length) from the genome assembly were annotated using the genome annotation platform GenSAS (www.gensas.org)^25^ and the programs listed below which are integrated into GenSAS. Default settings were used unless otherwise noted. The genome sequence was masked using RepeatMasker (www.repeatmasker.org), other dicots RepBase dataset, and RepeatModeler (www.repeatmasker.org/RepeatModeler). The gene models were predicted using BRAKER2 (https://github.com/Gaius-Augustus/BRAKER) which was trained with a BAM file which contained ‘HoneySweet’ RNA-seq reads (see paragraph below for information on reads) aligned to the genome assembly using HISAT2 (https://ccb.jhu.edu/software/hisat2/index.shtml). tRNA and rRNA were identified using tRNAscan-SE, (http://lowelab.ucsc.edu/tRNAscan-SE)^26^, and RNAmmer, (http://www.cbs.dtu.dk/services/RNAmmer)^27^ respectively. Functional annotation was performed using InterProScan, (http://www.ebi.ac.uk/interpro/search/sequence-search)^28^ Pfam, SignalP, TargetP, and protein alignments with BLAST (SwissProt protein database) and DIAMOND (NCBI, RefSeq, Plant, and *P. persica* proteins from Genbank). BUSCO was run on the predicted proteins, and the genome annotation contains 92.4% of the complete, conserved BUSCOs.

The RNA-seq reads used included a set of ~10 billion raw RNA-seq reads (150 bp paired end) derived from plum vegetative bud and leaf tissues at various stages of development. This RNA-seq set was created using the translatome profiling technique where epitope-tagged ribosomes are immunopurified to enrich for actively translating mRNAs. This technique enriches the mRNA fraction for fully spliced transcripts^29,30^.

Homology of the *Prunus domestica* Genome v1.0 proteins was determined by pairwise sequence comparison using the blastp algorithm against various protein databases. An expectation value cutoff less than 1e^−9^ was used for the NCBI nr (Release 2018-05) and 1e^−6^ for the Arabidopsis proteins (TAIR10), UniProtKB/SwissProt (Release 2018-04), and UniProtKB/TrEMBL (Release 2018-04) databases. The best hit reports are available for download in Excel format at GDR.

### ‘HoneySweet’ genome sequencing

DNA was extracted from ‘HoneySweet’ leaves utilizing a modified nuclei extraction procedure, where 1 g of leaf tissue was ground in liquid nitrogen, transferred to a cold 15 ml Dounce homogenizer containing 7 ml of ice-cold extraction buffer (0.5 M sucrose, 10 mM Trizma Base, 80 mM KCL, 10 mM EDTA, 1 mM spermidine, 1 mM spermine, final pH9.4-9.5 adjusted with NaOH). The material was passaged 4–10 times. The slurry was filtered through two layers of cheese cloth and one layer of miracloth (Calbiochem, San Diego, CA) into a cold centrifuge tube to enrich for nuclei. The nuclei were pelleted by centrifugation at 1800 g at 4 °C for 15 min. The pellet was washed with 5 ml extraction buffer with the addition of 0.15% beta-mercaptoethanol, filtered again through miracloth and re-pelleted. A repeat wash was done. The enriched nuclei were then the starting material for DNA extraction using GNome^®^ DNA kit (BIO101, Vista, CA) where the pellet was resuspended in 1 ml of Suspension Solution and the DNA extracted by manufacturer’s protocol. The resulting DNA was resuspended in 200 µl of water and treated with 2 µl RNaseA(1 mg/ml) for 30 min at 37 °C, followed by phenol/chloroform (1:1 vol/vol) and chloroform extraction^31^. DNA was again precipitated and resuspended in 75 µl water at ~1 mg/ml. The DNA was then sent to DHMRI (David H. Murdock Research Institute, Kanapolis, NC) for sequencing. Initially seven lanes of 75 base paired-end sequences and then four lanes of 100 base-paired reads were processed. Sequences were then trimmed by removing any adapter sequences, low-quality scores and reads less than 60 bases from the 75-base runs and 90 bases from the 100-base runs. These were then analyzed using CLC Genomics Workbench (Qiagen, Germantown, MD), MacVector (MacVector Inc, Apex, NC), and comparisons with peach sequences were done using the *Prunus persica* Genome v1.0 (ref. ^32^) assembly available at GDR, www.rosaceae.org^8^.

### Sequence of transformation vector

The sequence of the original transformation vector T-DNA was recreated through literature searches and available sequence. The sequence is presented in Supplementary Data Set 1. The schematic of the gene arrangement is presented in Fig. 1.

### PCR confirmation of insert sequence

‘HoneySweet’ leaf DNA was extracted using a CTAB protocol^33,34^; starting with ~1 g of fresh leaf and 7.5 ml of extraction buffer. Primer design used MacVector to predict the best sites that flanked the junction sequences as well as other locations in the transgenes (Table S8). Primers were synthesized by IDT (Coralville, IA). PCR used ~50 ng of DNA per 20 µl reaction using Applied Biosystems™ AmpliTaq™ DNA Polymerase with Buffer II (ThermoFisher Scientific) according to manufacturer’s directions. The standard cycle conditions were 5 min at 95 °C, then 30 s at 95 °C, 30 s at 55 °C, and 30 s at 72 °C for 25 cycles followed by 7 min at 72 °C. Variations were added to obtain single fragments of expected sizes, especially for longer fragments, including raising the temperature of annealing from 55 up to 70 °C, changing Mg concentration, adding DMSO, changing cycling program by adding increasing extension times at 72 °C, increasing cycle numbers and using Phusion TAQ polymerase—Phusion (NEB, Ipswitch, MA). The products were run on agarose gels and visualized with Typhoon FLA scanner (GE Healthcare Life Sciences, Marlborough, MA).

### RNA-seq

Leaves and fruit from 8 ‘HoneySweet’ trees and 10 ‘Stanley’ trees located in various sites in Europe and the US were used to look at expression. RNAs were extracted from 20 mg of lyophilized leaf and fruit tissue using the Norgen Plant/Fungi Total RNA Purification Kit (Norgen Biotek Corp., ON) following the manufacturer’s protocol. The RNA was DNased using the TURBO DNA-free kit (Thermo Fisher Scientific, MA) according to the manufacturer’s protocol. The RNA quality and purity were then assessed by electrophoresis on a 1.2% agarose gel and visualized on a Typhoon FLA9500 scanner (GE Healthcare Life Sciences, IL), and by analyzing spectrophotometrically on a NanoDrop^®^ ND-1000 (Thermo Fisher Scientific, MA). Fruit and leaf RNA from each of the individual trees was sent to DHMRI for sequencing (36 libraries), unidirectional reads of 100-base lengths.

### Analyses of plum DNA sequence and RNA sequences

All analyses were done using CLC Genomics Workbench (versions 5.5–12.0). Map to reference was used to map both ‘HoneySweet’ DNA sequences to T-DNA sequence. See analytic pipeline (Fig. S1), peach V1.0 as well as RNA sequences to predicted insertion events. RNA-seq was used to determine transcript rates for flanking genes. Synteny with peach, V2.0. was examined using the Synteny tool present on the GDR website

## Supplementary information

Supplementary Figures 1-6

Supplementary Data Set 1

Supplementary Data Set 2

Supplementary Data Set 3

Supplemental Table 1

Supplemetary Table 2

Supplementary Table 3

Supplementary Table 4

Supplementary Table 5

Supplementary Table 6

Supplementary Table 7

Supplementary Table 8

## Data Availability

RNA-sequencing data are available through Sequence Read Archive (SRA), accession number…. DNA-sequencing data are available through Sequence Read Archive (SRA), accession number…. Plum assembled and annotated genome is available through Genome Database for Rosaceae (GDR) https://www.rosaceae.org/.
