# Supplementary material for: Defining the ‘HoneySweet’ insertion event utilizing NextGen sequencing and a de novo genome assembly of plum (Prunus domestica)
Source: Hortic Res. 2021 Jan 1;8:8. doi: 10.1038/s41438-020-00438-2 (PMC7775438; doi:10.1038/s41438-020-00438-2)
Supplement: Supplementary file 1 — Supplementary Figures 1-6 [file 41438_2020_438_MOESM1_ESM.pdf]

Fig. S1. A pipeline scheme for determining junction sequences. Trimmed equence reads were aligned to the insert (T-DNA) sequence. Junction sequences were identified when only part of the sequence matched the T-DNA. The overhang or non-matching sequence was then determined to be either plum sequence or a non-contiguous part of the T-DNA.

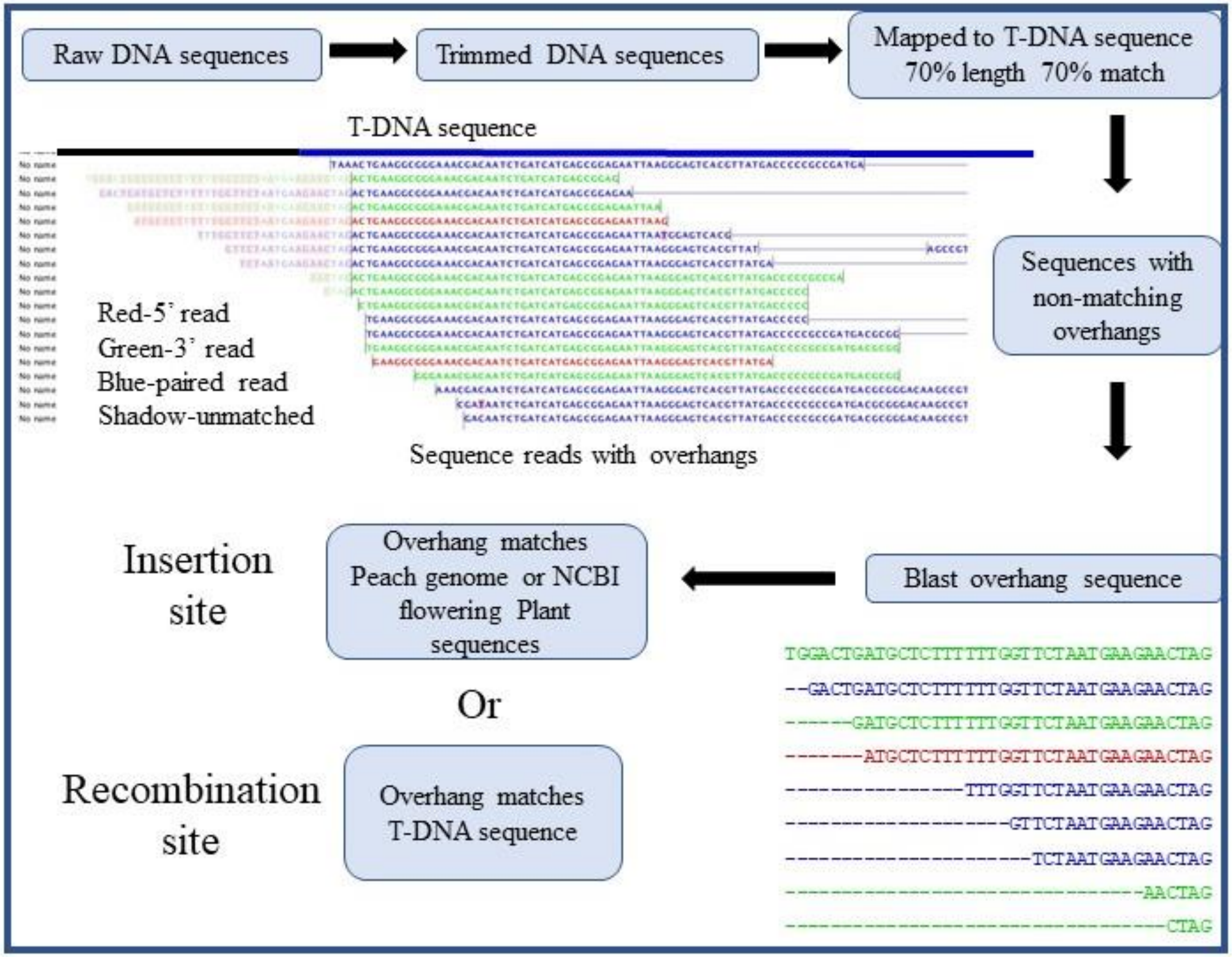

Fig. S2. ‘HoneySweet’ DNA sequence reads mapped to the predicted sequence of the two insertions**.** Only sequences that map to one position are included (unique reads). The condensed map shows that the reads only map to the predicted junction sites in the arrangement. Insertion 1 has seven fragments flanked by the junction sites and insertion 2 has two fragments flanked by junction sites. Blue lines represent paired reads with green and red being only one or the other strand.

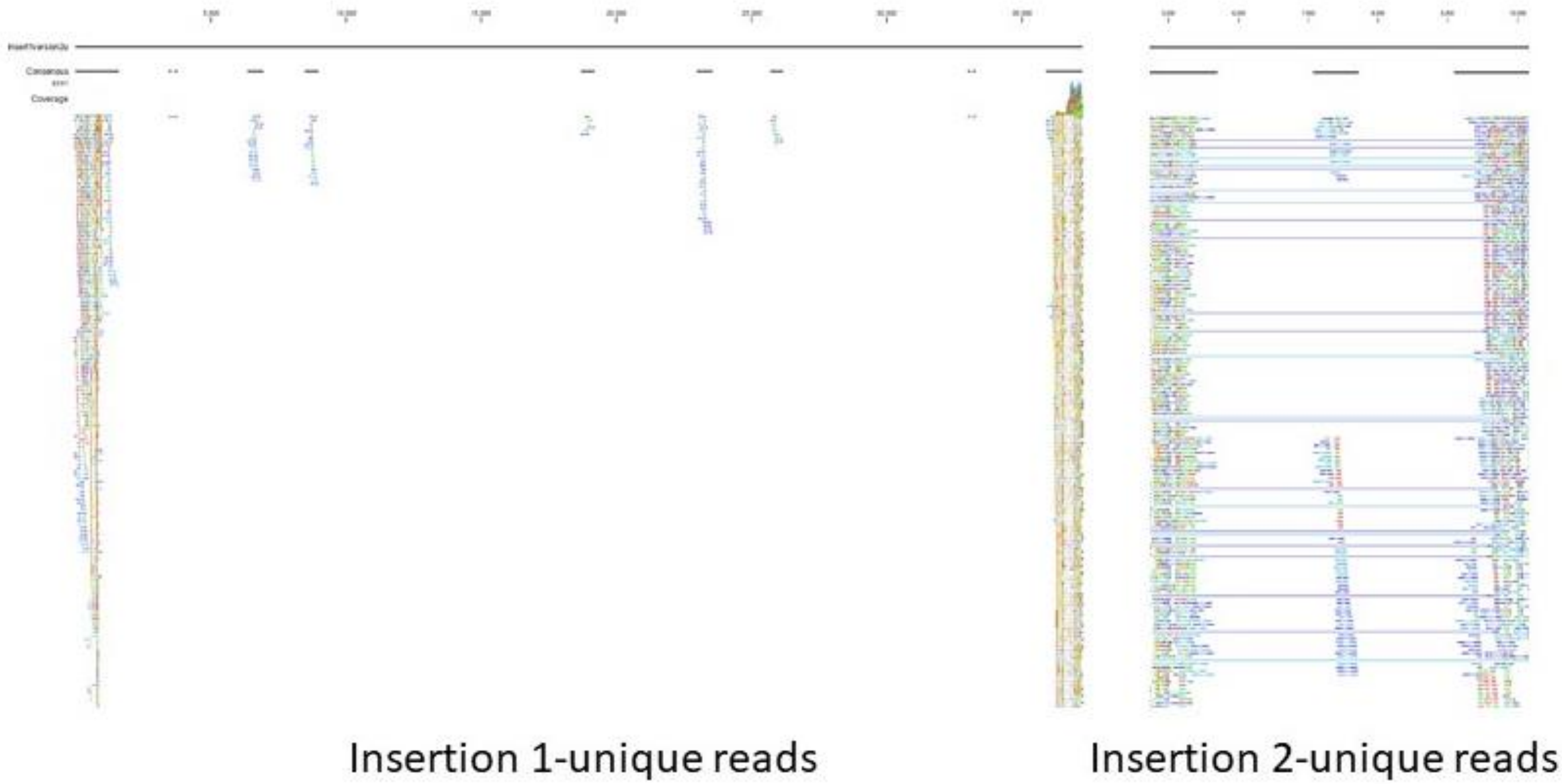

Fig. S3. A diagram of the fragments and rearrangements of the inserted transgene DNA and verification through PCR. Each color represents a different part of the insert as defined below, purple is plum DNA, yellow belongs to the NPTII cassette, blue to the UIDA cassette, green to pBR322 cassette and red to the PPV-CP cassette. Where ever there is a star, it means that junction was confirmed by PCR and exists in ‘HoneySweet’. The list of fragments is below each insertion map with sizes of each copy of insertion and nucleotide positions based on the insertion T-DNA (Supplementary Data Set 1).Carrots indicate the direction of the copy and the text describes the endpoint sequences.

Insertion One

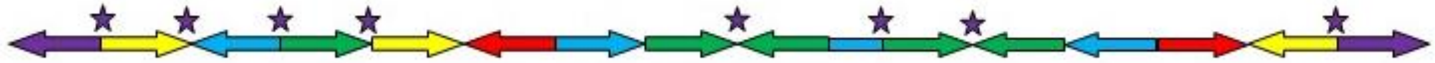

	Base # Description of components	Bases
1.	42-2472 >>>NosProm-NPT-NosTerm	2,430
2.	7272-4337<<<uidA3’-uidA-35SProm	2,935
3.	8019-10120>>>pBR322-B-lactamase-P3promoter	2,101
4.	63-10,000>>>NosProm-NPT-CP-uid-B-Lactamase	10,137
5.	10,000-7566or5937<<<B-lactamase-pBR322 or uidA(no promoter)	4,263
6.	7566 of 5937-10,000 >>>pBR322 or uidA(no Promoter)-B-lactamase	2,634
7.	10,000-25<<<B-lactamase-uidA-CP-NPT	10,175
		34,675

Insertion Two

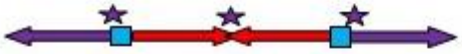

1.	4869-2834<<<35Sprom-CP-3’	2,035
2.	2962-4917>>>CP-35Sprom	1,955
		3,990

★ Verified junction utilizing PCR
Purple—Plum DNA
Yellow—*NPT* gene (Kanamycin resistance)
Blue—*UidA* gene (GUS)
Red—PPV-CP gene (coat protein from Plum Pox Virus strain D)

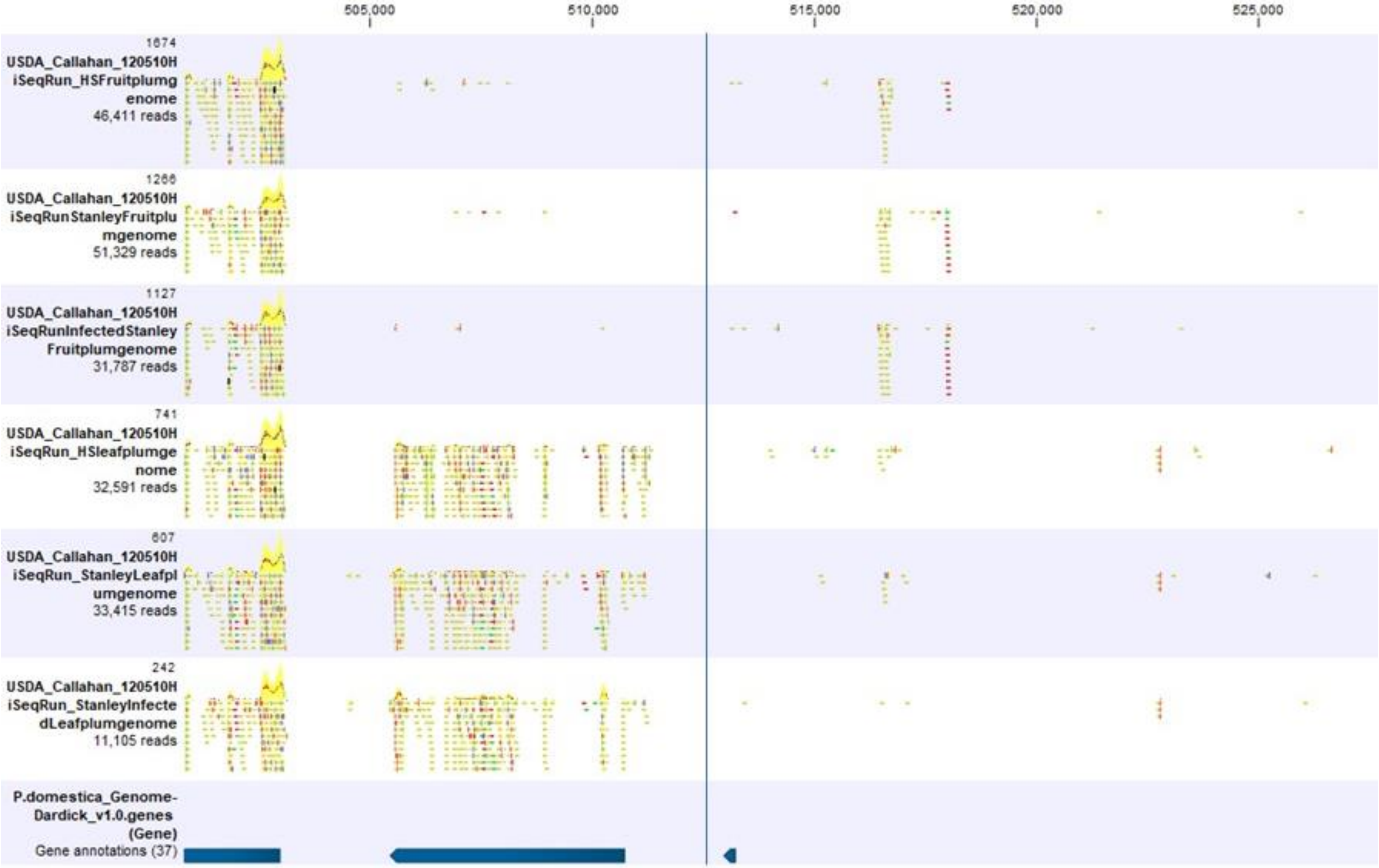

Fig. S4. RNA expression of genes flanking the site of insertion 1 in Scaffold 2675. No new transcripts were detected and no differences in expression patterns between 'HoneySweet', 'Stanley' and 'Stanley' infected with PPV. Each line represents a sequencing read with yellow representing a sequence that maps to more than one location, red and green are unique but either the positive or negative strand of DNA. The vertical line is the site of the insertion and the horizontal arrows on the bottom are predicted exons and represent the only predicted gene in the region presented.

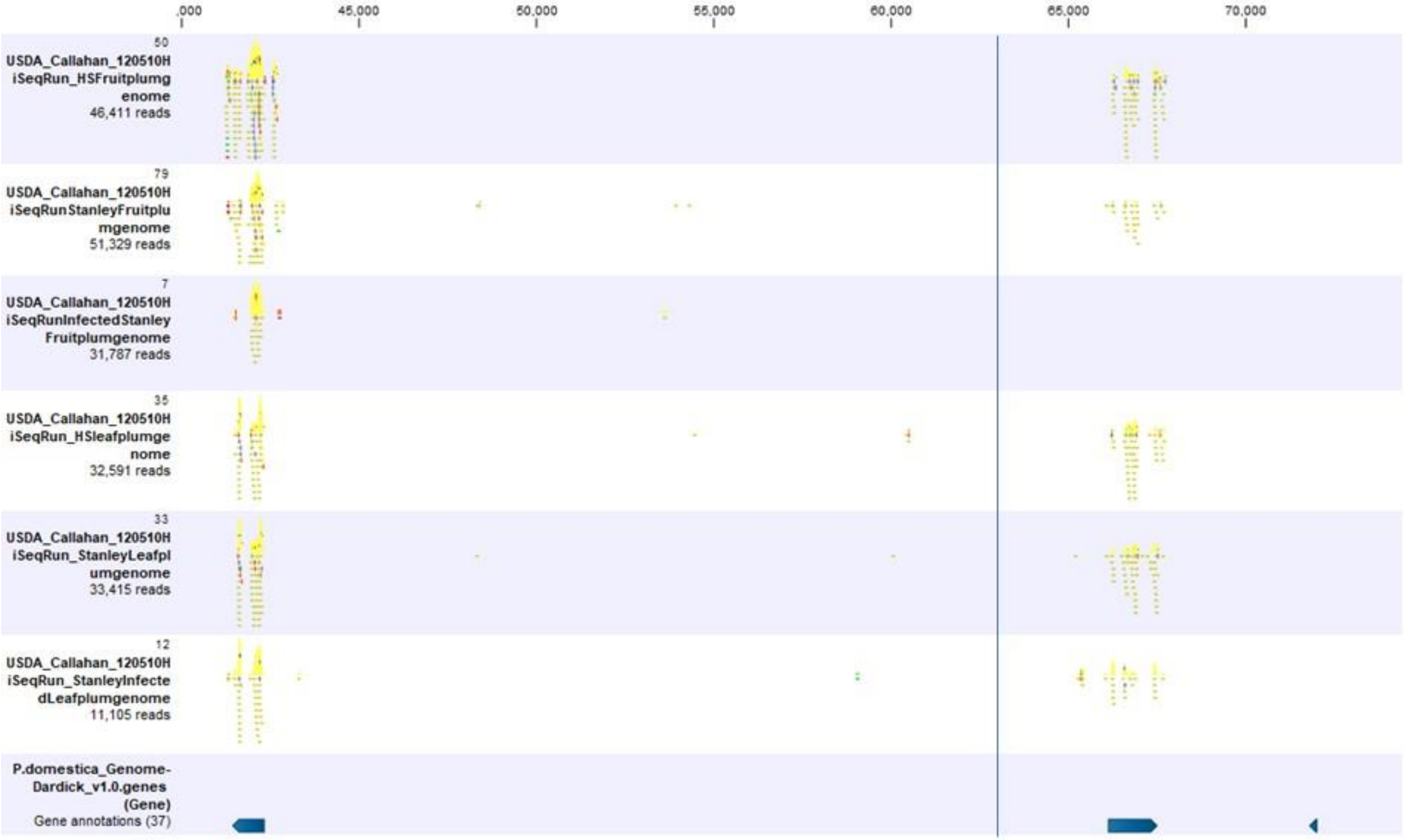

Fig. S5. RNA expression of genes flanking the site of insertion 2 in Scaffold 2675. No new transcripts were detected and no differences in expression patterns between 'HoneySweet', 'Stanley' and 'Stanley' infected with PPV. Each line represents a sequencing read with yellow representing a sequence that maps to more than one location, red and green are unique but either the positive or negative strand of DNA. The vertical line is the site of the insertion and the horizontal arrows on the bottom are predicted exons in the region presented.

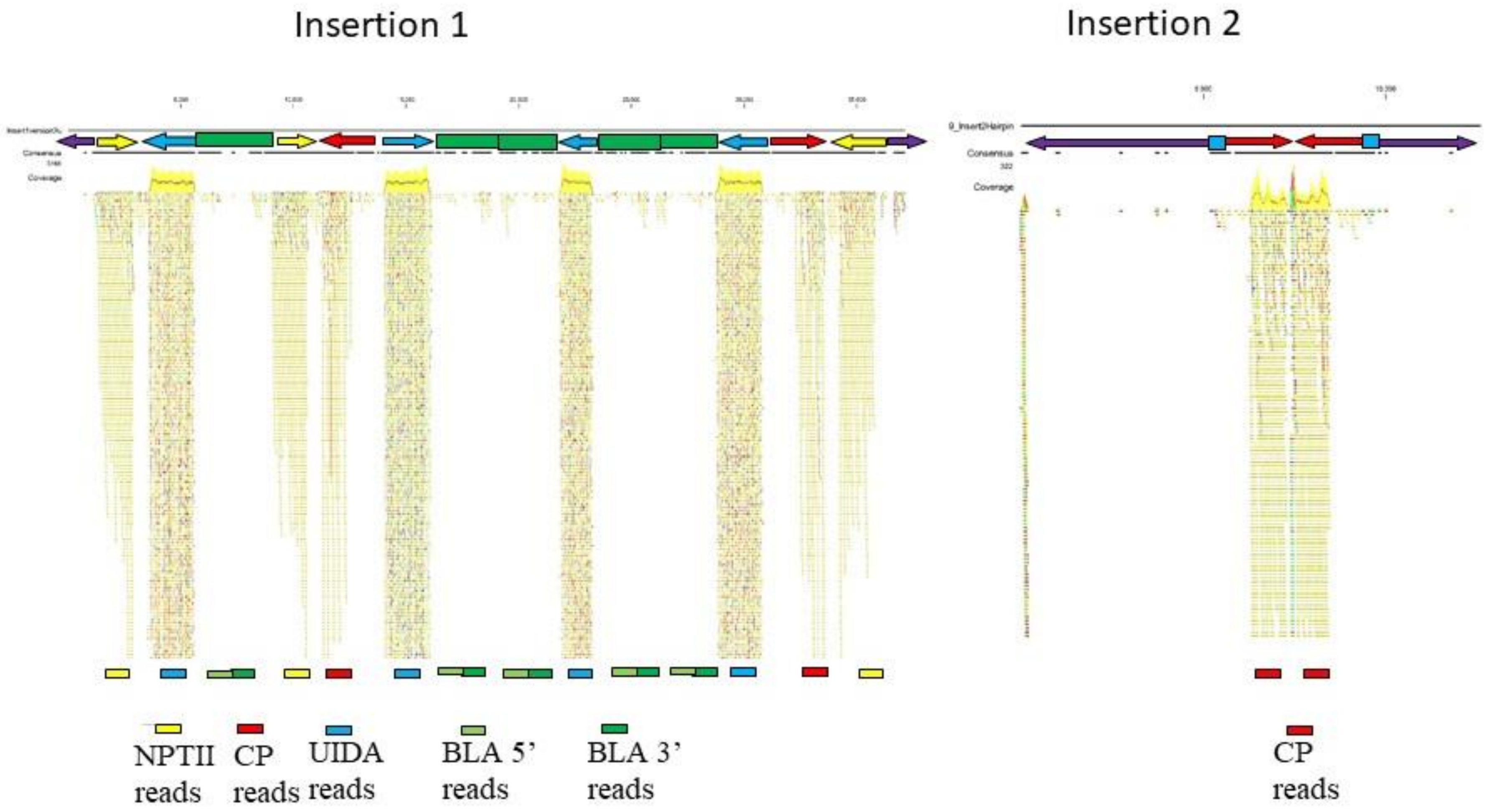

Fig. S6. RNAseq reads mapped to the predicted sequence of the two insertions**.** Again, there are no significant reads in the plum regions and no reads outside of the predicted genes suggesting that no new transcripts arise from the rearranged insert or from the plum-insert junctions. Correspondingly, no new proteins are produced by the transgene insert events. The color scheme for the insertion is the same as in Figure 1 and the color of the sequencing reads-yellow for non-unique mapping, and red and green for unique but to one strand only. The different gene reads are also identified at the bottom of the figure where highly expressed genes line UIDA have reads.
