# Supplementary material for: Defining the ‘HoneySweet’ insertion event utilizing NextGen sequencing and a de novo genome assembly of plum (Prunus domestica)
Source: Hortic Res. 2021 Jan 1;8:8. doi: 10.1038/s41438-020-00438-2 (PMC7775438; doi:10.1038/s41438-020-00438-2)
Supplement: Supplementary file 2 — Supplementary Data Set 1 [file 41438_2020_438_MOESM2_ESM.pdf]

Supplementary Data Set 1. Predicted sequence and annotation of the T-DNA from the transformation vector. Sequence is only predicted because it is assembled from a combination of literature searches and sequence. It has not been sequenced.

```
LOCUS       'HoneySweet' T-DNA from VECTOR 10361 BP DS-DNA

FEATURES             Location/Qualifiers
     misc_feature    1..25
                     /note="Agro Border"
     promoter        26..417
                     /note="NOS promoter"
     CDS             418..1170
                     /note="NPTII"
     3'UTR           1174..1359
                     /note="NPTII 3'"
     3'UTR           1360..1787
                     /note="NOS coding"
     3'UTR           1811..2287
                     /note="NOS term(NPT)"
     3'UTR           2288..2743
                     /note="Nos Term (CP)"
     3'UTR           complement(2743..3038)
                     /note="PPV-CP 3'UTR"
     3'UTR           7277..7543
                     /note="NOS Term"
     misc_feature    7544..10336
                     /note="pBR322 "
     misc_feature    10337..10357
                     /note="Agro Border"
     misc_feature    7979..7985
                     /note="Orgin of Replication"
     CDS             complement(8774..9084)
                     /note="3' end of coding Beta-Lactamase"
     misc_feature    9085..9092
                     /note="COS site"
     CDS             complement(9528..10073)
                     /note="5' end coding Beta-lactamase"
     promoter        complement(10110..10118)
                     /note="Procaryote P3 promoter"
     CDS             complement(3042..3992)
                     /note="PPV-CP"
     promoter        complement(3993..4222)
                     /note="35S prom-CP"
     promoter        4222..5394
                     /note="35SpromGUS"
     CDS             5395..7203
                     /note="uidA"
     3'UTR           7207..7276
                     /note="uidA 3'UTR"
BASE COUNT      2721 A      2507 C      2503 G      2626 T         4 OTHER
ORIGIN      -
        1 GTTTACCCGC CAATATATCC TGTCAAACAC TGATAGTTTA AACTGAAGGC GGGAAACGAC
       61 AATCTGATCA TGAGCGGAGA ATTAAGGGAG TCACGTTATG ACCCCCGCCG ATGACGCGGG
```

121 ACAAGCCGTT TTACGTTTGG AACTGACAGA ACCGCAACGT TGAAGGAGCC ACTCAGCCGC
181 GGGTTTCTGG AGTTTAATGA GCTAAGCACA TACGTCAGAA ACCATTATTG CGCGTTCAAA
241 AGTCGCCTAA GGTCACTATC AGCTAGCAAA TATTTCTTGT CAAAAATGCT CCACTGACGT
301 ACCATAAATT CCCCTCGGTA TCCAATTAGA GTCTCATATT CACTCTCAAT CCAAATAATC
361 TGCAATGGCA ATTACCTTAT CCGCAACTTC TTTACCTATT TCCGCCCGGA TCCGGGCAGG
421 TTCTCCGGCC GCTTGGGTGG AGAGGCTATT CGGCTATGAC TGGGCACAAC AGACAATCGG
481 CTGCTCTGAT GCCGCCGTGT TCCGGCTGTC AGCGCAGGGG CGCCCGGTTC TTTTTGTCAA
541 GACCGACCTG TCCGGTGCCC TGAATGAACT GCAGGACGAG GCAGCGCGGC TATCGTGGCT
601 GGCCACGACG GGCGTTCCTT GCGCAGCTGT GCTCGACGTT GTCACTGAAG CGGGAAGGGA
661 CTGGCTGCTA TTGGGCGAAG TGCCGGGGCA GGATCTCCTG TCATCTCACC TTGCTCCTGC
721 CGAGAAAGTA TCCATCATGG CTGATGCAAT GCGGCGGCTG CATACGCTTG ATCCGGCTAC
781 CTGCCCATTC GACCACCAAG CGAAACATCG CATCGAGCGA GCACGTACTC GGATGGAAGC
841 CGGTCTTGTC GATCAGGATG ATCTGGACGA AGAGCATCAG GGGCTCGCGC CAGCCGAACT
901 GTTCGCCAGG CTCAAGGCGC GCATGCCCGA CGGCGAGGAT CTCGTCGTGA CCCATGGCGA
961 TGCCTGCTTG CCGAATATCA TGGTGGAAAA TGGCCGCTTT TCTGGATTCA TCGACTGTGG
1021 CCGGCTGGGT GTGGCGGACC GCTATCAGGA CATAGCGTTG GCTACCCGTG ATATTGCTGA
1081 AGAGCTTGGC GGCGAATGGG CTGACCGCTT CCTCGTGCTT TACGGTATCG CCGCTCCCGA
1141 TTCGCAGCGC ATCGCCTTCT ATCGCCTTCT TGACGAGTTC TTCTGAGCGG GACTCTGGGG
1201 TTCGAAATGA CCGACCAAGC GACGCCCAAC CTGCCATCAC GAGATTTCGA TTCCACCGCC
1261 GCCTTCTATG AAAGGTTGGG CTTCGGAATC GTTTTCCGGG ACGCCGGCTG GATGATCCTC
1321 CAGCGCGGGG ATCTCATGCT GGAGTTCTTC GCCCACCCCG ATCCAACACT TACGTTTGCA
1381 ACGTCCAAGA GCAAATAGAC CACGAACGCC GGAAGGTTGC CGCAGCGTGT GGATTGCGTC
1441 TCAATTCTCT CTTGCAGGAA TGCAATGATG AATATGATAC TGACTATGAA ACTTTGAGGG
1501 AATACTGCCT AGCACCGTCA CCTCATAACG TGCATCATGC ATGCCCTGAC AACATGGAAC
1561 ATCGCTATTT TTCTGAAGAA TTATGCTCGT TGGAGGATGT CGCGGCAATT GCAGCTATTG
1621 CCAAAATCGA AATACCCCTC ACGCATGCAT TCATCAATAT TATTCATGCG GGGAAAGGCA
1681 AGATTAATCC AACTGGCAAA TCATCCAGCG TGATTGGTAA CTTCAGTTCC AGCGACTTGA
1741 TTCGTTTTGG TGCTACCCAC GTTTTCAATA AGGACGAGAT GGTGGAGTAA AGAAGGAGTG
1801 CGTCGAAGCA GATCGTTCAA ACATTTGGCA ATAAAGTTTC TTAAGATTGA ATCCTGTTGC
1861 CGGTCTTGCG ATGATTATCA TATAATTTCT GTTGAATTAC GTTAAGCATG TAATAATTAA
1921 CATGTAATGC ATGACGTTAT TTATGAGATG GGTTTTTATG ATTAGAGTCC CGCAATTATA
1981 CATTTAATAC GCGATAGAAA ACAAAATATA GCGCGCAAAC TAGGATAAAT TATCGCGCGC
2041 GGTGTCATCT ATGTTACTAG ATCGATCAAA CTTCGGTACT GTGTAATGAC GATGAGCAAT
2101 CGAGAGGCTG ACTAACAAAA GGTATGCCCA AAAACAACCT CTCCAAACTG TTTCGAATTG
2161 GAAGTTTCTG CTCATGCCGA CAGGCATAAC TTAGATATTC GCGGGCTATT CCCACTAATT
2221 CGTCCTGCTG GTTTGCGCCA AGATAAATCA GTGCATCTCC TTACAAGTTC CTCTGTCTTG
2281 TGAAATGAAC TGCTGACTGC CCCCCAAGAA AGCCTCCTCA TCTCCCAGTT GGCGGCGGCT
2341 GATACACCAT CGAAAACCCA CGTCCGAACA CTTGATACAT GTGCCTGAGA AATAGGCCTA
2401 CGTCCAAGAG CAAGTCCTTT CTGTGCTCGT CGGAAATTCC TCTCCTGTCA GACGGTCGTG
2461 CGCATGTCTT GCGTTGATGA AGCTTCTAGA GATCTAGTAA CATAGATGAC ACCGCGCGCG
2521 ATAATTTATC CTAGTTTGCG CGCTATATTT TGTTTTCTAT CGCGTATTAA ATGTATAATT
2581 GCGGGACTCT AATCATAAAA ACCCATCTCA TAAATAACGT CATGCATTAC ATGTTAATTA
2641 TTACATGCTT AACGTAATTC AACAGAAATT ATATGATAAT CATCGCAAGA CCGGCAACAG
2701 GATTCAATCT TAAGAAACTT TATTGCCAAA TGTTTGAACG ATCTGCAGGT CGACGGATCC
2761 CCTTTTTTTT TTTTTGTCTC TTGCACAAGA ACTATAACCC GAATGGAGTG AAGCACTCGC
2821 TACATCAGAT ACAAGGGCCT GTGTTCGACA ATAACAGACT AGAACATATG GAGGTAAAAC
2881 CTCACTGAAT GTACTGCTAT TAAAGCGGAG AAAAGGATGC TAACAGGAAT CTAAAAACAA
2941 CTGGATGATT AGACTCTCAC CCAGGTAGAG TTTATGATAG ATACCGAGAC CACTACACTC
3001 CCCTCACACC GAGGAGGTTG TGCATGTTGC GATTAACATC ACCAGCGGTG TGTCTCTCTG
3061 TGTCCTCTTC TTGTGTTCCG ACGTTTCCAT CCAAGCCAAA TAAACGATTT TGAACATTTC
3121 TCAATGCTGC TGCCTTCATC TGGATATGAG CTTCACGTGC CCGTACGGGT GTCGTTGAAG
3181 TCATTTCGTA AAAATCAAAG GCATATCTGG CGAGGCTGTA GTCTGTCAGG TTGCGCTGAA
3241 TTCCATACCT TGGCATGTAT GCTTTTTCAT AATTTCGTTT TTCAATATAC GCTTCAGCCA
3301 CGTTACTGAA ATGTGCCATA ATTTGTCTAA AAGTGGGTTT CGCATGATCC AACAATGGCT
3361 TTATTGGATA CTCCACTTGT GTTTCCCCAT CCATCATCAC CCACATTCCA TTGATATTCG
3421 GGGATGTTCC ATTCTCTATG CACCAAACCA TAAGACCATT TAAAATGATG CTCATTTCAT
3481 CGTCCGTGAC ATCATARTCT CGCTTAACTC CTTCATACCA AGTTTGGAAA CAAGACTGCG

3541 GAGCTCTCGT GTTTGACAAG TCAACCTGTG CAGGACTATA ATGTGCCAAA TGGTTCAAGT
3601 TCATAATAGC CTTTCCCTTC ACCTTTGGCA GAGATAGTTT CGAAGTCATT GCCTTCAAAC
3661 GTGGCACTGT AAAAGTTCCA ATTGATCCTG CATCGACGTC CCTGTCTCTG TTTGTGTTGA
3721 CTAGCGCGTT TGAGTTGCTA GGTGATGCAT CCTCATTACC ATATGTTCCA AARGTTTGCA
3781 GTTGAGGTCC TGRCACCTGT GAAACTGGTT TTGTTGCTGG TTGAGTTGTT GCTGGCGTGA
3841 AAATGGGGTT GAGCATTGGC GCCGTAGTCC GGGGTGCAGG CTGTATGACT GGAGGTGGTT
3901 GAAGTATTGG GCTAGTTGCT GCCGGTGYAG TAACTACAAT CGGCTTGCCT GCATCAACTT
3961 CCTCCTCGTC TTCTCTTTCG TCAGCTTGGC ATGCCATATT TAAAACGGAT CCGTCGACCT
4021 GCAGGTCGTC CTCTCCAAAT GAAATGAACT TCCTTATATA GAGGAAGGGT CTTGCGAAGG
4081 ATAGTGGGAT TGTGCGTCAT CCCTTACGTC AGTGGAGATA TCACATCAAT CCACTTGCTT
4141 TGAAGACGTG GTTGGAACGT CTTCTTTTTC CACGATGCTC CTCGTGGGTG GGGGTCCATC
4201 TTTGGGACCA CTGTCGGCAG AGGCATCTTG AACGATAGCC TTTCCTTTAT CGCAATGATG
4261 GCATTTGTAG GTGCCACCTT CCTTTTCTAC TGTCCTTTTG ATGAAGTGAC AGATAGCTGG
4321 GCAATGGAAT CCGAGGAGGT TTCCCGATAT TACCCTTTGT TGAAAAGTCT CAATAGCCCT
4381 TTGGTCTTCT GAGACTGTAT CTTTGATATT CTTGGAGTAG ACGAGAGTGT CGTGCTCCAC
4441 CATGTTGACG GATCTCTAGA AGCTTCTAGA GCTCGTTAAC GGTACCATCG ATAGATCCCC
4501 AGCTTGCATG CCTGCAGGTC CCCAGATTAG CCTTTTCAAT TTCAGAAAGA ATGCTAACCC
4561 ACAGATGGTT AGAGAGGCTT ACGCAGCAGG TCTCATCAAG ACGATCTACC CGAGCAATAA
4621 TCTCCAGGAA ATCAAATACC TTCCCAAGAA GGTTAAAGAT GCAGTCAAAA GATTCAGGAC
4681 TAACTGCATC AAGAACACAG AGAAAGATAT ATTTCTCAAG ATCAGAAGTA CTATTCCAGT
4741 ATGGACGATT CAAGGCTTGC TTCACAAACC AAGGCAAGTA ATAGAGATTG GAGTCTCTAA
4801 AAAGGTAGTT CCCACTGAAT CAAAGGCCAT GGAGTCAAAG ATTCAAATAG AGGACCTAAC
4861 AGAACTCGCC GTAAAGACTG GCGAACAGTT CATACAGAGT CTCTTACGAC TCAATGACAA
4921 GAAGAAAATC TTCGTCAACA TGGTGGAGCA CGACACACTT GTCTACTCCA AAAATATCAA
4981 AGATACAGTC TCAGAAGACC AAAGGGCAAT TGAGACTTTT CAACAAAGGG TAATATCCGG
5041 AAACCTCCTC GGATTCCATT GCCCAGCTAT CTGTCACTTT ATTGTGAAGA TAGTGGAAAA
5101 GGAAGGTGGC TCCTACAAAT GCCATCATTG CGATAAAGGA AAGGCCATCG TTGAAGATGC
5161 CTCTGCCGAC AGTGGTCCCA AAGATGGACC CCCACCCACG AGGAGCATCG TGGAAAAAGA
5221 AGACGTTCCA ACCACGTCTT CAAAGCAAGT GGATTGATGT GATATCTCCA CTGACGTAAG
5281 GGATGACGCA CAATCCCACT ATCCTTCGCA AGACCCTTCC TCTATATAAG GAAGTTCATT
5341 TCATTTGGAG AGAACACGGG GGACTCTAGA GGATCCCCGG GTGGTCAGTC CCTTATGTTA
5401 CGTCCTGTAG AAACCCCAAC CCGTGAAATC AAAAAACTCG ACGGCCTGTG GGCATTCAGT
5461 CTGGATCGCG AAAACTGTGG AATTGATCAG CGTTGGTGGG AAAGCGCGTT ACAAGAAAGC
5521 CGGGCAATTG CTGTGCCAGG CAGTTTTAAC GATCAGTTCG CCGATGCAGA TATTCGTAAT
5581 TATGCGGGCA ACGTCTGGTA TCAGCGCGAA GTCTTTATAC CGAAAGGTTG GGCAGGCCAG
5641 CGTATCGTGC TGCGTTTCGA TGCGGTCACT CATTACGGCA AAGTGTGGGT CAATAATCAG
5701 GAAGTGATGG AGCATCAGGG CGGCTATACG CCATTTGAAG CCGATGTCAC GCCGTATGTT
5761 ATTGCCGGGA AAAGTGTACG TATCACCGTT TGTGTGAACA ACGAACTGAA CTGGCAGACT
5821 ATCCCGCCGG GAATGGTGAT TACCGACGAA AACGGCAAGA AAAAGCAGTC TTACTTCCAT
5881 GATTTCTTTA ACTATGCCGG AATCCATCGC AGCGTAATGC TCTACACCAC GCCGAACACC
5941 TGGGTGGACG ATATCACCGT GGTGACGCAT GTCGCGCAAG ACTGTAACCA CGCGTCTGTT
6001 GACTGGCAGG TGGTGGCCAA TGGTGATGTC AGCGTTGAAC TGCGTGATGC GGATCAACAG
6061 GTGGTTGCAA CTGGACAAGG CACTAGCGGG ACTTTGCAAG TGGTGAATCC GCACCTCTGG
6121 CAACCGGGTG AAGGTTATCT CTATGAACTG TGCGTCACAG CCAAAAGCCA GACAGAGTGT
6181 GATATCTACC CGCTTCGCGT CGGCATCCGG TCAGTGGCAG TGAAGGGCGA ACAGTTCCTG
6241 ATTAACCACA AACCGTTCTA CTTTACTGGC TTTGGTCGTC ATGAAGATGC GGACTTGCGT
6301 GGCAAAGGAT TCGATAACGT GCTGATGGTG CACGACCACG CATTAATGGA CTGGATTGGG
6361 GCCAACTCCT ACCGTACCTC GCATTACCCT TACGCTGAAG AGATGCTCGA CTGGGCAGAT
6421 GAACATGGCA TCGTGGTGAT TGATGAAACT GCTGCTGTCG GCTTTAACCT CTCTTTAGGC
6481 ATTGGTTTCG AAGCGGGCAA CAAGCCGAAA GAACTGTACA GCGAAGAGGC AGTCAACGGG
6541 GAAACTCAGC AAGCGCACTT ACAGGCGATT AAAGAGCTGA TAGCGCGTGA CAAAAACCAC
6601 CCAAGCGTGG TGATGTGGAG TATTGCCAAC GAACCGGATA CCCGTCCGCA AGGTGCACGG
6661 GAATATTTCG CGCCACTGGC GGAAGCAACG CGTAAACTCG ACCCGACGCG TCCGATCACC
6721 TGCGTCAATG TAATGTTCTG CGACGCTCAC ACCGATACCA TCAGCGATCT CTTTGATGTG
6781 CTGTGCCTGA ACCGTTATTA CGGATGGTAT GTCCAAAGCG GCGATTTGGA AACGGCAGAG
6841 AAGGTACTGG AAAAAGAACT TCTGGCCTGG CAGGAGAAAC TGCATCAGCC GATTATCATC
6901 ACCGAATACG GCGTGGATAC GTTAGCCGGG CTGCACTCAA TGTACACCGA CATGTGGAGT

6961 GAAGAGTATC AGTGTGCATG GCTGGATATG TATCACCGCG TCTTTGATCG CGTCAGCGCC
7021 GTCGTCGGTG AACAGGTATG GAATTTCGCC GATTTTGCGA CCTCGCAAGG CATATTGCGC
7081 GTTGGCGGTA ACAAGAAAGG GATCTTCACT CGCGACCGCA AACCGAAGTC GGCGGCTTTT
7141 CTGCTGCAAA AACGCTGGAC TGGCATGAAC TTCGGTGAAA AACCGCAGCA GGGAGGCAAA
7201 CAATGAATCA ACAACTCTCC TGGCGCACCA TCGTCGGCTA CAGCCTCGGG AATTGCTACC
7261 GAGCTCGAAT TTCCCCGATC GTTCAAACAT TTGGCAATAA AGTTTCTTAA GATTGAATCC
7321 TGTTGCCGGT CTTGCGATGA TTATCATATA ATTTCTGTTG AATTACGTTA AGCATGTAAT
7381 AATTAACATG TAATGCATGA CGTTATTTAT GAGATGGGTT TTTATGATTA GAGTCCCGCA
7441 ATTATACATT TAATACGCGA TAGAAAACAA AATATAGCGC GCAAACTAGG ATAAATTATC
7501 GCGCGCGGTG TCATCTATGT TACTAGATCG GGAATTGGGG ATCTGCTGCC TCGCGCGTTT
7561 CGGTGATGAC GGTGAAAACC TCTGACACAT GCAGCTCCCG GAGACGGTCA CAGCTTGTCT
7621 GTAAGCGGAT GCCGGGAGCA GACAAGCCCG TCAGGGCGCG TCAGCGGGTG TTGGCGGGTG
7681 TCGGGGCGCA GCCATGACCC AGTCACGTAG CGATAGCGGA GTGTATACTG GCTTAACTAT
7741 GCGGCATCAG AGCAGATTGT ACTGAGAGTG CACCATATGC GGTGTGAAAT ACCGCACAGA
7801 TGCGTAAGGA GAAAATACCG CATCAGGCGC TCTTCCGCTT CCTCGCTCAC TGACTCGCTG
7861 CGCTCGGTCG TTCGGCTGCG GCGAGCGGTA TCAGCTCACT CAAAGGCGGT AATACGGTTA
7921 TCCACAGAAT CAGGGGATAA CGCAGGAAAG AACATGTGAG CAAAAGGCCA GCAAAAGGCC
7981 AGGAACCGTA AAAAGGCCGC GTTGCTGGCG TTTTTCCATA GGCTCCGCCC CCCTGACGAG
8041 CATCACAAAA ATCGACGCTC AAGTCAGAGG TGGCGAAACC CGACAGGACT ATAAAGATAC
8101 CAGGCGTTTC CCCCTGGAAG CTCCCTCGTG CGCTCTCCTG TTCCGACCCT GCCGCTTACC
8161 GGATACCTGT CCGCCTTTCT CCCTTCGGGA AGCGTGGCGC TTTCTCATAG CTCACGCTGT
8221 AGGTATCTCA GTTCGGTGTA GGTCGTTCGC TCCAAGCTGG GCTGTGTGCA CGAACCCCCC
8281 GTTCAGCCCG ACCGCTGCGC CTTATCCGGT AACTATCGTC TTGAGTCCAA CCCGGTAAGA
8341 CACGACTTAT CGCCACTGGC AGCAGCCACT GGTAACAGGA TTAGCAGAGC GAGGTATGTA
8401 GGCGGTGCTA CAGAGTTCTT GAAGTGGTGG CCTAACTACG GCTACACTAG AAGGACAGTA
8461 TTTGGTATCT GCGCTCTGCT GAAGCCAGTT ACCTTCGGAA AAAGAGTTGG TAGCTCTTGA
8521 TCCGGCAAAC AAACCACCGC TGGTAGCGGT GGTTTTTTTG TTTGCAAGCA GCAGATTACG
8581 CGCAGAAAAA AAGGATCTCA AGAAGATCCT TTGATCTTTT CTACGGGGTC TGACGCTCAG
8641 TGGAACGAAA ACTCACGTTA AGGGATTTTG GTCATGAGAT TATCAAAAAG GATCTTCACC
8701 TAGATCCTTT TAAATTAAAA ATGAAGTTTT AAATCAATCT AAAGTATATA TGAGTAAACT
8761 TGGTCTGACA GTTACCAATG CTTAATCAGT GAGGCACCTA TCTCAGCGAT CTGTCTATTT
8821 CGTTCATCCA TAGTTGCCTG ACTCCCCGTC GTGTAGATAA CTACGATACG GGAGGGCTTA
8881 CCATCTGGCC CCAGTGCTGC AATGATACCG CGAGACCCAC GCTCACCGGC TCCAGATTTA
8941 TCAGCAATAA ACCAGCCAGC CGGAAGGGCC GAGCGCAGAA GTGGTCCTGC AACTTTATCC
9001 GCCTCCATCC AGTCTATTAA TTGTTGCCGG GAAGCTAGAG TAAGTAGTTC GCCAGTTAAT
9061 AGTTTGCGCA ACGTTGTTGC CATTGCTGCA GGGGGGGGGG GGGGGGGGAC TTCCATTGTT
9121 CATTCCACGG ACAAAAACAG AGAAAGGAAA CGACAGAGGC CAAAAAGCCT CGCTTTCAGC
9181 ACCTGTCGTT TCCTTTCTTT TCAGAGGGTA TTTTAAATAA AAACATTAAG TTATGACGAA
9241 GAAGAACGGA AACGCCTTAA ACCGGAAAAT TTTCATAAAT AGCGAAAACC CGCGAGGTCG
9301 CCGCCCCGTA ACCTGTCGGA TCACCGGAAA GGACCCGTAA AGTGATAATG ATTATCATCT
9361 ACATATCACA ACGTGCGTGG AGGCCATCAA ACCACGTCAA ATAATCAATT ATGACGCAGG
9421 TATCGTATTA ATTGATCTGC ATCAACTTAA CGTAAAAACA ACTTCAGACA ATACAAATCA
9481 GCGACACTGA ATACGGGGCA ACCTCATGTC CCCCCCCCCC CCCCCCCTGC AGGCATCGTG
9541 GTGTCACGCT CGTCGTTTGG TATGGCTTCA TTCAGCTCCG GTTCCCAACG ATCAAGGCGA
9601 GTTACATGAT CCCCCATGTT GTGCAAAAAA GCGGTTAGCT CCTTCGGTCC TCCGATCGTT
9661 GTCAGAAGTA AGTTGGCCGC AGTGTTATCA CTCATGGTTA TGGCAGCACT GCATAATTCT
9721 CTTACTGTCA TGCCATCCGT AAGATGCTTT TCTGTGACTG GTGAGTACTC AACCAAGTCA
9781 TTCTGAGAAT AGTGTATGCG GCGACCGAGT TGCTCTTGCC CGGCGTCAAC ACGGGATAAT
9841 ACCGCGCCAC ATAGCAGAAC TTTAAAAGTG CTCATCATTG GAAAACGTTC TTCGGGGCGA
9901 AAACTCTCAA GGATCTTACC GCTGTTGAGA TCCAGTTCGA TGTAACCCAC TCGTGCACCC
9961 AACTGATCTT CAGCATCTTT TACTTTCACC AGCGTTTCTG GGTGAGCAAA AACAGGAAGG
10021 CAAAATGCCG CAAAAAAGGG AATAAGGGCG ACACGGAAAT GTTGAATACT CATACTCTTC
10081 CTTTTTCAAT ATTATTGAAG CATTTATCAG GGTTATTGTC TCATGAGCGG ATACATATTT
10141 GAATGTATTT AGAAAAATAA ACAAATAGGG GTTCCGCGCA CATTTCCCCG AAAAGTGCCA
10201 CCTGACGTCT AAGAAACCAT TATTATCATG ACATTAACCT ATAAAAATAG GCGTATCACG
10261 AGGCCCTTTC GTCTTCAAGA ATTCAGTACA TTAAAAACGT CCGCAATGTG TTATTAAGTT

10321 GTCTAAGCGT CAATTTGTTT ACACCACAAT ATATCCTGCC A//
