# Supplementary material for: Defining the ‘HoneySweet’ insertion event utilizing NextGen sequencing and a de novo genome assembly of plum (Prunus domestica)
Source: Hortic Res. 2021 Jan 1;8:8. doi: 10.1038/s41438-020-00438-2 (PMC7775438; doi:10.1038/s41438-020-00438-2)
Supplement: Supplementary file 3 — Supplementary Data Set 2 [file 41438_2020_438_MOESM3_ESM.pdf]

Supplementary Data Set 2. Alignment of five plum scaffolds from “Improved French assembly v 1.0” with sequence flanking insertion 1 in ‘HoneySweet’ (HS). Approximately 1000 bases upstream and downstream of predicted insertion 1 were aligned. Blue colored bases are variants and the light blue box represents the insertion site and the bases that are deleted from the insertion event.

```
        0                                                                                                                      120
5'HS
2675    CGCTGAGTCACCAACAACATCTTAGCATGGATAAGATAACTGTAGTCTGTGTCTGTCTCGTTTGTCCAAATGCCACATCAGATAATTATAACTGGATTTTACGTTTCTTCTAAAGGGAAA
1234    CGCTGAGTCACCAACAACATCTTAGCATGGATAAGATAACTGTAGTCTGTGTCTGTCTCGTTTGTCCAAATGCCACATCAGATAATTATAACTGGATTTTACGTTTCTTCTAAAGGGAAA
1332    CGCTGAGTCACCAACAAAATCTTAGCATGGATAAGATAACTGTAGTCTCTGTGTGTCTCGTTTGTCCAAATGCCACATCAGATAATTATAGCTGGATTTTACGTTTCTTTTAAAGGGAAA
1429    ATTGAAACACCCTTATATGCTGGGCGTCAACACCGCTTCGTCCTCTCTGTGAGTGATGCAATCCTCTCGTTATGATGAAGGACAAGATATTTGTCAATTTACGTTTCTTTTAAAGGGAAA
1650    CGCCGAGTCACCAACAAAATCTTAGCATGGATAAGATAACTGTAGTCTCTGTGTGTCTCGTTTGTCCAAATGCCACATCAGATAATTATAGCTGGATTTTACGTTTCTTTTAAAGGGAAA

                                                                                                                               240
5'HS    AATCTTTATTGAAAAAGAAAAAACCAATTTTGCCCTAAAAACAAAACTTAAGAAAAAAAAA--TTCTAAAATTGATACATTGGCAGGCATGGGTTGAGCCAATTGTCTATGGTAGTGAGTT
2675    ATCTTTATTGAAAAAGAAAAAA-CCAATTTTGCCCTAAAAACAAAACTTAAGAAAAAAAAA--TTCTAAAATTGATACATTGGCAGGCATGGGTTGAGCCAATTGTCTATGGTAGTGAGTT
1234    ATCTTTATTGAAAAAGAAAAAAACCAATTTTGCCCTAAAAACAAAACTTAAGAAAAAAAAA--TTCTAAAATTGATACATTGGCAGGCATGGGTTGAGCCAATTGTCTATGGTAGTGAGTT
1332    ATCTTTATTGAAAAAGAAAAAA-CCAATTTTGCCCTAAAAACAAAACTTAAGAAAAAAAAA---TCTAAAATTGATACATTGGCAGGCATGGGTTGAGCCAATTGTCTATGGTAGTGAGTT
1429    ATCTTTATTGAAAAAGAAAATA-CCAATTTTGCCCTAAAAACAAAACTTAAGAAGAAAAAAAATCTAAAATTGATACATTGGCAGGCATGGGTTGAGCCAATTGTCTATGGTAGTGAGTT
1650    ATCTTTATTGAAAAAGAAAAAA-CCAATTTTGCCCTAAAAACAAAACTTAAGAAAAAAAAA---TCTATAATTGATACATTGGCAGGCATGGGTTGAGCCAATTGTCTATGGTAGTGAGTT

                                                                                                                               360
5'HS    GCTCCTTTCCACTCGAGTTTGAGTCTTGTAAATAATTTAAATTATCATTTGTATACAAATTTTAA-----------------AAATAA-TAATAAAATTGAAACACCCTTATATGCTGGG
2675    GCTCCTTTCCACTCGAGTTTGAGTCTTGTAAATAATTTAAATTATCATTTGTATACAAATTTTAA-----------------AAATAA-TAATAAAATTGAAACACCCTTATATGCTGGG
1234    GCTCCTTTCCACTCGAGTTTGAGTCTTGTAAATAATTTAAATTATCATTTGTATACAAATTT-AA-----------------AAAAAAATAATAAAATTGAAACACCCTTATATGCTGGG
1332    GCTCCCTTCCACTCAAGTTTGAGTCTTGTAAATAATTTAAATTAAAAATAATTTAAGTTATCATTTGTATACAAATTTTTTTTTAAAAAGGATAAAATTGAAACACCCTTATATGCTGGG
1429    GCTCCTTTCCACTCGAGTTTGAGTCTTGTAAATAATTTAAATTAAAAATAATTTAAACTATCACTTGTATAGAAATT--AAAAAAAAAAGAATAAAATTGAAACACCCTTATATGCTGGG
1650    GCTCCCTTCCACTCAAGTTTGAGTCTTGTAAATAATTTAAATTAAAAATAATTTAAGCTATCATTTGTATACAATTTTTTTTTAAAAAAGGATAAAATTGAAACACCCTTATATGCTGGG

                                                                                                                               480
5'HS    CGTCAACACCGCTTCGTCCTCTCTGTGAGTGATGCAATCCTCTCGTTATGATGAAGGACAAGATATTTGTCAATTTAAGTCACAAAATGAAAGAAAAAAAAAGGGGATCAAAGTTAGCAAG
2675    CGTCAACACCGCTTCGTCTTCTCTGTGAGTGATGCAATCCTCTCGTTATGATGAAGGACAAGATATTTGTCAATTTAAGTCACAAAATGAAAGAAAAAAAAAGGGGATCAAAGTTAGCAAG
1234    CGTCAACACCGCTTCGTCCTCTCTGTGAGTGATGCAATCCTCTCGTTATGATGAAGGACAAGATATCTGTCAATTTAAGTCACAAAATGAAAGAAAAAAAAAGGGGATCAAAGTTAGCAAG
1332    CGTCAACACCGCTTCATCCTCTCTGTGAGTGATGCAATCCTCTCGTTATGATGAAGGACAAGATATTTGTCAATTTAAGTCACAAAATGAAAGAAAAAAAAAGGGGATCAAAGTTAGCAAG
1429    TGTCAACACCGCTTCGTCCTCTCTGTGAGTGATGCAATCCTCTCGTTATGATGAAGGACAAGATATTTGTCAATTTAAGTCACAAAATGAAAGAAAAAAAACGGGGATCAAAGTTAGCAAG
1650    CGTCAACACCGCTTCATCCTCTCTGTGAGTGATGCAATCCTCTCGTTATGATGAAGGACAAGATATTTGTCAATTTAAGTCACAAAATGAAAGAAGAAAAAGGGGATCAAAGTTAGCAAG

                                                                                                                               600
5'HS    TAATCACAAAAGAAGAAAATATTAGGGGTGGGCATCGGAACTACAAAATCAAATTGAATTGAGCCGGAAAAAAATCGTGTTGATTAAAAAAGTCAAAACTGACACAAAAATGGGTCCAATT
2675    TAATCACAAAAGAAGAAAATATTAGGGGTGGGCATCGGAACTACAAAATCAAATTGAATTGAGCCGGAAAAAAATCGTGTTGATTAAAAAAGTCAAAACTGACACAAAAATGGGTCCAATT
1234    TAATCACAAAAGAAGAAAATATTAGGGGTGGGCATCGGAACTACAAAATCAAATTGAATTGAGCCGGAAAAAAATCGTGTTGATTAAAAAAGTCAAAACTGACACAAAAATGGGTCCAATT
1332    TAATCACAAAAGAAGAAAATATTAGGGGTGGGCATCGGAACTACAAAATCAAATTGAATCGAGCCGGAAAAAAATCGTGTTGATTAAAAAAGTCAAAACTGACACAAAAATGGGTCCAATT
1429    TAATCACAAAAGAAGAAAATATTAGGGGTGGGCATCAGAACTACAAAATCAAATTGAATCGAGCCGGAAAAAAATCGTGTTGATTAAAAAAGTCAAAACTGACACAAAAATGGGTCCAATT
1650    TAATCACAAAAGAAGAAAATATTAGGGGTGGGCATCGGAACTACAAAATCAAATTGAATCGATCCGGAAAAAAATCGTGTTGATTAAAAAAGTCAAAACTGACACAAAAATGGGTCCAATT

                                                                                                                               720
5'HS    TCGATTTTTCCACCTCCACAAACTAGACCGATATATTTATATATTTATATATTTAATATATGTTGCAGGATTGTAATTTCTTATGATGGAGGATTGTAATTTTGAATGAATTTGGTTTGG
2675    TCGATTTTTCCACCTCCACAAACTAGACCGATATATTTATATATTTATATATTTAATATATGTTGCAGGATTGTAATTTCTTATGATGGAGGATTGTAATTTTGAATGAATTTGGTTTGG
1234    TCGATTTTTCCACCTCCACAAACTAGACCGATATATTTATATATTT--------AATATATGTTGCAGGATTGTAATTTCTTATGATGGAGGATTGTAATTTTGAATGAATTTGGTTTGG
1332    TCGATTTTTCCACCTCCACAAACTAGACCGATATATTTATATATTT--------AATATATGTTGCAGGATTGTAATTTGTTATGATGGAGGATTGTAATTTTGAATGAATTTGGTTTGG
1429    TCGATTTTTCCACCTCCACAAACTAGACCGATATATTTATATATTT--------AATATATGTTGCAGGATTGTAATTTGTTATGATGGAGGATCGTAATTTTGAATGAATTTGGTTTGG
1650    TCGATTTTTCCACCTCCACAAACTAGACCGATATATTTATATATTT--------AATATATGTTGCAGGATTGTAATTTGTTATGATGGAGGATTGTAATTTTGAATGAATTTGGTTTGG
```

```
                                                                                                                                                 840
5'HS    TTGTAATTTGTTATGCATGAGGATTGTAATTTGTTATTATGGAGGATTGTAGTTCTGAAGATGGATTGTAATTTGTTATGTTGTGCTGTGTTGGATTGTAATTTGGTTGTATTTTTGTTT
2675    TTGTAATTTGTTATGCATGAGGATTGTAATTTGTTATTATGGAGGATTGTAGTTCTGAAGATGGATTGTAATTTGTTATGTTGTGCTGTGTTGGATTGTAATTTGGTTGTATTTTTGTTT
1234    TTGTAATTTGTTATGCATGAGGATTGTAATTTGTTATTATGGAGGATTGTAGTTCTGAAGATGGATTGTAATTTGTTATGTTGTGCTGTGTTGGATTGTAATTTGGTTGTATTTTTGTTT
1332    TTGTAATTTGTTATGCATGAGGATTGTAATTTGTTATTATGGAGGATTGTAGTTCTGAAGATGGATTGTAATTTGTTATGTTGTGCTGTGTTGGATTGTAATTTGGTTGTATTTTTGTTT
1429    TTGTAATTTGTTATGCATGAGGATTGTAATTTGTTATTATGGAGGATTGTAGTTCTGAAGATGGATTGTAATTTGTTATGTTGTGCTGTGTTGGATTGTAATTTGGTTGTATTTTTGTTT
1650    -----------------------TTGTAATTTGTTATTATGGAGGATTGTAGTTCTGAAGATGGATTGTAATTTGTTATGTTGTGCTGTGTTGGATTGTAATTTGGTTGCTTTTTTNNNN
                                                                                                                                                 960
5'HS    TTTGTTTTTTT--GCTTTTTGTGGTGTTAAAAGTTTGCTGGATGATGAATTTGGGCTTGGGCCTATTGGAGAATGAATGTTGAAAAGTTGTGTTAGGAAAAAATTAATTTTAATGGGCTCA
2675    TTTGTTTTTTT--GCTTTTTGTGGTGTTAAAAGTTTGCTGGATGATGAATTTGGGCTTGGGCCTATTGGAGAATGAATGTTGAAAAGTTGTGTTAGGAAAAAATTAATTTTAATGGGCTCA
1234    TTTGTTTTTTT--GCTTTTTGTGGTGTTAAAAGTTTGCTGGATGATGAATTTGGGCTTGGGCCTATTGGAGAATGAATGTTGAAAAGTTGTGTTAGGAAAAAATTAATTTTAATGGGCTCA
1332    TTTGTTTTTTT--GCTTTTTGTGGTGTTAAAAGTTTGCTGGATGATGAATTTGGGCTTGGGCCTATTGGAGAATGAATGTTGAAAAGTTGTGTTAGGAAAAAATTAATTTTAATGGGCTCA
1429    TTGTTTTTATTTTGCTTTTTGTGGTGTTAAAAGTTTGCTGGATGATGAATTTGAGCTTGAACCTATTGGAGAATGAATGTTGAAAAGTTGTGTTAGGAAAAAATTAATTTTAATGGGCTTA
1650    NNNNNNNNNNNNNNNNNNNNNNNNNNNNNNNNNNNNNNNNNNNNN--------------GGCCTATTGGAGAATGAATGTTGAAAAGTTGTGTTAGGAAAAAATTAATTTTAATGGGCTTA

                                                                                                                                                1080
5'HS    AGATAAGGCTGAAAAACAGGAGAAA-GTGAAATGCTTTGCG-----------------GAATAAAATTTTGGGCTAAGGCCCAAACTAGAAAACCGCCAGAATCAGATCAGAATCGGGCA
2675    AGATAAGGCTGAAAAACAGGAGAAA-GTGAAATGCTTTGCG-----------------GAATAAAATTTTGGGCTAAGGCCCAAACTAGAAAACCGCCAGAATCAGATCAGAATCGGGCA
1234    AGATAAGGCTGAAAAACAGGAGAAA-GTGAAATGCTTTGCG-----------------GAATAAAATTTTGGGCTAAGGCCCAAACTAGAAAACCGCCAGAATCAGATCAGAATCGGGCA
1332    AGATAAGGCTGAAAAACAGGAGAAA-GTGAAATGCTTTGCG-----------------GAATAAAATTTTGGGCTAAGGCCCAAACTAGAAAACCGCCAGAATCAGATCAGAATCGGGCA
1429    AGATAAGGCTGAAAAACAGGAGAAA-GTGAAATGAAATGGGCTCCAGCATGTTTTACTGAATAAAATTTTGGGCTAAGGCCCAAACTAGAAAACCGCCGGAATCAGATCAGAATCGGGCA
1650    AGATAAGGCTGAAAAACAGGAGAAAAGTGAAATGAAATGGGTTCCAGCATGTTTTACTGAATAAAATTTTGGGCTAAGGCCCAAACTAGAAAACCACCAGAATCAAATCAGAATCGGGCA

                                                                                                                                                1200
5'HS    GGTTCGGATTGATTCGGTTCTCCCAATGATTTTTT-GCCTAAATTGGACTGATGCTCTTTTTTGGTTCTAATGAAGAACTAGAC
2675    GGTTCGGATTGATTCGGTTCTCCCAATGATTTTTT-GCCTAAATTGGACTGATGCTCTTTTTTGGTTCTAATGAAGAACTAGACCGTGCCGAGTCTTATAGAAATATTATGC-TTTTTTT
1234    GGTTCGGATTGATTCGGTTCTCCCAATGATTTTTT-GCCTAAATTGGACTGATGCTCTTTTTTGGTTCTAATGAAGAACTAGACCGTGCCGAGTCTTATAGAAATATTATGC-TTTTTTT
1332    GGTTCGGATTGATTCGGTTCTCCCAATGATTTTTT-GCCTAAATTGGACTGATGCTCTTTTTTGGTTCTAATGAAGAACTAGACCGTGCCGAGTCTTATAGAAATATTATGC-TTTTTTT
1429    GGTTCGGATTGGTTCGGTTCTCCCAATGATTTTTT-GCCTAAATTGGACTGATGCTCTTTTTTGGTTCTAATGAAGAACTAGACCGTGCCGAGTCTTTTAGAAATATTATGCGTTTTTTT
1650    GGTTCGGATTGGTTCGGTTCTCCTAATGATTTTTTTGCCTAAATTGGACTGATGCTCTTTTTTGGTTCTAATGAAGAACTAGACCGTGCCGAGTCTTTTAGAAATATTATGC-TTTTATT
3'HS                                                                                                                          TT

                                                                                                                                                1320
2675    ---TTTGTCAAACGAAAATATTATGTTTAAGCACCAAGTCCTGTGCTAGAAAAAAGAAAAAGCCTATCATTGGGATTTTCATGTTATCTCAGCGGCTTTCAAGTAAAATGATTGTTGGTG
1234    ---TTTGTCAAACGAAAATATTATGTTTAAGCACCAAGTCCTGTGCTAGAAAAAAGAAAAAGCCTATCATTGGGATTTTCATGTTATCTCAGCGGCTTTCAAGTAAAATGATTGTTGGTG
1332    ---TTTGTCAAACGAAAATATTATGTTTAAGCACCAAGTCCTGTGCTAGAAAAAAGAAAAAGCCTATCATTGGGATTTTCATGTTATCTCAGCGGCTTTCAAGTAAAATGATTGTTGGTG
1429    ---TTGGTCAAATGAAAATATTATGTTTAAGCACCAAGTCCTGTGCTAGAAAAAAGAAAAAGCCTATCATTTGGATTTTCATGTTATCTCAGCAGCTTTCAAGTAAAATGATTGTTGGTG
1650    TTTTTGGTCAAACGAAAATATTATGTTTAAGCAGCAAGTCCTGTGCTAGAAAAAAGAAAAAGCCTATCATTAGGATTTTCATGTTATCTCAGTAGCTTTCAAGTAAAATGATTGTTGGTG
3'HS    ---TTGGTCAAACGAAAATATTATGTTTAAGCACCAAGTCCTGTGCTAGAAAAAAGAAAAAGCCTATCATTAGGATTTTCATGTTATCTCAGCAGCTTTCAAGTAAAATGATTGTTGGTG

                                                                                                                                                 1440
2675    TTATAAAATAGACAGAATATGAAAGTACCATTGAATATTGGGGTGGGATTGTATTTGCTCTTGTGGTA-TTACACTGATAAAATTTTCTCTCTCGGATAAAACGTTCTAACTCTCAAATTT
1234    TTATAAAATAGACAGAATATGAAAGTACCATTGAATATTGGGGTGGGATTGTATTTGCTCTTGTGGTA-TTACACTGATAAAATTTTCTCTCTCGGATAAAACGTTCTAACTCTCAAATTT
1332    TTATAAAATAGACAGAATATGAAAGTACCATTGAATATTGGGGTGGGATTGTATTTGCTCTTGTGGTA-TTACACTGATAAAATTTTCTCTCTCGGATAAAACGTTCTAACTCTCAAATTT
1429    TTATAAAATAGACAGAGTATGAAAGTACCACTGAATATTGGGGTGGGATTGTATTTGCTCTTGTGGTA-TTACACTGATAAAATTTTCCCTCTCGGATAAAACGTTCTAACTCTCAAATTT
1650    TTATAAAATAGACAGAGTATGAAAGTACCACTGAATATCGGGGTGGGATTGTATTTGCTCTTGTGGTA-TTACACTAATAAAATTTTCTCTCTCAGATAAAACGTTCTAACTCTCAAATTT
3'HS    TTATAAAATAGACAGAGTATGAAAGTACCACTGAATATCGGGGTGGGATTGTATTTGCTCTTGTGGTTATTACACTGATAGAATTTTCTCTCTCGGATAAAACTTTCTAACTCTCAAATTT

                                                                                                                                                1460
2675    CTCCCACTTTTCTCTTTGGATTGGTGTATCTTCTTCTTCAACTAAGGATCCCTTTTATAGGGAAAGTTACCAGAGCAAA--------TGCTTTGCGGAAGAATTGGAGGTGAAAACATTT
1234    CTCCCACTTTTCTCTTTGGATTGGTGTATCTTCTTCTTCAACTAAGGATCCCTTTTATAGGGAAAGTTACCAGAGCAAA--------TGCTTTGCGGAAGAATTGGAGGTGAAAACATTT
1332    CTCCCACTTTTCTCTTTGGATTGGTGTATCTTCTTCTTCAACTAAGGATCCCTTTTATAGGGAAAGTTACCAGAGCAAA--------TGCTTTGCGGAAGAATTGGAGGTGAAAACATTT
1429    CTCTCACTTTTCTCTTTGGATTGGTGTATCTTCTTCTTCAACTAAGAATCCCTTTTATAGGGAAAGTTACCAGAGCAAA--------TGCTTTGCGGAAGAATTGGAGGTGAAAACATTT
1650    CTCTCACTTTTCTCTTTGAATTGGTGTATCTTCTTCTTCAACTAAGGATCCCTTTTATAGGGAAAGTTACCAGAGCAAA--------TGCTTTGCGGAAGAATTGGAGGTGAAAACATTT
```

```
3'HS    CTCTCACTTTTCTCTTTGAATTGGTGTATCTTCTTCTTCAGCTGAGGATCCCTTTTATAGGGAAAGTTACCAGAGCAAAGCAGTTAATGCTTTGCGGAAGAATTGGAGGTGAAAACATTT

                                                                                                                           1780
2675    CACCCACCAAAAACAGTATTTCACTCACATCAATCTACTATTGCATGCACAAAAAGTTTTGGGAAATTGACATAGATAATCACAAAAGATAAACCATAATCAAATATACTCACATTATAA
1234    CACCCACCAAAAACAGTATTTCACTCACATCAATCTACTATTGCATGCACAAAAAGTTTTGGGAAATTGACATAGATAATCACAAAAGATAAACCATAATCAAATATACTCACATTATAA
1332    CACCCACCAAAAACAGTATTTCACTCACATCAATCTACTATTGCATGCACAAAAAGTTTTGGGAAATTGACATAGATAATCACAAAAGATAAACCATAATCAAATATACTCACATTATAA
1429    CACCCACCAAAAACAGTATTTCACTCACATCAATCTACTATTGCATGCACAAAAAGTCTTCCTTGCTACATCCACACACACGTTTCACAATAGTTGGCAGTATTTATATTTATTTTATT
1650    CAACCACCAAAAACAGTATTTCACTCACATCAATCTACTATTGCATGCACAAAAAGTCTTCCTTGCTACATCCACACACACGTTTCACAATAGTTGGCAATATTTATATTTATTTTATT
3'HS    CACCCACCAAAAACACTATTTCACTCACATCAATCTACTATTGCATGCACAAAAAG

                                                                                                                           1900
2675    ATTTCAATCAGAAATACTCACTTTGTAACATGAAAGTACCTAATTGCCCTCCATATAAATATATATGTATTGACCCTTTATCCCCTCAAAAATTCATTTTCTGAAACTCAATTTCTCCCA
1234    ATTTCAATCAGAAATACTCACTTTGTAACATGAAAGTACCTAATTGCCCTCCATATAAATATATATGTATTGACCCTTTATCCCCTCAAAAATTCATTTTCTGAAACTCAATTTCTCCCA
1332    ATTTCAATCAGAAATACTCACTTTGTAACATGAAAGTACCTAATTGCCCTCCATATAAATATATATGTATTGACCCTTTATCCCCTCAAAAATTCATTTTCTGAAACTCAATTTCTCCCA
1429    TTATAATGAAACAAATAGGCTTGTAAGATATTATTTTCCTGATAAAATATATTTCTTTTTATAAAGTATAGGATTTTATTGTTTACTTATCAGTTAAGTTTTAGGAATTTTAAAAGATAT
1650    TTATAATGAAACAAATAGGCTTGTAAGATATTATTTTCCTGATAAAATATATTTCTTTTTATAAAGTATAGGATTTTATTGTTTACTTATCAGTTAAGTTTTAGGAATTTTAAAAGATAT

                                                                                                                           2020
2675    ACTCTTCTCCTCTCTCGCACTCTCCCAAATCTTGCTCAGCCATGACTTCATAGAAAATAGAGAGAGTAACAGCCATGGCGACGACGTCGGTGGATTCTCCGAAGTCTGACCCGGACACT
1234    ACTCTTCTCCTCTCTCGCACTCTCCCAAATCTTGCTCAGCCATGACTTCATAGAAAATAGAGAGAGTAACAGCCATGGCGACGACGTCGGTGGATTCTCCGAAGTCTGACCCGGACACT
1332    ACTCTTCTCCTCTCTCGCACTCTCCCAAATCTTGCTCAGCCATGACTTCATAGAAAATAGAGAGAGTAACAGCCATGNNNNNNNNNNGTTGCTTTTTGTGGTGTTAAAAGTTTGCTGGAT
1429    GGGCTTAGCCAAGTTGGCTAGAGTGGATGTATTTCTCACGTTGCACTCTAGTTCGAATCCCCCTCCCCGTAATTTAAAGTAGAATATTGCATGTATCACAACAATATTTTAGGAACTTTA
1650    GAGCTTAGCCAAGTTGGTTAGAGTGGATGTACTTCTCACGTTGCACTCTAGTTCGAATCCCCCTCCCCGTAATTTAAAGTAGAATATTGCATGTATCACAACAATATTTTAGGAACTTTA
```
