# Supplementary material for: Defining the ‘HoneySweet’ insertion event utilizing NextGen sequencing and a de novo genome assembly of plum (Prunus domestica)
Source: Hortic Res. 2021 Jan 1;8:8. doi: 10.1038/s41438-020-00438-2 (PMC7775438; doi:10.1038/s41438-020-00438-2)
Supplement: Supplementary file 4 — Supplementary Data Set 3 [file 41438_2020_438_MOESM4_ESM.pdf]

**Fig. S6.** Alignment of seven plum scaffolds from "Improved French assembly v 1.0" with sequence flanking insertion 2 in 'HoneySweet' (HS). Approximately 1000 bases upstream and downstream of the insertion site are aligned. In this case, there were no good alignments upstream for some of the Scaffolds and those sequences are shaded in gray until they did have good alignments. Three of the Scaffolds had long insertions. The start of the insertion has asterisks and the end of the insertion where it again matches is shaded in yellow. The insertion sequence for 1429-2 is marked with a single asterisk and is shown at the bottom of the figure. The insertion sequence for 4101 and 1650 marked with a double asterisk are nearly identical and are shown at the bottom of the figure. Blue colored bases are variants and the light blue box represents the insertion site and the bases that are deleted from the insertion event.

```
        1                                                                                                                    120
3'HS    TTTGTTGACGGAATACATGAGC---TCAAAAGCAAGAGATTGTCAGTAATAGACTAAACAGGAACAAATGCATTTTGAACACTAGAAATCACCTCAGGCAAAATGATC-----ATCAAAA
4101    TTTGTTGACGGAATACATGAGC---TCAAAAGCAAGAGATTATCAGTACTAGACTGAACAGGAACAAATGGTTTTCGAAGACTAGAAATCACCTCAGGCAAAATGATC-----ATCAAAA
1650    TTTGTTGACGGAATACATGAGC---TCAAAAGCAAGAGATTATCAGTACTAGACTGAACAGGAACAAATGGTTTTCGAAGACTAGAAATCACCTCAGGCAAAATGATC-----ATCAAAA
2675    TTTAATTATGGCCGACACTTT----TAATAAGCAAGAGATTATCAATAATCGACTGAACATGAATAAATGCATTTTGAACACTAGAAATCACCTCAGGTAAAATGATC-----ATCAAAA
1234_1  TTTCGTCGCGCAAAGTTGCTTT-------GCGCGACCAATAAGTT-TCGTCGCGCAAA-GTGACTT--TGCGCGACGAATA--AAAAACTTCGTCGCGCAAAGTCTTT-----CTCCACG
1234_2  TTTGTTGACGAAATGCATGAGC---TCAAAAGCAAGAGATTATCAGTAATGGACTGAACAGGAACGAATGCATTTTGAACACTAGAAATCACCTCAGGCAAAATGATC-----ATAAAAA
1429_1  TGTCCTTGCGCGACGAAATCTTGTTCGTCGCGCAAACCGTAAAATGTAGTAGTGTAAACAGGAACAAATGCATTTTGAACACTAGAAATCACCTCAGGCAAAATGATC-----ATCAAAA
4359    TGTCCTTGCGCGACGAAATCTTGTTCGTCGCGCAAACCGTAAAATGTAGTAGTGTAAACAGGAACAAATGCATTTTGAACACTAGAAATCACCTCAGGCAAAATGATC-----ATCAAAA
1429_2  GAACGATCCATACTTACTTGTACTACGATTGCATATAGGGTTTAATTTGTGCGTTAGTTATTTCTCACGCCA-----AACACTAGAAATCACCTCAGGCAAAATGATC-----ATCAAAA
6796    TATCGCCGCAC-TCTATGCAATGGTTACTACTCAAATCCCCAAA--TTCTTTCCCGAACAAAAATTCA-ATTTTAAGTCCATTAAAATACTCTCGAGGTAAAATTGTTTTTTTCTTGAAA

                                                                                                                             240
3'HS    AATTATGTA---GAGTCTTTGCACCGAACTGAGATCTTGAACTC---------TAAGAAAGTGTCTTAAAGC---ATAGAATGTGTAAGGGTTTCTCAGACCCCGCACATTCCAATCCA-
4101    AATTATGTA---GAGTCTTTGCACCGAACTGAGATCTTGAACTC---------TAAGAAAGTGTCTTAAAGC---ATAGAATGTGTAAGGGTTTCTCAGAGCCCGCACATTCCAATCCA-
1650    AATTATGTA---GAGTCTTTGCACCGAACTGAGATCTTGAACTC---------TAAGAAAGTGTCTTAAAGC---ATAGAATGTGTAAGGGTTTCTCAGAGCCCGCACATTCCAATCCA-
2675    AATTATTTA---CATTCTTTGTACCGAACTGGGATCTTGAACTC---------TAAGAAAATGTCTTAAAGC---ATAGAATGTATAAGGATTTCTCATACCCCACACATTCCAATCCAT
1234_1  ATCTTTGCG---TGACGAATTCTGCGCGACGAAGTTTC-------------------TGTCGCGCTAAAGT----TTGTGCG-ACAAATATGTGTTTTGCGCGACGAAATTCTCTTCGT
1234_2  AATTATGTA---GAGTCTTTGCACTAAACTGGGATCTTGAACTC---------TAAGAAAGTGTCTTAAAGC---ATAGAATGTGTAAGGGTTTCTCAGACCCCGCACATTCCAATCCA-
1429_1  AATTATGTA---GAGTCTTTGCACCGAACTGAGATCTTGAACTC---------TAACAAAGTGTCTTAAAGC---ATAGAATGTGTAGGGGTTTCTCAGACCCCGCACATTCCAATCCAT
4359    AATTATGTA---GAGTCTTTGCACCGAACTGAGATC---------------------AAAGTGTCTTAAAGC---ATAGAATGTGTAGGGGTTTCTCAGACCCCGCACATTCCAATCCAC
1429_2  AATTATGTA---GAGTCTTTGCACCGAAGTGAGATCTTGAACTC---------TAAGAAAGTGTCTTAAAGC---ATAAAATGTGTAAGGGTTTCTCAGACCCCGGACATTCCAATCCAC
6796    AATTTAATAATTGAATATTAATTTAGGAAGGGGATATCACAATCATAATATGGCAAAAAGTCATTTTTGGTCCCTATAGAATGTGTAAGGGTTTCTCAGACCCCGCACATTCCAATCCAC

                                                                                                                             360
3'HS    CTAGGCTCATTGAAATTGACACATTCCAAT-CCATT-----AGAGCATGCACATTTGACTCATTTATCACCACTAGGTTTACGAGAAGAAGTTCCACAATATAGTTTATTTGGTCTACTA
4101    TTAGGCTCATTGAAATTGACACATTCCAAT-CCATT-----AGAGCATGCAAATTTGACTCATTTATCACCACTAGGTTTACGAGAAGAAGTTCCAGAATATAGTTTATTTGGTCTACTA
1650    TTAGGCTCATTGAAATTGACACATTCCAAT-CCATT-----AGAGCATGCAAATTTGACTCATTTATCACCACTAGGTTTACGAGAAGAAGTTCCAGAATATAGTTTATTTGGTCTACTA
2675    TTAGGCTCATTGAAATTGACACATTTCAAT-CCATT-----AGAGCATGCAAATTTGACTCATTTATC---ACTAGGTTTACGAGAAGAAGTTCCACAATATAGTTTATTTGGTCTACTA
1234_1  TCGCGCAAAGTG----TAAAATGTAGTAGTGCCATT-----AGAGCATGCAAATTTGACTCATTTATCACCACTAGGTTTACGAGAAGAAGTTCCACAATATAGTTTATTTGGTCTACTA
1234_2  CTAGGCTCATTGAAATTGACACATTCCAAT-CCAGT-----AGAGCATGCAAATTTGACTCATTTATCACCACTAGGTTTACGAGAAGAAGTTCCACAATATAGTTTATTTGGTCTACTA
1429_1  CTATGCTCATTGAAATTGACACATTCCAAT-CCATTCCATTAGAGCATGCACATTTGACTCATTTATCACCACTAGGTTTACGAGAAGAAGTTCCACAATATAGTTTATTTGGTCTACTA
4359    CTATGCTCATTGAAATTGACACATTCCAAT-CCATT-----AGAGCATGCACATTTGACTCATTTATCACCACTAGGTTTACGAGAAGAAGTTCCACAATATAGTTTATTTGGTCTACTA
1429_2  CTAGGCTCATTGAAATTGACACATTCCAAT-CCATT-----AGAGCATGCACATTTGACTCATTTATCACCACTAGGTTTACGAGAAGAAGTTCCACAATATAGTTTATTTGGTCTACTA
6796    CTAGGCTCATTGAAATTGACACATTCCAAT-CCATT-----AGAGCATGCACATTTGACTCATTTATCACCACTAGGTTTACGAGAAGAAGTTCCACAATATAGTTTATTTGGTCTACTA

                                                                                                                             480
3'HS    AATATTATTAAATTTCCTAAAATATTGGAATTGTTTTTTTTTT--CTTGGATTTGAATTGTTCTGTAGACACTGTAATAATTGTTCATGGTATATGGTTAATGGTTTTCAAAGAAGTATC
```

```
4101      AATATTATTAAATTTCCTAAAATATTTGAATTGTTTTTTTTT---CTTGGATTTGAATTGTTCTGTAGACATTGTAATAATTGTTCATGGTATATGGTTAATGGTTTTCAAAGAAGTATC
1650      AATATTATTAAATTTCCTAAAATATTTGAATTGTTTTTTTTTT--CTTGGATTTGAATTGTTCTGTAGACATTGTAATAATTGTTCATGGTATATGGTTAATGGTTTTCAAAGAAGAATC
2675      AATATTATTAAATTTCCTAAAATATTTGAATTGTTTTTTATTTT-CTTGTATTTGAATTGTTCTGTAGACGCTGTAATAATTGTTCATGGTATATGGTTAATGGTTTTGAAGAAGTCTC
1234_1    AATATTATTAAATTTCCTAAAATATTTGAATTGTTTTTTTTT---CTTGGATTTGAATTGTTCTGTAGACATTGTAATAATTGTTCATAGTATATGGTTAATGGTTTTCGAAGAAGAATC
1234_2    AATATTATTAAATTTCCTAAAATATTTGAAATTTTTTTTCTTT-CTTGGATTTGAATTGTTCTGTAGACGCTGTAATAATTGTTCATGGTATATGGTTAATGGTTTTTGAAGAAGTATC
1429_1    AATATTATTAAATTTCCTAAAATATTGGAATTGTTTTTTTTTTTTCTTGGATTTGAATTGTTCTGTAGACACTGTAATAATTGTTCATGGTATATGGTTAATGGTTTTCAAAGAAGTATC
4359      AATATTATTAAATTTCCTAAAATATTGGAATTGTTTTTTTTTTTTCTTGGATTTGAATTGTTCTGTAGACACTGTAATAATTGTTCATGGTATATGGTTAATGGTTTTCAAAGAAGTATC
1429_2    AATATTATTAAATTTCCTAAAATATTGGAATTGTTATTTTTTT--CTTGGATTTGAATT-TTCTGTAGACACTGTAATAATTGTTCATGGTATATGGTTAATGGTTTTCGAAGAAGTATC
6796      AATATTATTAAATTTCCTAAAATATTGGAATTGTTTTTTTTTT--CTTGGATTTGAATTGTTCTGTAGAAACTGTAATAATTGTTCATGGTATATGGTTAATGGTTTTCAAAGAAGTATC

                                                                                                                               600
3'HS      TGGTTTTCTTAACAACTATATACATGAGAATGTTCCAATTATGCATTAGTCTTAGAGCAGGTGGTCTTGAGTCATTGCAGTCTTAGA-------TAGAAGGTGTCTC----ATAAGCATC
4101      TGGTTTTCTTAACAACTATATACATGAGAGTGTTCCAATCATGCATTAGTCTTAGAGCAGGTGGCCTTGAGTCATTGCAGTCTT-GAACA----TAGATAGAAGGTGTCTCAGAAGCATC
1650      TGGTTTTCTTAACAACTATATACATGAGAGTGTTCCAATCATGCATTAGTCTTAGAGCAGGTGGCCTTGAGTCATTGCAGTCTT-GAACA----TAGATAGAAGGTGTCTCAGAAGCATC
2675      TGGTTTTCTTAACAACTATATGCATGAGAATGTTCCAATTATAGATTAGTCTTAGAGCAGGTGGCCTCGAGTCATTGCAGTCTT-GAACA----TAGAAGGTGTCTC----AGAAGCGTC
1234_1    TGGTTTTCTTAACAACTATATACATGAGAGTGTTCCAATCATGCATTAGTCTTAGAGCAGGTGGCCTTGAGTCATTGCAGTCTT-GAACATAGATAGAAGGTGTCTC----AGAAGCATC
1234_2    TGGTTTTCTTAACAACTATATACATGAGAATGTTCCAATCATGCATTAGTCTTAGAGCAGGTGGCCTTGAGTCATTGCAGTCTTGAACATAACATAGATAGAAGGTGTCTCAGAAGCATC
1429_1    TGGTTTTCTTAACAACTATATACATGAGAATGTTCCAATTATGCATTAGTCTTAGAGCAGGTGGTCTTGAGTCATTGCAGTCTTAGA-------TAGAAGGTGTCTC----ATAAGCATC
4359      TGGTTTTCTTAACAACTATATACATGAGAATGTTCCAATTATGCATTAGTCTTAGAGCAGGTGGTCTTGAGTCATTGCAGTCTTAGA-------TAGAAGGTGTCTC----ATAAGCATC
1429_2    TGGTTTTCTTAACAACTATATACATGAGAATGTTCCAATTATGCATTAGTCTTACAGCAGGTGGCCTTGAGTCATTGCAGTCAT-GAACA----TAGATAGAAGGTGTCTCATAAGCATC
6796      GGGTTTTCTTAACAACTATATACATGAGAATGTTCCAATTATGCATTAGTCTTAGAGCAGGTGGCCATGAGTCATTGCAGTCTTAGA-------TAGAAGGTGTCTC----ATAAGCATC
                                                                                                                              720
3'HS      AGAAGGGAAATGAAATGTAACTTTGCACGTTATTTTCTTCATTTCAGCAACTTGACGCCAGACCTTTCGTCAGATGCAG---AAACTTGACGTGCATCCCACACGTGCATGTTATTACAT
4101      AGAAGGGAAATGAAATGTAACTTTGCACGTTATTTTCTTCATTTCAGCAACTTGACGCCAGACCTTTCGTCAGATGCAG---AAACTTGACGTGCATCCCACACGTGCATGTTATTACAT
1650      AGAAGGGAAATGAAATGTAACTTTGCACGTTATTTTCTTCATTTCAGCAACTTGACGCCAGACCTTTCGTCAGATGCAG---AAACTTGACGTGCATCCCACACGTGCATGTTATTACAT
2675      AGAAGGGAAATGAAATGTAACTTTACACGTTATTTTCTTCATTTCAGCAGCTTGACGCCAGCCCTTTCGTCAGATGCAG---AAACTTGACGTGCATCCCACACGTGCATGTTATTACAT
1234_1    AGAAGGGAAATGAAATGTAACTTTGCACGTTATTTTCTTCATTTCAGCAACTTGACGCCAGACCTTTCGTCAGATGCAG---AAACTTGACGTGCATCCCACACGTGCATGTTATTACAT
1234_2    AGAGGGGAAATGAAATGTAACTTTGCACGTTATTTTCTTCATTTCAGCAACTTGACGCCAGCCCTTTCGTCAGATGCAGCAGAAACTTGACGTGCATCCCACACTTGCATGTTATTTCAT
1429_1    AGAAGGGAAATGAAATGTAACTTTGCACGTTATTTTCTTCATTTCAGCAACTTGACGCCAGACCTTTCGTCAGATGCAG---AAACTTGACGTGCATCCCACACGTGCATGTTATTACAT
4359      AGAAGGGAAATGAAATGTAACTTTGCACGTTATTTTCTTCATTTCAGCAACTTGACGCCAGACCTTTCGTCAGATGCAG---AAACTTGACGTGCATCCCACACGTGCATGTTATTACAT
1429_2    AGAAGGGAAATGAAAAGTAACTTTGCACGTTATTTTCTTCATTTCAGCAACTTGACGCCAGACCTTTCGTCAGATGCAG---AAACTTGATGTGCATCCCACACGTGCATGTTATTACAT
6796      AGAAGGGAAATGAAATGCAACTTTGCACGTTATTTTCTTCATTTCAGCAACTTGACGCCAGACCTTTGGTCAGATGCAG---AAACTTGACGTGCATCCCACACGTACATGTTATTACAT
                                                                                 Insertion Site                               840
3'HS      TAACTTTGTTTAATGTAAGCAGTAGGGCGGGCCACACGTGCACGGTGCATCCCAAGAAAGAATTCAT
4101      TAACTTTGTTTAATGTAAGCAGTAG    GCCCACACGTGCACGGTGCATCCCAAGAAAGAATTCATTTGTCCTAATTTTATATATAGATTGTTGGTCGTCAGACTTTTCCTCGCCATAG
1650      TAACTTTGTTTAATGTAAGCAGTAG----GCCCACACGTGCACGGTGCATCCCAAGAAAGAATTCATTTGTCCTAATTTTATATATAGATTGTTGGTCGTCAGACTTTTCCTCGCCATAG
2675      TAACTTTGTTTAATGTAAGCAGTAG----GCCCACACGTGCACGGTGCATCCCAAGAAAGATTTAATTTGTTCTAATTTTATATATAGATTGTTGGTCGTCAGACCTTTCCTTGCCATA-
1234_1    TAACTTTGTTTAATGTCAGCAGTAG----GCCCACACGTGCACGGTGCATCCCAAGAAAGAATTCATTTGTCCTAATTTTATATATAGATTGTTGGTCGTCAGACTTTTCCTTGCCATAG
1234_2    TAACTTTGTTTAATGTAAGCAGTAG----GCCCACACGTGCACGGTGCATCCCAAAAAAAGAATTCATTTGTCCTAATTTTATATATAGATTGTTGGTCGTCAGACTTTTCCTTGCCATAG
1429_1    TAACTTTGTTTAATGTAAGCAGTAGGGCGGGCCACACGTGCACGGTGCATCCCAAGAAAGAATTCATTTGTTCTACTTTTATATATAGATTGTTGGTCGTCAGACTTTTCCTTGCCATAG
4359      TAACTTTGTTTAATGTAAGCAGTAGGGCGGGCCACACGTGCACGGTGCATCCCAAGAAAGAATTCATTTGTTCTACTTTTATATATAGATTGTTGGTCGTCAGACTTTTCCTTGCCATAG
1429_2    TAACTTTGTTTAATGTAAGCAGTAG----GGCCACACGTGCACGGTGCATCCCAAGAAAGAATTCATTTGTTCTAATTTTATATATAGATTGTTGGTCGTCAGAGTTTTCCTTGCCATAG
6796      TAACTTTGTTTAATGTAAGCAGTAG----GGCCACACGTGCACGGTGCATCCGAAGAAAGAATTCATTTGTTCTAATTTTATATATAGATTGTTGGTCGTCAGACTTTTCCTTGCCATAG
5'HS                                                                                                              TTTCCTTGCCATAG
                                                                                                                              960
4101      TTGGTAG**GAAATGAATTCCTTGTTGCTTCTGCAATGCACATTTTATGCGCACGGGACAGGACTGGC------------------GTTGAAATAATATAAGAAA-TGCAGAACCATTTTTT
1650      TTGGTAG**GAAATGAATTCCTTGTTGCTTCTGCAATGCACATTTTATGCGCACGGGACAGGACTGGC------------------GTTGAAATAATATAAGAAA-TGCAGAACCATTTTTT
2675      CTGGTGG--GAAATGAATTCCTTGTTGCTTCTGCAATGCA-TTTTATTTGCACGGGACAGGACTGGCCTTTAATGGGGGAATAGCGTTGAAATAATATAAGAAA-TGCAGAACCATTTTTT
1234_1    TTGGTAG--GAAATGAATTCCTTGTTGCTTATGCAATGCACATTTTATGCGCACGGGACAGGACTGGC------------------GTTGAAATAATATAAGAAA-TGCAGAACCATTTTTT
1234_2    TTGGTAG--GAAATGAATTCCTTGTTGCTTCTGCAAAGCACATTTTATGCGCACGGGACAGGACTGGC------------------GTTGAAATAATATAAGAAA-TGCAGAACCATTTTTT
1429_1    TTTGTAG--GAAATGAATTCCTTGTTGCTTCTGCAATGCACATTTTATGCGCACGGGACAGGACTGGC------------------GTTGAAATAATATAAGAAA-TGCAGA-CCATTTTTT
4359      TTTGTAG--GAAATGAATTCCTTGTTGCTTCTGCAATGCACATTTTATGCGCACGGGACAGGACTGGC------------------GTTGAAATAATATAAGAAA-TGCAGA-CCATTTTTT
1429_2    TTGGTAG--GAAATGAATTCCTTGTTGCTTCTGCAATGCGCATTTTATGCGCACGGGACAGGACTGGC------------------GTTGAA-TAATATAAGAAAATGCAGACCATTTTTT-
6796      TTGGTAG--GAAATGAATTCCTTGTTGCTTCTGCAATGCACATTTTATGCGCACGGGACAGGACTGGC------------------GTTGAAATAATATAAGAAA-TGCAGA-CCATTTTTT
5'HS      TTGGTAG--GAAATGAATTCCTTGTTGCTTCTGCAATGCACATTTTATGCGCACGGGACAGGACTGGC------------------GTTGAAATAATATAAGAAA-TGCAGA-CCATTTTTT
```

```
                                                                                                                                          1080

4101     GACTAATTTTGTTGCGGTCACAAATGACGGTTTTGTGGTACTTCCAAAATACACATTTTCA GGGTTAGGTAACACCACATTTTATTTTGGGTACTC-GAAAGTCGCCCTTAGTCTTCATT
1650     GACTAATTTTGTTGCGGTCACAAATGACGGTTTTGTGGTACTTCCAAAATACACATTTTCA GGGTTAGGTAACACCACATTTTATTTTGGGTACTC-GAAAGTCGCCCTTAGTCTTCATT
2675     GACTAATTTTGTTGCGGTCACGAATGACGGTTTTGTGGTACTTCCAAAATACACATTT-CA GGGTTAGGTAA-ACCACAATTTATTTTGGGTACTC-GAAAGTCGCCATTAGTCTTCATT
1234_1   GACTAATTTTGTTGCGGTCACAAATGACGGTTTTGTGGTACTTCCAAAATACACATTTTCA GGGTTAGGTAACACCACATTTTATTTTGGGTACTC-GAAAGTCGCCCTTAGTCTTCATT
1234-2   GACTAATTTTGTTGCGGTCACAAATGACGGTTTTGTGGTTCTTCCAAAATACACATTTTCA GGGTTAGGTAACACCATATTTTATTTTGGGTATTC-GAAAGTCGCCCTTAGTCTTCATT
1429_1   GACTAATTTTGTTGCGGTCACAAATGACGGTTTTGTGGTACTTCCAAAATACACATTTTCA GGGTTAGGTAACACCACATTTTATTTTGGGTACTC-GAAAGTCGCCCTTAGTCTTCATT
4359     GACTAATTTTGTTGCGGTCACAAATGACGGTTTTGTGGTACTTCCAAAATACACATTTTCA GGGTTAGGTAACACCACATTTTATTTTGGGTACTC-GAAAGTCGCCCTTAGTCTTCATT
1429_2   GACTAATTTTGTTGCGGTCACAAATGACGGTTTTGTGGTACTTCCAAAATACACATTTTCA*AGGTTAGGTAACACCACATTTTATTTTGGGTACTC-GAAAGTCGCCCTTAGTCTTCATT
6796     GACTAATTTTGTTGCGGTCACAAACGACGGTTTTGTGGTACTTCCAAAATACACATTTTCA GGGTTGGGTAACACCACATTTTATTTTGGGTACTC-GAAAGTCGCCCTTAGTCTTCATT
5'HS     GACTAATTTTGTTGCGGTCACAAATGACGGTTTTGTGGTACTTCCAAAATACACATTTTCA GGGTTAGGTAACACCACATTTTATTTTGGGTACTCTGAAAGTCGCCCTTAGTCTTCATT

                                                                                                                                          1200
4101     TTTCTTCATCCTTTTTTT-TCCTGTCTCATGCGAACTAAAAGCGTAGGGTCCTATCACAAGAGATCTTAATCCTTAATCCAATATGTGGTGTTCACATGATGTACTTGAATATTTACTCT
1650     TTTCTTCATCCTTTTTTT-TCCTGTCTCATGCGAACTAAAAGTGTAGGGTCCTATCACAAGAGATCTTAATCCTTAATCCAATATGTGGTGTTCACATGATGTACTTGAATATTTACTCT
2675     TTT---------TTTTCT-TTCTGTCGCATGCGAACTAAAA-TGTAGGGTCCTATCACAAGAGATCTTAATTCTTAATCCAATATGTGGTGTTCACATAATGTACTTGAATATTTACTCT
1234_1   TTTCTCCATCCTTTTTTTATCCTGTCTCGTGCGAACTAAAAGCGTAGGGTCCTATCACAAGTGATCTTAATCCTTAATCCAATATGTGGTGTTCACATGATGTACTTGAATATTTACTCT
1234-2   TTTCTTCATCCTTTTTTT-TCCTGTCTCGTGCGAACTAAAAGCATAGGGTCCTATCACAAGAGATCTTAATCCTTAATCCAATATATGATGTTCACATGATGAATATTTACTCATACTCC
1429_1   TTTCTTCATCCTTTTTTT-TCCTGTCTCGTGCGAACTAAAAGCGTAGGGTCCTATCACAAGAGATCTTAATTCTTAACCCAATATGTGATGTTCACATGATCAATATTTACTCTTACCCC
4359     TTTCTTCATCCTTTTTTT-TCCTGTCTCGTGCGAACTAAAAGCGTAGGGTCCTATCACAAGAGATCTTAATTCTTAACCCAATATGTGATGTTCACATGATCAATATTTACTCTTACCCC
1429_2   TTTCTTCATCCTTTTTTT-TCTTGTCTCGTGCGAACTAAAAGCGTAGGGTCCTATCACAAGAGATCTTAATCCTTAACCCAATATGTGATGTTCACATGATGAATATTTACTCTTACCCC
6796     TTTCTTCATCCTTTTTTT-TCCTGCCTCGTGCGAACTAAAAGCGTAGGGTCCTATCACAAGAGATCTTAATCTTTAACCCAATATGTGATGTTCACATGATGAATATTTACTCTTACCCC
5'HS     TTTCTTCATCCTTTTTTTTTCCTGTCTCGTGCGAACTAAAAGCGTAGGGTCCTATCACAAGAGATCTTAATTCTTAACCCAATATGTGATGTTCACATGATGAATATTTACTCTTACCCC
                                                                                                                                          1320
4101     -TACCCCTTACTCAATACGTGATGTTAACGAGTTGGGCTTGAATGATT--GACTCCACCGTGAATTTGACTGCATATGTGGCCCATTACGTGACGGTCGTACATAAGAAATAAAGATAATA
1650     -TACCCCTTACTCAATACGTGATGTTAACGAGTTGGGCTTGAATGATT--GACTCCACCGTGAATTTGACTGCATATGTGGCCCATTACGTGACGGTCGTACATAAGAAATAAAGATAATA
2675     -CACCCCTTACTCAATATGTGGTGTTAACGAGTTGGGCTTGAATGATTTTGACTTCATTGTGAATTTGGCTGCATGTGTGGCCCATTATGTGGCGGTCGTACATAAGAAATAAAAAATATA
1234_1   -TACCCCTTATTCAATACGTGATGTTAACGAGTTGGGCTTGAATGATT--GACTCCATCGTGAATTTGACTGCATATGTGGCCCATTACGTGACGGTCGTACATAAGAAATAAAGATAATA
1234-2   TTACTCAATACGCGGTGTTAACGAGATGGACTTGAATGATG-GACTCCACCGTGAATTTGGATGCATATGTGGCCCATTACGTGACGGTCGTATA--------TAAGAAATAAAGATAATA
1429_1   TTACTCAATACGCGGTGTTAATGAGTTGGACTTGAATGATT-GAATCCACCATGAATTTGGCTGCATATGTGGCCCATTACGTGACGGTCGTACA--------TAAGAAATAAAGATAATA
4359     TTACTCAATACGCGGTGTTAATGAGTTGGACTTGAATGATT-GAATCCACCATGAATTTGGCTGCATATGTGGCCCATTACGTGACGGTCGTACA--------TAAGAAATAAAGATAATA
1429_2   TTACTCAATACGCGGTGTTAACGAGTTGGACTTGAATGATT-AACTCCACCATGAATTTGGCTGCATATGTGGCCCATTACGTGACGGTCGTACA--------TAAGAAATAAAGATAATA
6796     TTACTCAATACGCGGTGTTAATGAGTTGGACTTGAATGATT-GACTCCACCATGAATTTGGCTGCATATGTGGCCAATTACGTGACGGTCGTACA--------TAAGAAATAAAGATAATA
5'HS     TTACTTAATACACGGTGTTAACGAGTTGGACTTGAATGATT-GACTCCACCATGAATTTGGCTGCATATGTGGCCCATTACGTGACGGTCGTACA--------TAAGAAATAAAGATAATA
                                                                                                                                          1440
4101     TAT--ATTGAATGGGATCCACTACCACTACCACTCAGATCTTGGAATCTGTGAGTGATTAAGTTTGGGACAATATCGGTATTGCTAGTAGTGGGTTGTTGGTTTGTCTATTTGAAAACTT
1650     TAT--ATTGAATGGGATCCACTACCACTACCACTCAGATCTTGGAATCTGTGAGTGATTAAGTTTGGGACAATATCGGTATTGCTAGTAGTGGGTTGTTGGTTTGTCTATTTGAAAACTT
2675     TATTGATTGAATGGGATCCACTACTACTAGCACTCAGATTTTGGAATCTGTAAGTGGTTGAGTTTGGGACAATATCGGTGTTGTTAGTAGTGGACTGTTGGTTTATCTATTTGAAAACTT
1234_1   TAT--ATTGAATGGGATCCACTACCACTACCATTCAGATCTTGGAATCTGTGAGTGATTAAGTTTGGGACAATATCGGTATTGCTAGTAGTGGGTTGTTGGTTTGTCTATTTGAAAACTT
1234-2   TAT--ATTGAATGAGATCCACTACCACTACCACTCAGATCTTGGAATCTGTGAGTGGTTAAATTTGGGACAATATCGGTGTTGCTAGTAGTGGGTTGTTGGTCTGCCTATTTAAGAACTT
1429_1   TAT--ATTGAATGGGATCCACTACCACTACCACTCAGATCTTGGAATCTGTGAGTGATTAAGTTTGGGACAATATCGATGTTGCTAGTAGTGGGTTGTTGGTCTGTCTATTTGAAAACTT
4359     TAT--ATTGAATGGGATCCACTACCACTACCACTCAGATCTTGGAATCTGTGAGTGATTAAGTTTGGGACAATATCGATATTGCTAGTAGTGGGTTGTTGGTCTGTCTATTTGAAAACTT
1429_2   TAT--ATTGAATGGGATCCACTACCACTACCACTCAGATCTTGGAATCTATGAGTGGTTAAGTTTGGGACAATATCGGTGTTGCTAGTAGTGGGTTGTTGGTCTGTCTATTTGAAAACTT
6796     TAT--ATTGAATGGGATCCACTACCACTACCACTCAGATCTTGGAATCTGTGAGTGGTTAAGTTTGGGATAATATCGGTGTTGCTAGTAGTGGGTTGTTGGTCTGTCTATTTGAAAACTT
5'HS     TAT--ATTGAATGGGATCCACTACCACTACCACTCAGATCTTGGAATTTGTGAGTGATTAAGTTTGGGACAATATCGGTGTTGCTAGTAGTGGGTTGTTGGTTTGTCTATTTGAAAACTT
                                                                                                                                          1560
4101     AACACTATTCAGACTCGCTTTTGA--CTCTCTCTTGAAGAACCAAGGATATGAAAGTAAAACCCCTAAGCTTACTTCACCACTAGTT--TCGCATCGGGTTCTTGTAATTTTCTATAATT
1650     AACACTATTCAGACTCGCTTTTGA--CTCTCTCTTGAAGAACCAAGGATATGAAAGTAAAACCCCTAAGCTTACTTCACCACTAGTT--TCGCATCGGGTTCTTGTAATTTTCTATAATT
```

```
2675     AACACTATTCAAACTCGCTTTTGAGACTCTCTCTTGAAGAACCAAGGATATGAAAGTAAA-CCCCTAAGCTTACTTCACCACTAGTT--TCACATTGGGTTCTTGTAATTTTCTATAATT
1234_1   AACACTATTCAGACTCGCTTTTGA--CTCTCTCTTGAAGAACCAAGGATATGAAAGTAAAACCCCTAAGCTTACTTCACCACTAGTT--TCGCATTGGGTCCTTGTAATTTTCTATAATT
1234-2   AACACTATTCAGACTCGCTTTTGA--CTCTCTCTTGAAGAACCAAGGATACGAAAGTAAAACCCCTAAGCTTACTTCACCACTAGTT--TCACATCGGGTTCTTGTAATTTTCTATAAAT
1429_1   AACACTATTCAGACTCGCTTTTGA--CCCTCTCTTGAAGAACCAAGGATATGAAAGTAAAACCCCTAAGA--GCTTCACCACTAGTT--TAACATCGGGTTCTTGTAATTTTCTATAATT
4359     AACACTATTCAGACTCGCTTTTGA--CCCTCTCTTGAAGAACCAAGGATATGAAAGTAAAACCCCTAAGA--GCTTCACCACTAGTT--TAACATCGGGTTCTTGTAATTTTCTATAATT
1429_2   AACACTATTTAGACTCGCTTTTGA--CCCTCTCTTGAAAAACCAAGGATATGAAAGTAAAACCCCTAAGA--GCTTCACCACTCACTGGTAAAAAATAACCCTTGTG--CGACCAAATTT
6796     AACACTATTCAGACTCACTTTTGA--CCCTCTCTTGAAGAACCAAGGATATGAAAGTAAAACCCCTAAGAT--CTTCACCACAC--TAGTAAAAAATAACCCTTGTG--CGACCAAATTT
5'HS     AACACTATTCAGACTCGCTTTTGA--CCCTCTCTTGAAGAACCAAGGATATGAAAGTAAAACCCCTAAGA--GCTTCACCACTCACTAGTAAAAAATAACCCTTGTG--CGACCAAATTT
                                                                                                                             1780
4101     TGCATT-TAGAACTCTATTAGAAGCCAAATATCTTCTACTATCATGAAGAAGATATCAGTGGGACAGATATCTTCTACTACCATTGGGACAAAAGGCTTCTG--GTTCCTCTTTTTAAAT
1650     TGCATT-TAGAACTCTATTAGAAGCCAAATATCTTCTACTATCATGAAGAAGATATCAGTGGGACAGATATCTTCTACTACCATTGGGACAAAAGGCTTCTG--GTTCCTCTTTTTAAAT
2675     TGCATT-TAGAACTCTATTAGAAGCCAAATATCTTCTACTTTCATGAAGAAGATATCAATGGGACGGATATCTTCTACTACCATTGGGACAAAAGGCTTCTGTTTTTCTTCTTTTTAAAT
1234_1   TGCATT-TAGAACTCTATTAGAAGCCAAATATCTTCTACTATCATGAAGAAGATATCAGTGGGACATATATCTTCTACTACCATTGGAACAAAAGGCTTCTG--TTTCCTCTTTTTAAAT
1234-2   TGCATT-TAGAACTCTATTAGAAGCCAAATATCTTCTACTATCATGAAGAAGATATCAGTGGGACAGATATCTTCTACTACCATTGGGACAAAAGGCTTCTG--TTTCCTCTTTTTAAAT
1429_1   TGCATT-TAGAACTCTATTAGAAGCCAAATACCTTC--CTAACATGCAGTCAGCAT-GTTAGGAAGGTTTCAGTCAAATCCTGGTCAAGCACA-------------TTGGGTTGCAGGA
4359     TGCATT-TAGAACTCTATTAGAAGCCAAATACCTTC--CTAACATGCAGTCAGCAT-GTTAGGAAGGTTTCAGTCAAATCCTGGTCAAGCACA-------------TTGGGTTGCAGGA
1429_2   TGCGCGACGAAAAACTATTCGTCGCTTAAAGTCACT---TTGCGCGACAAAACT-----CAAAACTTCGTTGCTTAAAGTCTTGCGCGACCAA---------------ATTTTGCATGA
6796     TGCGCGACGAAAAACTATTCGTCGCTCAAAGTCACT---TTGCGTGACAAAACT-----CAAAACTTCGTTGCTCAAAGTCTTGCACGACCAA---------------ATTTTGCATGA
5'HS     TGCGCGACGAAAAACTATTCGTCGCTCAAAGTCACT---TTGCGCGACAAAACT-----CAAAACTTCGTTGCTCAAAGTCTTGCACGACCAA---------------ATTTTGCATGA

                                                                                                                              1900
4101     TTATCATTGTTGGGGTCACTTAGAAGAAAATATCAACAGATGTGGCCGTAATCCAATAATATTCTATAATTTCTCCTAAAAAAAGATTATACA---AGACTG TATAGATTATTTCTGGAA
1650     TTATCATTGTTGGGGTCACTTAGAAGAAAATATCAACAGATGTGGCCGTAATCCAATAATATTCTATAATTTCTCCTAAAAAAAGATTATACA---AGACTG TATAGATTATTTCTGGAA
2675     TTATCATTTTTGGGATCATTTAGAAGAAGATATCAACAGATGTGGCCATAATCCAATAATATTCTATAATTTCTCCTCTAAAAGGTTTCTACA---AGACTG TACAGATTATTTCCGGAA
1234_1   TTATAATTGTTGGGGTCACTTAGAAGAAGATATCAACAGATGTGGCCGTAATCCAATAATATTCTATAATTTCTCCTAAAAAAAGATTATACA---AGACTG TATAGATTATTTCTGGAA
1234-2   TTATCATTTTTGGGGTCACTTAGTAGAAGATATCAACAGATGTGGCCATAATCCAATAATATTCTATAATTTCTCCTAAAAAAAGATTATACA---AGACTC TATAGATTATTTCTGGAA
1429_1   AAGAAAGTCATGAGATACCTACAAAGAACAAAAGACTACAAGCTG--ATATTCAAAAAGGGTG--AAAGTTTGGAATTACAAGGATTTGCATATGCAGATTT TGCAGGTTGTC-----AA
4359     AAGAAAGTCATGAGATACCTACAAAGAACAAAAGACTACAAGCTG--ATATTCAAAAAGGGTG--AAAGTTTGGAATTACAAGGATTTGCAGATGCAGATTT TGCAGGTTGTC-----AA
1429_2   TGAAAAACAATTCG-T-GCGCAAAGTGACTTTGTGCGACAAACTT-----TTGCGCGACGACAATACATCTTCGCTCAAAGTGACTTTGAGCGACAAAACAA TAAACTTCGTCGAGCAAA
6796     TGAAAAACAATTCG-TCGCGCAAAGTGACTTTGTGCGACAAACTT-----TTGAGCGACGACAATACATTGTCGCTCAAAGTGAATTTGCGCGACAAAACAA TAAACTTCGTCGAGCAAA
5'HS     CGAAAAACAATTCG-TCGCGCAAAGTGACTCTGATGGTTGGATCG-----TTGAATGGTGTGTGTATATATATATATATATATTTATTAAGTAGCTTTAAATTTATTTATTTTGTACGT
```

## Intervening Sequences

```
*1429_2 TAGGGGTGGGCACGGTCCGGATTGGTTCGGTTTTACTCTCAAACCACAGCTGAACCAATAAGAAAATAACGGTCCGGTTTGGTTCGAATTAGCCTAAAATCATAATGAAAACCGAACCAAT
CCAAACCACTTGTAATCGGTTTGGTTCGGATTGGTTCGGCCGGTTTGTGCCTAACACAAAATCATGAATTTTTCAACCCGCTCTTATATTATAAATTATAATGCTAACATGTTGTCATTATATCATTCC
ATTCCATCAAAGGCTAACACAACAACATTCTATTTAATCAAAAGCTGTCCAATTTTCAGCATGCATCAAGTTCACAATTTCCATCAAAACATTCTAAAATTCATAAGAAATGAATACAAGATGAACTAC
AAAATGAAAGGCAGCCCAAAATACAAGTTCATACTTTCCATTTAAACAAAAGCTATCCAGACAGATTGCACCCAACAATTATTCTGCAGTAAGTCCAAACTCATGGAAATGCCAAAAGAACAAAAATGA
GAAATAGCACTTGTCTTTTCCTAGTTTGTTCCTTATATTTTTGAGCTATTTACTTTGTTTTTGTGTTTTGTAGGATCTATCAAGCAAAGAAAAGAAAAATAGCACAAGTGGGATTTTAAGTTACAAATT
CGTCAAAACTGCCTGTGCAGATGAGCTGACTTTGGAAGCAAGTTGCGAACAGCTCAGAAAGAATTAGAGAACGTGCCATATATGCTTGGAAAGCTACAGATGTCTATTCTCTGGAGCATTTCGCGGATT
ATTCATATCATGTTTCTAGAAGCAGTTATGGCCGTTTTAACACTGAAAGGTTCATAAATATATAATTAATTAATAGTTAAATGGTTCGGTTCGGATTAGCACGGTTTTCCAAGGCTCAAAACTGCAGCT
GAACCAAACTACTCCGGTTTGGTTCGGATTTTAAAACTGTTGACTTTTTTAAAGTCAAAACCAAACCAAACCGGAAAATACGGTTTGGTTCGGCCGGTTTGGTCGGATCGGCCGGATTGATGCCCACCC
CTAATTTTCAG

**4101  CATGGTCTGAAATATCGATAATATCGAGGAAATATCGAGGATATTTCGGTTTTTTTGAATCACGGATATTTCGAAACATATCCATGTACATATCGTATAAGATAATATCGGAAAATATCGA
**1650  CATGGTCTGAAATATCGATAATATCGAGGAAATATCGAGGATATTTCGGTTTTTTTGAATCACGGATATTTCGAAACATATCCATGTACATATCGTATAA----ATATCG-ATAATATCGA

**1650  CGATAATATCGGAAAATATCG

**4101  ATGTCAATAATTTCGCTCACACTTCAGCAATATTTTGTCAAAATATCGGTGTAATATCGCTAAAATATCGAAAATATCGATGTAAAGGAAAA GAGAAAAAAAGGGGGGAAAAGGGAGAAAA
**1650  ATGTCGATAATTTCGCTCACACTTCAGCAATATTTTGTCAAAATATCGGTGTAATATCGCTAAAATATCGAAAATATCGATGTAAAGGAAAAAGAGAAAAAAAGGGGGGAAAAGGGAGAAAA

**4101  AAAACACAAAGAGGATTTGAACCACTCCCATTTTACTCCTCCAACACCTTAACCACTATATCACTTATGTTTTTGTGATAATATGCTAAAATATTTATATTTATATTGGTTTGTTTTGAATT
**1650  AAAACACAAAGAGGATTTGAACCACTCCCATTTTACTCCTCCAACACCTTAACCACTATATCACTTATGTTTTTGTGATAATATGCTAAAATATTTATATTTATATTGGTTTGTTTTGAATT
```

```
**4101  CTTTTTGCAAAAATCAAATTTTCACATGAGAACGAATCTTTGACCTTTCCTTTGGGTGGCATGTTAACAATTACATGCAAAACTATTTTGGAGATTTATCATTTGATGACTACTCTTCACAA
**1650  CTTTTTGCAAAAATCAAATTTTCACATGAGAACAAATCTTTGACCTTTCCTTTGGGTGGCATGTTAACAATTACATGCAAAACTATTTTGGAGATTTATAATTTGATGACTACTCTTCACAA

**4101  TACACTTACTCTACACATAGAGATGATGAAGATAGTGAAAAATCTGAACCTCATAGGAACTCTATGTGGTACTAAGTCACTCATATATCTTATCATGCAATGTATACAGTGTAAAATATTGT
**1650  TACACTTACTCTACACATAGAGATGATGAAGATAGTGAAAAATCTGAACCTCATAGGAACTCTATGTGGTACTAAGTCACTCATGTATCTTATCATGCAATGTATACAGTGTAAAATATTGT

**4101  AGTAAATCTTATATATAAATTATTATGGTGTGTTTAATATTTTTTCATTAATTTCTACATATTTTCTACACTCATAGTGTTTGCCAGCGCTATACAATCAACTTAAATAAGTTAAATCCATC
**1650  AGTAAATCTTATATATAAATTATTATGGTGTGTTTAATATTTTTTCATTAATTTCTACATATTTTCTACACTCATAGTGTTTGCCAGCGCTATACAATCAACTTAAATAAGTTAAATCCATC

**4101  ATGCAATGCAATTCCTTCCAATTTTTTGTGATAAACTAATAGATAATTGACTAAATAAGCATCCTCCAAAGTTTCAATAAAAATTTCCAAGTTTTTCTTACAATTTCCGTGATTTTTATTCA
**1650  ATGCAATGCAATTCCTTCCAATTTTTTGTGATAAACTAATAGATAATTGACTAAATAAGCATCCTCCAAAGTTTCAATAAAAATTTCCAAGTTTTTCTTACAATTTCCGTGGTTTTTATTCA

**4101  ATTTTTATCGATATCGATAATATCCCGATATTTCCATCGAAATTTCCGTGTTTTTGGACTACCGATATTTCCGATATCACCGATATTTTAGACCTTGGTTGGTAG
**1650  GGACTACCGATATTTCCGATATCACCGATATTTTAGACCTTGGTTGGTAGATTTTTATCGATATCGATAATATCCCGATATTTCCATCGAAATTTCCGTGTTTTT
```
