# Supplementary material for: Defining the ‘HoneySweet’ insertion event utilizing NextGen sequencing and a de novo genome assembly of plum (Prunus domestica)
Source: Hortic Res. 2021 Jan 1;8:8. doi: 10.1038/s41438-020-00438-2 (PMC7775438; doi:10.1038/s41438-020-00438-2)
Supplement: Supplementary file 5 — Supplemental Table 1 [file 41438_2020_438_MOESM5_ESM.pdf]

Table S1. Junction sequences found in whole genome sequencing and the confirming PCR reactions.

	Junction[1]	Match BAC clone[2]	Junction sequence[3]	Confirming PCRs-Primer Pairs[4]
1	Plum::<br>42>[5]	BAC153	*TGATGCTCTTTTTTGGTTCTAATGAAGAACTAG*<br>ACTGAAGGCGGGAAACGACAATCTGATCAT	1F-NPT3R
2	<2743::<br>7276<	BAC153	*AAGAAACTTTATTGCCAAATGTTTGAAC*<br>GATC<br>GGGGAAATTCGAGCTCGGTAGCAATTCCCG	2F-Gus6823F<br>2R-NPT5'-1100
3	<4337::<br>8019<	BAC153	*CAACAAAGGGTAATATCGGGAAACCT*<br>TAGGCTCCGCCCCCCTGACGAGCATCACAA	3F-Bla8839R 3F-4R
*4	>10120/<br>10131::<br>68/79>	BAC153	*ATATTATTGAAGCATTTATCAGGGTTATTGTC*<br>TCATGAGCGGA<br>GAATTAAGGGAGTCACGTTATGACCCCCGC	4F-NPT649R 4F-NPT983R 4F-PPV5'3171R
5	<7562::<br>5943>		*TGCATGTGTCAGAGGTTTTCACCGTCATCACC*<br>GGTGGACGATATCACCGTGGTGACGCATGT	5F-GUS5R<br>5F-Bla3'-8839R 5F-Bla3'-9804<br>5R-Bla8839R 5R-Bla-9804R
6	>10177::<br>10288<		*AATAAACAAATAGGGGTTCCGC*<br>G<br>TACTGAATTCTTGAAGACGAAAGGGCCTCG	6F-pBR322-8032F<br>6R-GUS1441
7	>10304::<br>10217<		*TCTTCAAGAATTCAGTACATTAAAAACGTCCGC*<br>AATG<br>GTTTCTTAGACGTCAGGTGGCACTTTTCGG	7F-pBR322-7560F<br>7R-pBR322-8032F 7R-pBR322-7560F
8	<25::<br>Plum		TAAACATAATATTTTCGTTTGACAA<br>AAAC<br>*ACTGATAGTTTAAACTGAAGGCGGGAAACGAC*	8R-NPT983 8R-NPT649
9	Plum::<br>4917<	BAC207	*ACAGTTCATACAGAGTCTCTTACGACTCAATGA*<br>ATTCTTTCTTGGGATGCACCGTGCACGTGTG	9F-10R
10	<2961::<br>2834>	BAC207	*GTGGTCTCGGTATCTATCATAAACT T TACCTGG*<br>AGGGCCTGTGTTCGACAATAACAGACTAGA	10F-11R
11	>4917::<br>Plum	BAC207	*AGTTCATACAGAGTCTCTTACGACTCAATGA*<br>ATTCTTTCTTGGGATGCACCGTGCACGTGTG	10F-11R

[1]Junction is described by Plum if it is plum sequence and by a number which represents the location in the original vector, SFig1.

[2]Indicates if the junction was found in either of the two sequenced BAC clones.

[3]Sequence in italics is on the upstream side of the junction and the plain sequence is on the downside of junction. Sequence in between could be on either side of the junction.

[4]Indicates the primer pairs that gave a product confirming the junction. Primers are listed in Table S2)

[5]Direction of carrot indicates in the sequence is positive > or negative < strand
