# Supplementary material for: Defining the ‘HoneySweet’ insertion event utilizing NextGen sequencing and a de novo genome assembly of plum (Prunus domestica)
Source: Hortic Res. 2021 Jan 1;8:8. doi: 10.1038/s41438-020-00438-2 (PMC7775438; doi:10.1038/s41438-020-00438-2)
Supplement: Supplementary file 6 — Supplemetary Table 2 [file 41438_2020_438_MOESM6_ESM.pdf]

Table S2. Primer sequences used in confirming junction sequences in whole genome sequence of 'HoneySweet'.

Name	Primer Sequence
1F	GAAGAACTAGACTGAAGGCGGG
2F	GCATGTCTTGCGTTGATGAAGC
2R	AGGGAGGCAAACAATGAATCAACAAC
3F	TATCGGGAAACCTTAGGCTCC
3R	CGGAGCCTAAGGTTTCCCGATA
4F	TTGTCTCATGAGCGGAGAATTAAGG
4R	CCTTAATTCTCCGCTCATGAGACAA
5F	ACCGTCATCACCTGGGTGGA
5R	TCCACCCAGGTGATGACGGT
6F	GGGTTCCGCGTACTGAATTCTTG
6R	CAAGAATTCAGTACGCGGAACCC
7F	GTCCGCAATGGTTTCTTAGACG
7R	CGTCTAAGAAACCATTGCGGAC
8R	CGTTTGACAAAAAACTGATAG
9F	GCAAGGAAATGTGCGAGTTCTGT
10F	AACTTTACCTGGAGGGCCTGTG
10R	CACAGGCCCTCCAGGTAAAGTT
11R	CCCAAGAAAGAATTCATTGAGTCG
NPT3R	TTCAGTGACAACGTCGAGCACA
NPT649R	TTCAGTGACAACGTCGAGCACA
NPT983R	CCATGATATTCGGCAAGCAGGCAT
NPT5'-1100	GCTTCCTCGTGCTTTACGGTATC
PPV5’3171R	CGAAATGACTTCAACGACACCC
GUS1441	GATTTGGAAACGGCAGAG
GUS5R	AAATATTCCCGTGCACCTTGCG
Bla3'-8839R	AGGCAACTATGGATGAACG
Bla-9804R	TCGCCGCATACACTATTC
pBR322-7560F	TCGGTGATGACGGTGAAAACC
pBR322-8032F	CCTGACGAGCATCACAAAAATCG
