# Supplementary material for: Defining the ‘HoneySweet’ insertion event utilizing NextGen sequencing and a de novo genome assembly of plum (Prunus domestica)
Source: Hortic Res. 2021 Jan 1;8:8. doi: 10.1038/s41438-020-00438-2 (PMC7775438; doi:10.1038/s41438-020-00438-2)
Supplement: Supplementary file 7 — Supplementary Table 3 [file 41438_2020_438_MOESM7_ESM.pdf]

Table S3. Location of predicted insertion events and flanking genes in all plum scaffolds.

**Insertion 1 Upstream**[1]				2 carboxy-1,4 napthoquinone phytyltransferase			
Scaffold	Length	14-3-3 like protein[2]				Insert 1[3]	
		Plum gene ID[4]	Location[5]	Plum gene ID	Location	5' border	3' border
2675	652,684	Pd.00g722710	500678-502984	Pd.00g722720	505444-510730	512,344	512,378
1234	1,642,067	Pd.00g007460	1105256-1107562	Pd.00g007470	1109964-1115752	1,116,847	1,116,881
1429	1,885,080	Pd.00g1042760	608,906-611,211	Pd.00g1042770	613671-618960	620,936	620,971
1332	463,642	Pd.00g783580	38291-40583	Pd.00g783590	41729..48627	50,252	50,286
1650	2,171,811	Pd.00g1204540	2037140-2039446	Pd.00g1204550	2042068..2047156	2,048,827	2,048,861
**Insertion 1 Downstream**[6]							
Scaffold	Length	NCRNA		ABC transporter G family member like		phospho-2-dehydro-3-deoxyheptonate	
		Plum gene ID	Location	Plum gene ID	Location	Plum gene ID	Location
2675	652,684	Pd.00g722730	512,942-513,229	Pd.00g722740	550,641-556,129	Pd.00g722760	613,017-616,623
1234	1,642,067	Pd.00g007480	1117445-1117732	Pd.00g007490	1141277-1146786	Pd.00g007500	1,223,582-1,226,995
1429	1,885,080	Pd.00g1042780	656,687-657,090	Pd.00g1042790	658200-664262	Pd.00g1042820	708,108-711,521
1332	463,642	-		Pd.00g783600	70,837-75,341	-	
1650	2,171,811	-		Pd.00g1204560	2063904-2069231	Pd.00g1204590	2,129,673-2,132,233
**Insertion 2 Upstream**[7]							
Scaffold	Length	DMR6-LIKE OXYGENASE 2-like		Insert 2-Hairpin[8]		Insert 2-Hairpin[8]	
		Plum gene ID	Location	5' border	3' border	5' border	3' border
2675	652,684	Pd.00g722430	41421..42341	63,535	63,574		
1234	1,642,067	Pd.001g 007160	418131..419051	574,510	574,549	461,925	461,964
1234	1,642,067	Pd.001g 007190	539755..541132				
1429	1,885,080	Pd.001g 1042470	250429..251810	269,251	269,290	173,055	173,094
1429	1,885,080	Pd.00g1042430	145796..147161				
1650	2,171,811	Pd.00g1204450	1882422..1885089	1,891,212	1,891,173		
4359	638,531	Pd.001g 509790	268296..269677	287,100	287,139		
6796	1,062,804	Pd.001g864740	873165..875575	893,504	893,543		
4101	2,184,767	Pd.00g357610	248531..251198	257,301	257,262		
**Insertion 2 Downstream**							
Scaffold	Length	DMR6-LIKE OXYGENASE 2-like					
		Plum gene ID	Location				
2675	652,684	Pd.00g722440	66114..67514				
1234	1,642,067	Pd.001g 007170	466861..467285				
1234	1,642,067	Pd.00g007100.m01	579652..582272				
1429	1,885,080	Pd.001g 1042480	271627..272444				
1429	1,885,080	Pd.00g1042440	186445..187806				
1650	2,171,811	Pd.00g1204460	1899807..1901184				
4359	638,531	Pd.001g 509800	289476..290293				
6796	1,062,804	Pd.001g864750	901727..903121				
4101	2,184,767	Pd.00g357620	264638..266015				

[1]These are the scaffolds and their size that contain sequence homologous to the plum borders for insertion 1. The two flanking genes upstream are listed.

[2]The two flanking gene identities were determined by homologies in Genbank to genes with same annotation.

[3]The 5' and 3' border represent the nucleotide positions of the first plum base that abutts insert 1 sequence. Gaps between the two numbers represent deletion

[4]The plumID represents the gene ID given in the plum assembly found at the GDR website, https://www.rosaceae.org.

[5]Location are the coordinates for the gene in the designated Scaffold.

[6]The same scaffords and sizes as the upstream but the 3 flanking genes are downstream by location.

[7]The descriptions are the same as for insert 1 but now they are the flanking genes for insert 2, upstream and downstream.

[8]This is the location of insert 2 in the respective scaffolds. This is the 'hairpin' arrangement of the PPV-CP. Some of the scaffolds have two potentials sites.
