# Supplementary material for: Defining the ‘HoneySweet’ insertion event utilizing NextGen sequencing and a de novo genome assembly of plum (Prunus domestica)
Source: Hortic Res. 2021 Jan 1;8:8. doi: 10.1038/s41438-020-00438-2 (PMC7775438; doi:10.1038/s41438-020-00438-2)
Supplement: Supplementary file 8 — Supplementary Table 4 [file 41438_2020_438_MOESM8_ESM.pdf]

**Table S4. List of the Plum scaffolds that have any synteny with the peach Pp08 that overlaps with the two insertion events in 'HoneySweet'.**

| | | | | | | | Flanking Genes[6] | | | |
|---|---|---|---|---|---|---|---|---|---|---|
| | | | | | | Peach Gene[7] | Prupe.8G064500.1 | Prupe.8G064300.1 | Prupe.8G066600.1 | Prupe.8G066500.1 |
| | | | | | | Location in Pp08 | 9,557,545..9,563,216 | 9,487,742..9,492,470 | 9,839,014..9,840,380 | 9,834,136..9,835,889 |
| Scaffold[1] | Insertion 1[2] | Insertion2[3] | Insertion2[3] | Plum Synteny[4] | Peach Synteny[5] | Synteny Block ID[8] | Insertion 1 | Insertion 1 | Insertion 2 | Insertion 2 |
| 4892 | | | | 87,893-1,018,281 | 8,385,328-9,492,470 | pdoppB2610 | | Pd.00g1300790.m01 | | |
| 3648 | | | | 329,961-712,923 | 8,473,585-9,824,282 | pdoppB2087 | | | | |
| 1429 | 620,936 | 269,251 | 173,055 | 20,906-1,584,537 | 8,473,585-9,856,415 | pdoppB0738 | Pd.00g1042770.m01 | Pd.00g1042790.m01 | Pd.00g1042370.m01 Pd.00g1042430.m01 | Pd.00g1042380.m01 Pd.00g1042440.m01 |
| 1234 | 1,116,847 | 574,510 | 461,925 | 13,955-1,231,936 | 9,475,233-9,952,471 | pdoppB2208 | Pd.00g007470.m01 | Pd.00g007490.m01 | Pd.00g007160.m01 Pd.00g007190.m01 | Pd.001g 007170.m01 Pd.00g007200.m01 |
| 2675 | 512,344 | 63,535 | | 41,421-619,300 | 9,475,233-9,835,889 | pdoppB1469 | Pd.00g722720.m01 | Pd.00g722740.m01 | Pd.00g722430.m01 | Pd.00g722440.m01 |
| 1650 | 2,048,827 | 1,891,212 | | 113,498-2,135,024 | 9,475,233-10,539,990 | pdoppB0862 | Pd.00g1204550.m01 | Pd.00g1204560.m01 | Pd.00g1204450.m01 | Pd.00g1204460.m01 |
| 2491 | | | | 1,046,267-2,315,245 | 9,477,835-10,664,355 | pdoppB1384 | | Pd.00g125500.m01 | | |
| 1195 | | | | 1,715,331-2,979,226 | 9,477,835-10,664,355 | pdoppB1610 | | Pd.00g423490.m01 | | |
| 4359 | | 287,100 | | 9,640-567,380 | 9,622,797-9,952471 | pdoppB2379 | | | Pd.00g509790.m01 | Pd.00g509800.m01 |
| 4101 | | 257,301 | | 58,678-1,892,490 | 9,707,582-10,635,400 | pdoppB2279 | | | Pd.00g357610.m01 | Pd.00g357620.m01 |
| 1884 | | | | 34,744-585,139 | 9,810,765-10,121,166 | pdoppB0998 | | | | |
| 1428 | | | | 13,319-578,588 | 9,834,136-10,297,726 | pdoppB0735 | | | | Pd.00g476390.m01 |
| 6796 | | 893,504 | | 53,293-753,728 | 9,847,684-10,297,726 | pdoppB0339 | | | Pd.001g864740 | Pd.001g864750 |
| 267 | | | | 3,440,128-3,924,628 | 10,424,704-11,141,502 | pdoppB1465 | | | | |
| 3194 | | | | 1,288,759-1,952,169 | 10,424,704-11,141,502 | pdoppB1768 | | | | |
| 2373 | | | | 166,509-653,824 | 10,601,512-11,141,502 | pdoppB1275 | | | | |
| 5274 | | | | 102,918-765,961 | 10,611,481-11,236,100 | pdoppB2697 | | | | |
| 1332 | 50,252 | | | Pp01-274,708-427,210; Pp07-122,391-461,205 | Pp01-34403188-34490426 Pp07-15,974,851-16,173,775 | pdoppB0407 pdoppB0408 | Pd.00g783590 | Pd.00g783600 | | |

[1]Scaffold that has synteny with the peach genome covering the homologous region for either insertion 1 or insertion 2.

[2]Location of insertion1 in the scaffold as defined by the plum border sequences flanking insert 1.

[3]Location of insertion2 in the scaffold as defined by the plum border sequences flanking insert 2.

[4]Region of plum scaffold that matches with synteny to the peach sequence in next column.

[5]Peach region on Pp08 with synteny to plum. Colored fill highlights scaffolds that are related but do not have any matching flanking sequence to the insertions. The genes present are part of [illegible]

[6]Flanking genes are peach genes that flank homologous insertion sites. The identities are the same as the flanking plum genes,2 carboxy-1,4 napthoquinone phytyltransferase and ABC transporter G family for insertion 1 and DMR6-LIKE OXYGENASE 2-like for insertion 2.

[7]Location of bases for the matching peach genes

[8]The block ID is generated by the Synteny program at GDR and identifies the peach to plum synteny regions.
