# Supplementary material for: Defining the ‘HoneySweet’ insertion event utilizing NextGen sequencing and a de novo genome assembly of plum (Prunus domestica)
Source: Hortic Res. 2021 Jan 1;8:8. doi: 10.1038/s41438-020-00438-2 (PMC7775438; doi:10.1038/s41438-020-00438-2)
Supplement: Supplementary file 9 — Supplementary Table 5 [file 41438_2020_438_MOESM9_ESM.pdf]

Table S5. Gene expression for genes flanking insertion 1 in 'HoneySweet' was compared to that of 'Stanley' to determine if changes in expression was detected.

		Scaffold1234 (121,739)[1]					Scaffold2675 (115,945)						Scaffold1332 (85,887)					Scaffold1429 (102,615)							Scaffold1650 (95,093)					
	Tree #[2]	Pd.00g007460	Pd.00g0074703	Pd.00g007480	Pd.00g007490	Pd.00g007500	Pd.00g722710	Pd.00g722720	Pd.00g722730	Pd.00g722740	Pd.00g722750	Pd.00g722760	Pd.00g783580	Pd.00g783590	Pd.00g783600	Pd.00g783610	Pd.00g783620	Pd.00g1042760	Pd.00g1042770	Pd.00g1042780	Pd.00g1042790	Pd.00g1042800	Pd.00g1042810	Pd.00g1042820	Pd.00g1204540	Pd.00g1204550	Pd.00g1204560	Pd.00g1204570	Pd.00g1204580	Pd.00g1204590
Leaf[3]																														
HS[4]	83	0.1	0.5	-	-	-	0	0	0	0	0.3935	2.57	-	-	-	-	1.7	-	-	-	-	-	0.4	-	-	7.0	-	0.9	0.2	-
	87	-	0.3	-	-	-	0	0	0	0	0.4736	1.96	-	0.1	8.5	-	-	-	-	-	2.5	-	0.7	-	-	3.7	0.1	2.2	0.0	-
	91	-	0.6	-	-	-	0	0	0	0	0.2911	1.23	-	-	-	-	4.5	-	-	-	0.2	-	0.7	-	-	5.4	-	0.8	0.0	-
	159	0.1	0.4	-	-	-	0	0	0	0	0.1705	[illegible]	-	-	-	0.2	2.0	-	-	-	-	-	0.2	-	-	5.6	-	0.6	0.3	-
	163	-	0.6	-	-	-	0	0	0	0	0.1615	1.85	-	-	-	-	2.5	-	-	-	-	-	0.1	-	-	7.7	-	1.0	0.2	-
	167	-	0.3	-	-	-	0	0	0	0	0.0662	1.24	-	-	-	-	0.2	-	-	-	-	-	0.3	-	-	2.9	0.3	1.1	0.0	-
	171	-	0.5	-	-	-	0	0	0	0	0.0653	2.32	-	-	-	-	0.1	-	-	-	0.0	0.1	0.4	-	-	4.2	1.0	3.9	-	-
	175	-	0.9	-	-	-	0	0	0	0	1.7064	1.77	-	-	-	-	1.0	-	-	-	0.0	-	1.2	-	-	5.3	0.1	3.1	1.6	-
ST[5]	29	-	0.3	-	-	-	0	0	0	0	0.0939	3.42	-	0.4	0.1	-	6.0	-	0.1	-	-	-	0.1	-	-	3.1	-	0.2	-	-
	41	0.1	0.2	-	-	-	0	0	0	0	0	3.54	-	0.3	-	-	5.6	-	0.1	-	0.0	0.6	0.1	-	-	4.9	-	0.1	0.0	-
	45	-	0.4	-	-	-	0	0	0	0	0.075	3.12	-	0.3	-	-	6.0	-	0.1	-	0.0	0.5	0.1	-	-	0.2	-	-	1.9	-
	61	0.2	0.5	-	-	-	0	0	0	0	1.9299	1.33	-	0.4	-	-	4.3	-	0.1	-	-	3.5	0.3	-	-	4.5	-	2.1	1.2	-
	67	0.1	0.1	-	-	-	0	0	0	0	0.025	2.92	-	-	0.7	-	-	-	0.0	-	0.8	2.0	0.1	-	-	1.0	0.5	3.1	0.0	-
	71	0.1	0.1	-	-	-	0	0	0	0	1.7901	1.87	-	0.2	0.1	-	14.6	-	0.1	-	0.0	1.9	0.7	-	-	1.0	-	1.3	0.3	-
ST Inf[6]	33	-	0.7	-	-	-	0	0	0	0	0.0359	5.5	-	1.0	-	-	4.0	-	0.3	-	-	1.7	-	-	-	5.9	-	0.3	-	-
	37	-	0.6	-	-	-	0	0	0	0	0.0747	4.5	-	1.0	-	-	2.9	-	0.7	-	0.0	1.4	0.1	-	-	7.9	-	0.1	0.0	-
	55	-	0.7	-	-	-	0	0	0	0	0.1964	6.79	-	0.5	0.1	-	8.0	-	0.2	-	0.2	0.3	0.4	-	-	4.9	-	0.8	-	-
	63	0.2	0.3	-	-	-	0	0	0	0	0.9481	4.8	-	0.5	0.4	-	4.9	-	-	-	0.7	0.6	0.9	-	-	2.7	-	1.2	-	-
Fruit[7]																														
HS	51	-	0.0	-	-	-	0	0	0	0	0.9249	0.84	-	-	-	-	0.1	-	-	-	-	-	0.3	-	-	0.0	-	0.9	0.2	-
	95	0.1	-	-	-	-	0	0	0	0	0.4607	2.43	-	-	-	-	0.1	-	-	-	-	-	0.4	-	-	0.0	-	0.6	0.8	-
	99	-	-	-	-	-	0	0	0	0	0.3981	1.98	-	-	-	-	0.1	-	-	-	0.0	-	0.6	-	-	0.0	-	1.1	0.7	-
	103	-	-	-	-	-	0	0	0	0	0.7205	1.98	-	-	-	0.1	0.1	-	-	-	-	-	0.5	-	-	0.0	-	1.2	1.1	-
	119	0.1	-	-	-	-	0	0	0	0	2.8086	1.06	-	-	-	-	-	-	-	-	0.0	-	6.5	-	-	-	-	6.1	1.3	-
	131	-	0.0	-	-	-	0	0	0	0	[illegible]	1.29	-	-	-	-	0.1	-	-	-	0.0	-	3.8	-	-	0.1	-	4.0	1.3	-
	143	-	-	-	-	-	0	0	0	0	0.7954	1.44	-	-	-	-	0.5	-	-	-	-	-	0.5	-	-	0.1	-	1.0	0.1	-
	147	-	-	-	-	-	0	0	0	0	0.6413	0.9	-	-	-	-	-	-	-	-	-	-	0.4	-	-	-	-	0.8	0.1	-
ST	107	-	-	-	-	-	0	0	0	0	0.6471	6.28	-	-	-	0.4	1.2	-	-	-	-	0.6	0.7	-	-	-	-	0.7	0.0	-
	111	-	-	-	-	-	0	0	0	0	0.4904	6.28	-	-	-	0.3	0.5	-	-	-	-	0.5	0.6	-	-	-	-	0.3	0.0	-
	115	0.1	-	-	-	-	0	0	0	0	0.8197	6.73	-	-	-	-	0.7	-	-	-	-	0.3	0.5	-	-	-	-	0.7	0.1	-
	135	-	-	-	-	-	0	0	0	0	1.6768	1.58	-	-	0.1	0.4	0.2	-	-	-	-	0.7	1.2	-	-	0.1	-	1.3	0.0	-
	139	-	-	-	-	-	0	0	0	0	0.2924	8.95	-	-	-	-	0.4	-	-	-	-	0.5	0.7	-	-	0.1	-	0.8	0.0	-
	151	0.1	-	-	-	-	0	0	0	0	0.555	4.73	-	-	-	-	1.8	-	-	-	-	0.1	0.5	-	-	0.1	-	0.3	-	-
ST Inf	47	-	-	-	-	-	0	0	0	0	0.9361	3.47	-	-	-	0.2	1.2	-	-	-	-	0.7	0.8	-	-	-	-	1.0	0.3	-
	123	0.1	-	-	-	-	0	0	0	0	0.7813	8.33	-	-	-	0.4	1.1	-	-	-	-	0.7	0.5	-	-	0.0	-	0.7	0.0	-
	127	-	-	-	-	-	0	0	0	0	0.6376	8.91	-	-	-	0.5	1.1	-	-	-	-	0.5	0.4	-	-	-	-	0.5	0.1	-
	155	0.2	-	-	-	-	0	0	0	0	0.5879	2.66	-	-	-	0.2	0.3	-	-	-	-	0.3	0.5	-	-	0.1	-	0.4	0.3	-

Scale[8]							
0	0.5	1	2	4	8	10	15

[1]Scaffolds that have sequence matching the plum border sequence of insert 1 are named here with the total nucleotides that cover the 5-7 flanking genes in common with the other scaffolds

[2]Tree number represents individual trees from which a leaf and a fruit library was made from RNA and sequenced.

[3]This the data from the individual tree leaf tissue RNAseq, values are in Transcripts Per Million (TPM).

[4]'HoneySweet'

[5]'Stanley'

[6]'Stanley' infected with Plum pox virus

[7]This is the data from the individual tree fruit tissue RNAseq--values are in Transcripts Per Million (TPM).

[8]Scale that color codes the ranges of TPMs from 0 to 15.

A line indicates the position of the insertion.
