# Supplementary material for: Defining the ‘HoneySweet’ insertion event utilizing NextGen sequencing and a de novo genome assembly of plum (Prunus domestica)
Source: Hortic Res. 2021 Jan 1;8:8. doi: 10.1038/s41438-020-00438-2 (PMC7775438; doi:10.1038/s41438-020-00438-2)
Supplement: Supplementary file 10 — Supplementary Table 6 [file 41438_2020_438_MOESM10_ESM.pdf]

Table S6. Gene expression for genes flanking insertion 2 in 'HoneySweet' was compared to that of 'Stanley' to determine if changes in expression was detected.

		Scaffold1234 (311,772)[1]							Scaffold4101 (50,061)				Scaffold4359 (83,552)				Scaffold2675 (34,846)				Scaffold6796 (76,875)				Scaffold1429 (143,729)								Scaffold1650 (56,162)			
	Tree #[2]	Pd.00g007150	Pd.00g007160	Pd.00g007170	Pd.00g007180	Pd.00g007190	Pd.00g007200	Pd.00g007210	Pd.00g357600	Pd.00g357610	Pd.00g357620	Pd.00g357630	Pd.00g509780	Pd.00g509790	Pd.00g509800	Pd.00g509810	Pd.00g722420	Pd.00g722430	Pd.00g722440	Pd.00g722450	Pd.00g864730	Pd.00g864740	Pd.00g864750	Pd.00g864760	Pd.00g1042420	Pd.00g1042430	Pd.00g1042440	Pd.00g1042450	Pd.00g1042460	Pd.00g1042470	Pd.00g1042480	Pd.00g1042490	Pd.00g1204440	Pd.00g1204450	Pd.00g1204460	Pd.00g1204470
Leaf[3]																																				
HS[4]	83	-	0.2	0.2	-	-	-	8.7	-	-	-	-	-	-	-	-	-	-	-	-	0.0	-	-	-	-	-	0.4	-	-	-	-	-	-	-	-	-
	87	-	-	0.2	-	-	-	8.2	-	-	-	0.1	-	0.1	-	-	-	-	-	-	0.1	-	-	-	-	-	0.3	-	-	-	-	-	-	-	-	0.1
	91	-	-	0.7	-	-	-	5.2	-	-	-	-	-	0.0	-	-	-	-	-	-	-	-	-	-	-	-	0.1	-	-	-	-	-	-	-	-	-
	159	-	-	0.6	-	-	-	15.9	-	-	-	0.1	-	0.1	-	-	-	-	-	-	0.1	-	-	-	-	-	1.3	-	-	-	-	-	-	-	-	-
	163	-	-	0.9	-	0.1	-	16.1	-	-	-	-	-	-	-	-	-	-	-	-	-	-	-	-	-	-	1.2	-	-	-	-	-	-	-	-	-
	167	-	0.1	0.3	-	-	-	4.9	-	-	-	0.1	-	-	-	-	-	-	-	-	-	-	-	-	-	-	0.7	-	-	-	-	-	-	-	-	-
	171	-	-	0.5	-	-	-	9.3	-	-	-	-	-	0.6	-	-	-	-	-	-	0.7	-	-	-	-	-	0.5	-	-	-	-	-	-	-	-	-
	175	-	-	0.6	-	-	-	3.7	-	-	-	-	0.1	-	-	-	-	-	-	-	-	-	-	-	-	-	-	-	-	-	-	-	-	-	-	-
ST[5]	29	-	0.1	1.0	-	-	-	14.0	-	-	-	0.7	-	-	-	-	-	-	-	-	-	-	-	-	-	0.1	0.9	-	-	-	-	-	-	-	-	-
	41	-	-	0.5	-	0.1	-	11.5	-	-	-	0.3	-	-	-	-	-	-	0.2	0.1	0.3	-	0.1	-	-	-	1.2	-	-	-	-	-	-	-	-	-
	45	-	0.1	0.7	-	-	-	14.3	-	-	-	0.1	-	-	-	-	-	-	-	-	0.1	-	-	-	-	0.1	0.8	-	-	-	-	-	-	-	-	-
	61	-	-	-	-	-	-	[illegible]	-	-	-	0.2	0.0	0.0	-	-	-	-	-	-	0.3	-	-	-	-	-	0.9	-	-	-	-	-	-	-	-	-
	67	-	0.2	1.6	-	-	-	15.6	-	-	-	0.1	-	-	-	-	-	-	-	-	0.0	-	-	-	-	0.1	1.5	-	-	-	-	-	-	-	-	-
	71	-	-	-	-	-	-	4.7	-	-	-	0.1	-	-	-	-	-	-	-	-	-	-	0.1	-	-	-	[illegible]	-	-	-	-	-	-	-	-	0.1
ST Inf[6]	33	-	-	1.3	-	-	-	9.8	-	-	-	0.5	-	-	-	-	-	-	-	-	0.1	0.1	-	-	-	-	1.0	-	-	-	-	-	-	-	-	-
	37	-	-	0.5	-	-	-	8.9	-	-	-	0.5	-	-	-	-	-	-	-	-	0.1	-	-	-	-	0.2	1.3	-	-	-	-	-	-	-	-	-
	55	-	-	-	-	-	-	[illegible]	-	-	-	0.8	-	-	-	-	-	-	-	-	-	-	-	-	-	-	1.3	-	-	-	-	-	-	-	-	-
	63	-	-	0.8	-	-	-	7.3	-	-	-	0.6	-	-	-	-	-	-	-	-	0.2	-	-	-	-	-	1.1	-	-	-	-	-	-	-	-	-
Fruit[7]																																				
HS	51	-	-	0.8	-	-	-	1.9	-	-	-	0.1	0.2	0.1	-	-	-	-	-	-	0.5	-	-	-	-	-	1.6	-	-	-	-	-	-	-	-	-
	95	-	-	0.6	-	-	-	0.9	-	-	-	0.1	0.1	0.1	-	-	-	-	-	-	0.4	-	-	-	-	-	0.4	-	-	-	-	-	-	-	-	-
	99	-	0.1	0.2	-	-	-	0.7	-	-	-	-	0.1	-	-	-	-	-	-	-	0.1	-	-	-	-	-	0.1	-	0.2	-	-	-	-	-	-	-
	103	-	-	0.6	-	-	-	0.2	-	-	-	0.1	0.0	-	-	-	-	-	-	-	0.1	-	-	-	-	-	0.1	-	-	-	-	-	-	-	-	-
	119	-	-	0.2	-	0.0	-	0.0	-	-	-	-	0.1	-	-	-	-	-	-	-	-	-	0.0	-	-	-	0.1	-	-	-	-	-	-	-	-	-
	131	-	-	0.2	-	-	-	0.2	-	-	-	0.1	0.1	-	-	-	-	-	-	-	0.1	-	-	-	-	-	0.2	-	-	-	-	-	-	-	-	-
	143	-	-	[illegible]	-	-	-	[illegible]	-	-	-	0.2	0.3	0.4	-	-	-	-	-	-	0.4	-	-	-	-	-	0.9	-	-	-	-	-	-	-	-	-
	147	-	-	-	-	-	-	0.2	-	-	-	0.1	0.1	-	-	-	-	-	-	-	0.1	-	-	-	-	-	0.1	-	-	-	-	-	-	-	-	-
ST	107	-	0.1	0.3	-	-	-	1.6	-	-	-	0.4	-	-	-	-	-	-	-	-	10.2	-	-	-	-	1.4	0.6	-	-	-	-	-	-	-	-	-
	111	-	-	-	-	-	-	0.5	-	-	-	0.2	-	-	-	-	-	-	-	-	21.0	-	-	-	-	1.4	0.8	-	-	-	-	-	-	-	-	-
	115	-	-	0.4	-	-	-	1.4	-	-	-	0.2	-	-	-	-	-	-	-	-	7.7	-	-	-	-	1.5	0.8	-	-	-	-	-	-	-	-	-
	135	-	-	0.7	-	0.1	-	4.5	-	-	-	0.2	0.1	-	-	-	-	-	-	-	0.2	-	-	-	-	0.1	7.1	-	-	-	-	-	-	-	-	-
	139	-	-	0.5	-	-	-	1.0	-	-	-	0.9	-	-	-	-	-	-	-	-	17.6	-	-	-	-	0.1	0.6	-	-	-	-	-	-	-	-	-
	151	-	-	-	-	-	-	4.1	-	-	-	0.4	0.1	-	-	-	-	-	-	-	[illegible]	-	-	-	-	0.1	1.1	-	-	-	-	-	-	-	-	-
ST Inf	47	-	-	-	-	-	-	0.1	-	-	0.7	-	-	-	-	-	-	-	-	-	1.4	-	-	-	-	0.2	0.1	-	-	-	-	-	-	-	-	-
	123	-	-	-	-	-	-	1.7	-	-	-	0.4	0.0	-	-	-	-	-	-	-	0.4	-	-	-	-	0.6	0.5	-	-	-	-	-	-	-	-	-
	127	-	-	-	-	-	-	0.7	-	-	-	0.7	0.1	-	-	-	-	-	-	-	0.2	-	-	-	-	0.3	0.7	-	-	-	-	-	-	-	-	-
	155	-	-	0.3	-	-	-	0.1	-	-	-	0.2	-	-	-	-	-	-	-	-	0.1	-	-	-	-	-	-	-	-	-	-	-	-	-	-	-

Scale[8]	-	0.4	2.0	5.0	10.0	21.0

[1]Scaffolds that have sequence matching the plum border sequence of insert 1 are named here with the total nucleotides that cover the 5-7 flanking genes in common with the other scaffolds

[2]Tree number represents individual trees from which a leaf and a fruit library was made from RNA and sequenced.

[3]This the data from the individual tree leaf tissue RNAseq, values are in Transcripts Per Million (TPM).

[4]'HoneySweet'

[5]'Stanley'

[6]'Stanley' infected with Plum pox virus

[7]This is the data from the individual tree fruit tissue RNAseq--values are in Transcripts Per Million (TPM).

[8]Scale that color codes the ranges of TPMs from 0 to 15.

A line indicates the position of the insertion.
