# Supplementary material for: Defining the ‘HoneySweet’ insertion event utilizing NextGen sequencing and a de novo genome assembly of plum (Prunus domestica)
Source: Hortic Res. 2021 Jan 1;8:8. doi: 10.1038/s41438-020-00438-2 (PMC7775438; doi:10.1038/s41438-020-00438-2)
Supplement: Supplementary file 11 — Supplementary Table 7 [file 41438_2020_438_MOESM11_ESM.pdf]

Table S2. - BUSCO copy number analysis summary

Number of Gene copies	Number of genes identified Phased[1]
1	76
2	95
3	210
4	347
5	636
>=6	30

[1]The number of genes in the BUSCO single genes that have each each
