# Supplementary material for: Defining the ‘HoneySweet’ insertion event utilizing NextGen sequencing and a de novo genome assembly of plum (Prunus domestica)
Source: Hortic Res. 2021 Jan 1;8:8. doi: 10.1038/s41438-020-00438-2 (PMC7775438; doi:10.1038/s41438-020-00438-2)
Supplement: Supplementary file 12 — Supplementary Table 8 [file 41438_2020_438_MOESM12_ESM.pdf]

Table S4. Phased plum genome: Sequencing libraries.

Library type	Insert size	Reads	Number of Libraries produced	Approximate depth (coverage)
PCR-free PE library (PE250X2)	450-470bp	250bpX2	1	X84
PCR-free PE library (PE150X2)	700- 800bp	150bpX2	1	X44
MP (Nextera™ MP Gel Plus)	2-4kbp	150bpX2	1	X29
MP (Nextera™ MP Gel Plus)	5-7kbp	150bpX2	1	X24
MP (Nextera™ MP Gel Plus)	8-10kbp	150bpX2	1	X29
10X genomics™ Chromium™	N/A	150bpX2	1	X55
